# Scalable fabrication of transparent sodium calcium silicate glass-ceramics by structural engineering overcoming anisotropy barriers

**DOI:** 10.1038/s41467-026-75474-y

**Published:** 2026-07-10

**Authors:** Weifan Liao, Yunlan Guo, Kaiwen Hu, Jong Heo, Xiujian Zhao, Jian Ruan, Chao Liu

**Affiliations:** 1 State Key Laboratory of Advanced Glass Materials, Wuhan, China; 2 Silicate Materials Engineering Research Center, Wuhan University of Technology, Wuhan, China; 3 State Key Laboratory of Silicate Materials for Architectures, Wuhan University of Technology, Wuhan, China

**Keywords:** Ceramics, Glasses, Biomedical materials, Mechanical properties

## Abstract

Transparent glass-ceramics with high crystallinity offer many attractive optical, mechanical, and biomedical properties. However, for glass-ceramics containing crystals with thermal expansion anisotropy, excessive crystallization induces severe microcracking and consequent loss of transparency. Here, we employ structural engineering in the sodium calcium silicate system to mitigate thermal expansion anisotropy through Zr^4+^ substitution at Ca^2+^ sites in Na_4_Ca_4_Si_6_O_18_ crystal and stabilize the crystallized high-temperature polymorph. This strategy overcomes microcrack barriers, enabling large-sized glass-ceramics with high crystallinity ( ~ 93 wt%) and >84% visible transmittance. Validated for biomedical implants (100% cell viability and bioactivity) and fire-resistant windows, the work expands the application scope of the traditional alkali-silicate system towards highly crystalline transparent glass-ceramics for advanced functional uses.

## Abstract (web summary)

Researchers achieve scalable production of large, highly crystalline transparent glass-ceramics via Zr4+ substitution, which mitigates thermal expansion anisotropy and stabilizes high-temperature phases for practical application.

## Introduction

Transparent glass-ceramics (TGCs) constitute a unique class of inorganic materials that integrate the processing flexibility of glasses with the functional advantages of crystalline phases^1^. By controlling the nucleation and growth of crystals within a glass matrix, TGCs can achieve a wide range of microstructures— from isolated nanocrystals to almost fully crystallized bodies—while retaining high optical transparency^1,2^. This unique combination has enabled TGCs to outperform conventional glasses in mechanical strength, thermal stability, and chemical durability, and to offer tailorable coefficients of thermal expansion (including near-zero or negative values) that are essential for precision optical components, telescope mirrors, cooktop panels, and fire-resistant windows^3^. In the photonics domain, TGCs containing functional crystals (e.g., fluorides, semiconductors, or nonlinear optical phases) serve as efficient spectral converters, laser gain media, scintillators, and upconversion materials, often exhibiting luminescence properties that surpass those of their parent glasses^4–6^. Moreover, certain TGC compositions are bioactive and machinable, making them attractive for bone implants and dental restorations^7^. Despite these remarkable achievements, the practical implementation of TGCs in large-scale and high-performance applications remains constrained by several fundamental challenges. Most commercially successful TGCs—notably lithium aluminosilicate (LAS) and magnesium aluminosilicate (MAS) systems—rely on the precipitation of nanosized crystals to minimize light scattering according to Rayleigh-Gans theory, and their crystalline volume fraction should be tailored to avoid microcracking caused by thermal expansion mismatch between the crystal and the residual glass^3,8^.

Among all alkali-containing glass families, sodium calcium silicate (SCS) glasses are by far the most abundant, accounting for over 80% of global glass output, and are extensively used in architecture, automotive manufacturing, and consumer goods. Converting the commodity of SCS glass into transparent, highly crystalline glass-ceramics would offer immense economic and environmental benefits, yet this goal has remained elusive for decades due to microcracking the tendency and consequent loss of transparency. The specific composition of 1Na_2_O·2CaO·3SiO_2_ emerged as an ideal prototype, exhibiting homogeneous volume nucleation^9–12^ and potential for congruent crystallization of low-birefringence Na_4_Ca_4_Si_6_O_18_ crystals^13–15^. Berthier et al.^14^ were able to convert into TGCs containing Na_4 + 2*x*_Ca_4 − *x*_[Si_6_O_18_] (0 ≤ *x* ≤ 1) solid solution crystals^16,17^ with ~97% crystallinity, 5–7 µm grain size, and <60% transmittance at 400 nm. However, thermal expansion anisotropy along the a- and c-axes of Na_4_Ca_4_Si_6_O_18_ crystals causes spontaneous microcracking and concurrent transparency reduction when crystallinity exceeds 97%^14,18^. Although the phase is non-cubic, its birefringence is low^13–15^; therefore, mitigating this a/c-axis thermal expansion anisotropy is essential for obtaining highly crystalline transparent SCS glass-ceramics.

**Fig. 1:**
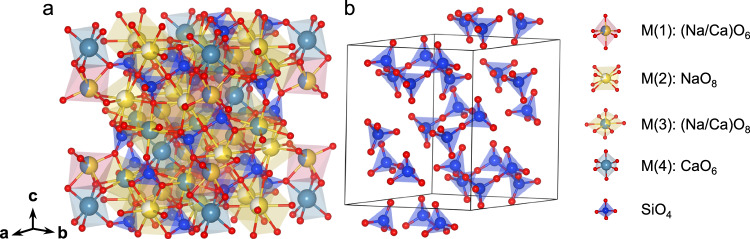
Crystal structure of Na_4_Ca_4_Si_6_O_18_ (PDF#79-1085). **a** Unit-cell structure visualization showing M-site cation coordination polyhedra. **b** Atomic configuration of [Si_6_O_18_]^12−^ rings demonstrating the planar hexagonal symmetry and corner-sharing [SiO_4_] connectivity.

Structurally, Na_4_Ca_4_Si_6_O_18_ belongs to lovozerite-group minerals with general formula A_3_B_3_C_2_MSi_6_O_12_O_6-*y*_(OH)_*y*_·*n*H_2_O (0 ≤ *y* ≤ 6; *n* = 0–1)^19–21^. The M sites are exclusively occupied by Zr^4+^, Ti^4+^, Fe^3+^, and Ca^2+^, while the A and C sites contain mixed Ca^2+^/Na^+^. In high-temperature Na_4_Ca_4_Si_6_O_18_, these cation sites are further divided into four distinct types^16^: six-coordinated M(1) sites accommodate Na^+^ and Ca^2+^, eight-coordinated M(2) sites host Na^+^ and vacancies, eight-coordinated M(3) sites contain Na^+^ and Ca^2+^, and six-coordinated M(4) sites are exclusively occupied by Ca^2+^ ions (Fig. 1a). The structure consists of puckered six-membered silicate rings stacked in cubic close-packing, interconnected through Na/Ca polyhedra (Fig. 1b)^16^. Due to weak cation-oxygen bonding compared to the rigid silicate framework, thermal expansion perpendicular to the silicate ring is substantially lower than parallel to it^16,22^—behavior analogous to ring silicates^23^. The high-temperature polymorph (trigonal, space group $R\bar{3}m$, isostructural with Na_6_Ca_3_Si_6_O_18_), exhibits thermal expansion coefficients of *α*_a_ = 11.6 × 10^−6^ K^−1^ and *α*_c_ = 7.86 × 10^−6^ K^−1^, while the low-temperature form (trigonal, P3_1_21 or P3_2_21) shows *α*_a_ = 14.2 × 10^−6^ K^−1^ and *α*_c_ = 6.17 × 10^−6^ K^−1^^16^. It is clear that the high-temperature phase demonstrates significantly reduced thermal expansion anisotropy. Strategic substitution of weakly bonded cations with stronger alternatives while simultaneously stabilizing the high-temperature polymorph should effectively mitigate thermal expansion anisotropy, enabling highly crystalline transparent SCS glass-ceramic fabrication.

Herein, we overcome a long-standing challenge in the fabrication of transparent and highly crystalline glass-ceramics by employing a structural engineering approach that mitigates microcracking from thermal expansion anisotropy. In glasses with compositions approximating Na_2_O·2CaO·3SiO_2_ modified with ZrO_2_ and B_2_O_3_, partial substitution of [CaO_6_] octahedra by [ZrO_6_] polyhedra effectively counteracts thermal expansion anisotropy. This structural optimization yields SCS glass-ceramics featuring a grain size of ~30 µm and visible-range transmittance of >84% at 400 nm with high crystallinity (~93 wt%). These results establish a scalable pathway to convert commercial SCS glass into high-performance TGCs for biological and high-temperature applications.

## Results

### Fabrication and structure characterization

**Fig. 2:**
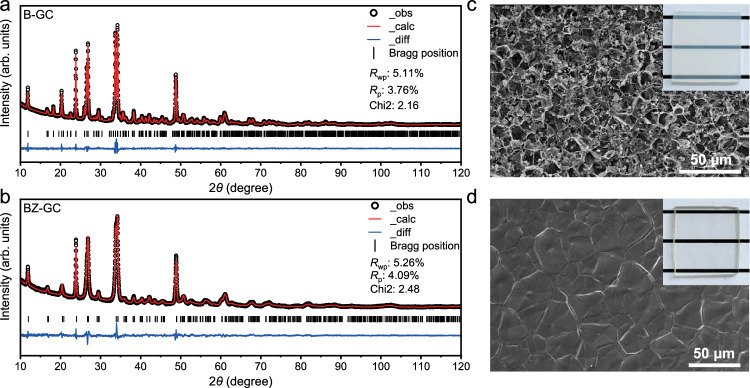
Structural and microstructural characterization of B-GC and BZ-GC. Rietveld refinement profiles of powder X-ray diffraction patterns for **a** B-GC and **b** BZ-GC samples. FE-SEM secondary electron micrographs and corresponding digital photographs (insets) of **c** B-GC and **d** BZ-GC samples. B-GC sample was prepared by 600 °C/20 h nucleation and 670 °C/20 h crystallization, and BZ-GC sample was prepared by 630 °C/20 h nucleation and 720 °C/20 h crystallization. Sizes of the specimens in (**c**, **d**) are ~1 cm × 1 cm. Source data of Fig. 1a–d are provided as a Source data file.

We optimized SCS glass compositions based on Berthier et al.^14^ and our previous work^24^. Two variants with identical base composition (49.27SiO_2_-34.48CaO-16.25Na_2_O in mol.%) were designed: one with B_2_O_3_ addition (B glass) and another with B_2_O_3_/ZrO_2_ co-doping (BZ glass). Detailed preparation methods are provided in the “Methods” section. Differential scanning calorimetry (DSC) revealed glass transition temperature (*T*_g_) and crystallization temperature (*T*_c_) for B glass (*T*_g_ = 590 °C, *T*_c_ = 678 °C) and BZ glass (*T*_g_ = 598 °C, *T*_c_ = 719 °C; Supplementary Fig. 1). Crystallization protocols comprised: B glass nucleated at 600 °C for 20 h and crystallized at 670 °C for 20 h; BZ glass nucleated at 630 °C for 20 h and crystallized at 720 °C for 20 h. Both systems achieved near-complete crystallization (Fig. 2a, b). Rietveld refinement analysis identified distinct crystalline phases: crystallized B glass contained crystalline phase corresponding to the low-temperature Na_4_Ca_4_Si_6_O_18_ (Trigonal P3_1_21/P3_2_21, PDF#78-364; *a* = *b* = 10.475 Å, *c* = 13.133 Å, *V* = 1248.4 Å^3^, *Z* = 3; Fig. 2a), consistent across temperatures (Supplementary Fig. 2). BZ glass formed crystalline phase closely matching the high-temperature polymorph of Na_4_Ca_4_Si_6_O_18_ (Trigonal $R\overline{3}{{{\rm{m}}}}$, PDF#79-1085; *a* = *b* = 10.476 Å, *c* = 13.061 Å, *V* = 1241.6 Å^3^, *Z* = 3; Fig. 2b). This phase difference dictated optical properties. B glass-ceramic (B-GC) maintained a translucent quality (Fig. 2c inset), characterized by irregular grain morphology with chemically etched surfaces and discontinuous grain boundaries, as evidenced in Fig. 2c. In contrast, BZ glass-ceramic (BZ-GC) achieved remarkable optical transparency (Fig. 2d inset) through close-packed polygonal grains with defined boundaries (Fig. 2d). Transmission electron microscopy (TEM) observation of the BZ-GC specimen revealed clear grain boundaries with sharp interfacial contrast and no obvious presence of a continuous glass phase (Supplementary Fig. 3a, b). High-resolution transmission electron microscopy (HR-TEM) imaging and corresponding fast Fourier transformation patterns (Supplementary Fig. 3c–e) further confirmed that the grains are highly crystalline and structurally consistent with the high-temperature Na_4_Ca_4_Si_6_O_18_ polymorph. Quantitative phase analysis via Rietveld refinement of X-ray diffraction (XRD) patterns, using 30 wt% rhombohedral α-Al_2_O_3_ powder as an internal standard (Supplementary Fig. 4), yielded crystalline fractions of approximately 90 wt% for B-GC and 93 wt% for BZ-GC. Owing to pronounced beam-sensitivity (charging and damage) caused by the material’s insulating nature and high alkali content, energy-dispersive X-ray spectroscopy (EDS) mapping (Supplementary Fig. 3f) could not conclusively detect a thin glass phase at the grain boundaries. However, time-of-flight secondary ion mass spectrometry (TOF-SIMS) mapping (Supplementary Fig. 5a) and laser ablation inductively coupled plasma mass spectrometry (LA-ICP-MS) analysis (Supplementary Fig. 5b) suggested that the residual glass phase, enriched in B and Zr, is likely distributed along the grain boundaries.

### Boron coordination and silicate network evolution

**Fig. 3:**
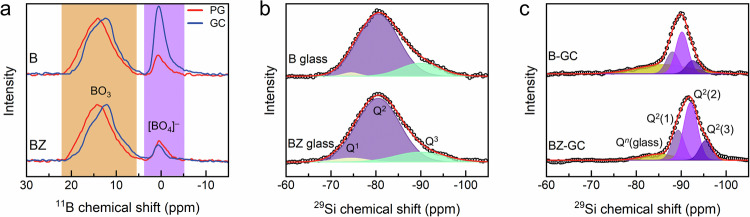
Boron coordination and silicate network evolution during crystallization. **a** ^11^B MAS NMR spectra of B and BZ precursor glasses (PG), B-GC and BZ-GC (GC), showing distinct chemical shifts for trigonal [BO_3_] (yellow region) and tetrahedral [BO_4_]^‒^ (purple region). ^29^Si MAS NMR spectra tracking structural evolution: **b** B and BZ precursor glasses showing broad Q^*n*^ (*n* = 1, 2, 3, from left to right) distribution, and **c** B-GC and BZ-GC exhibiting sharp Q^2^ (*i*, *i* = 1, 2, 3) distribution and broad Q^*n*^ in residual glass. B-GC sample was prepared by 600 °C/20 h nucleation and 670 °C/20 h crystallization, and BZ-GC sample was prepared by 630 °C/20 h nucleation and 720 °C/20 h crystallization. Source data of Fig. 3a–c are provided as a Source data file.

Solid-state nuclear magnetic resonance (NMR) spectroscopy revealed that boron in both B and BZ glasses predominantly existed as [BO_3_] units with minor [BO_4_]^−^ contributions (Fig. 3a), consistent with alkali borosilicate glasses^25^. Given boron’s coordination preference and the structural constraints of Na_4_Ca_4_Si_6_O_18_ crystals, boron was excluded from the crystalline phase and enriched in residual glassy regions at grain boundaries. Crystallization induced minimal chemical shift changes for [BO_3_] and [BO_4_]^−^ units, but significantly enhanced [BO_4_]^−^ content relative to [BO_3_] in B-GC. This indicated substantial compositional difference in residual glassy phases between B-GC and BZ-GC. ^29^Si NMR spectra of B and BZ glasses exhibited mutual similarity (Fig. 3b), analogous to Na_2_O-2CaO-3SiO_2_ glass^26^. Spectral deconvolution using three Gaussian functions yielded components at −74.5 ppm (Q^1^), −80.5 ppm (Q^2^), and −89.8 ppm (Q^3^), corresponding to [SiO_4_] tetrahedra with one, two, and three bridging oxygens, respectively^26^. Q^2^ dominance aligned with glass compositions and prior reports^27,28^. Post-crystallization, ^29^Si NMR spectra shifted toward upfield with narrowed line widths (Fig. 3c). Constrained equal-linewidth deconvolution of crystallized samples yielded three components matching Na_4_Ca_4_Si_6_O_18_’s distinct silicon sites in puckered silicate rings^26^ (Supplementary Table 1). Compared to B-GC, BZ-GC showed reduced sidebands, greater chemical shift dispersion, and spectral asymmetry. These features could be fitted using three components rather than the single component observed in high-temperature Na_4_Ca_4_Si_6_O_18_—reflecting enhanced crystallinity and structural disorder due to Na^+^/Ca^2+^ site mixing and [ZrO_6_] substitution for [CaO_6_] units.

### Optical transparency evolution during crystallization

**Fig. 4:**
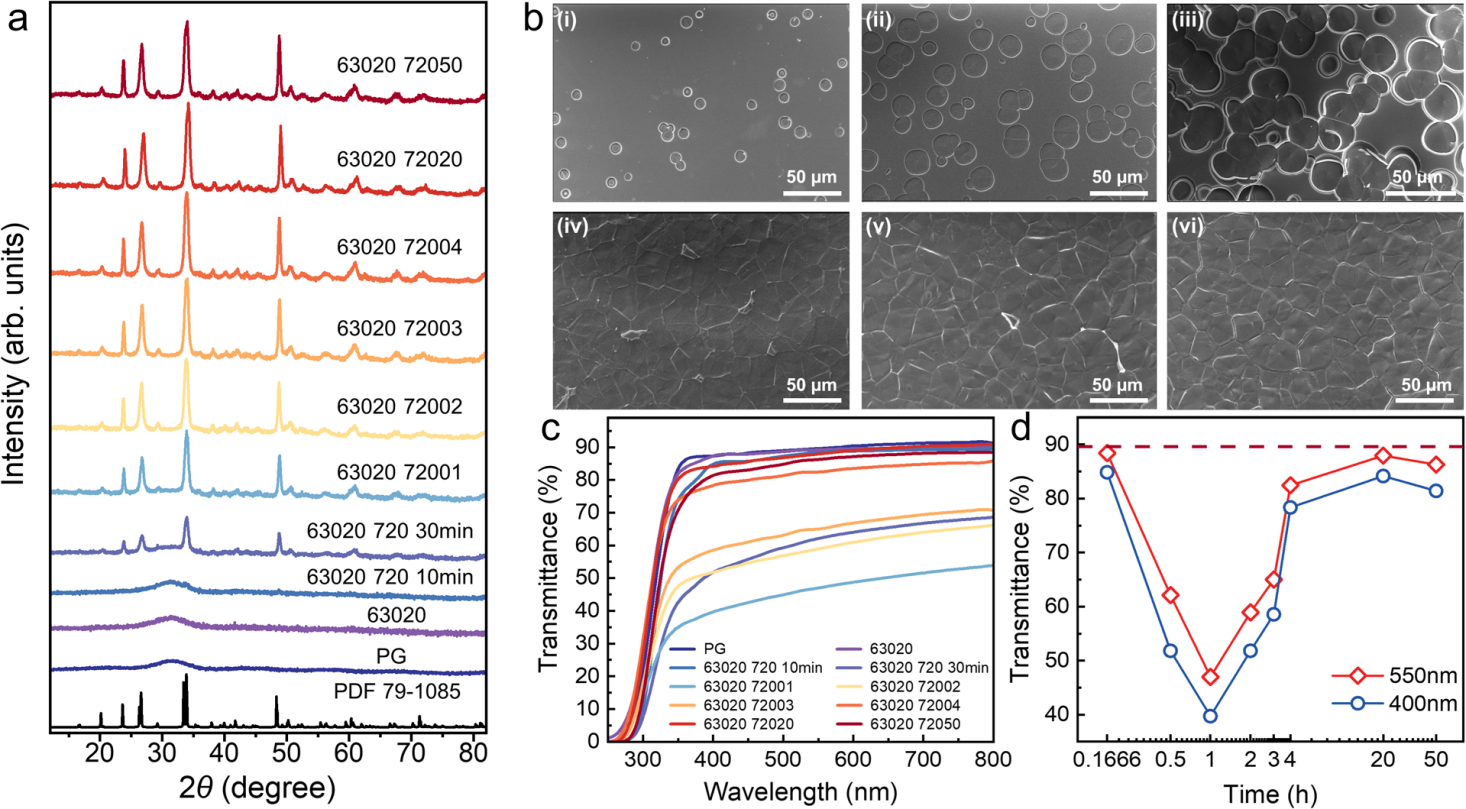
Crystallization-dependent structural and optical evolution during BZ glass crystallization. **a** XRD patterns showing phase evolution during heat treatment. PG denotes parent glass; other samples are labeled according to their heat treatment schedule. For example, 63020 represents 630 °C for 20 h; 63020 720 10 min indicates 630 °C/20 h followed by 720 °C/10 min; and 63020 72001 denotes 630 °C/20 h followed by 720 °C/1 h. **b** FE-SEM secondary electron micrographs illustrating microstructural development at i 10 min, ii 30 min, iii 1 h, iv 3 h, v 4 h, vi 50 h treatment durations. **c** Transmittance spectra (250–800 nm) demonstrating optical transparency evolution. The thickness of the specimens was 1 mm. **d** Quantitative transmittance evolution at 400 nm (blue) and 550 nm (red) as a function of second-step treatment time, with a dashed line indicating the precursor glass (PG) reference at 550 nm. Source data of Fig. 4a–d are provided as a Source data file.

The evolution of transparency in BZ glass was further investigated through a two-stage thermal treatment: nucleation at 630 °C for 20 h, followed by crystallization at 720 °C for varying durations (Fig. 4). Post-nucleation XRD analysis revealed only a broad amorphous halo (Fig. 4a), confirming the absence of crystalline phases. After 10 min of crystallization, weak diffraction peaks emerged superimposed on the amorphous background (Fig. 4a), with these peaks progressively intensifying and stabilizing upon extended crystallization (Fig. 4a), unequivocally confirming the precipitation of crystals closely matching the Na_4_Ca_4_Si_6_O_18_ crystals (PDF#79-1085).

Field-emission scanning electron microscope (FE-SEM) characterization documented the microstructural evolution (Fig. 4b): at 10-min crystallization, ~10 µm spherical grains dispersed within the glass matrix; prolonged treatment induced grain growth and impingement, transforming the microstructure from isolated crystalline spheres in glass to discrete glassy pockets within a crystalline continuum; beyond 20 h, nearly complete elimination of residual glassy phases yielded ~30 µm polyhedral grains with defined boundaries. This microstructural progression directly modulated optical transmittance (Fig. 4c, d, and Supplementary Fig. 6), exhibiting a characteristic trajectory: initial transmittance at 400 nm decreased from 87.3% (as-prepared/nucleated state) to 39.7% after 1-h crystallization, subsequently recovering to 84.1% following 20-h treatment. Throughout this evolution, long-wavelength transmittance consistently surpassed performance at 400 nm (Fig. 4c, d). Taking BZ-GC (Fig. 2d) as a representative example, both FE-SEM of its freshly fractured surface and confocal laser scanning microscopy (CLSM) of optically polished specimen confirmed a dense, pore-free microstructure (Supplementary Fig. 7). The CLSM analysis, with a detection resolution of roughly <100 nm^29^, scanned a three-dimensional volume of 100 × 100 × 20 μm^3^ and revealed no evidence of pores (Supplementary Fig. 7c), indicating that pore formation was effectively suppressed.

### Anisotropy engineering and phase stabilization

**Fig. 5:**
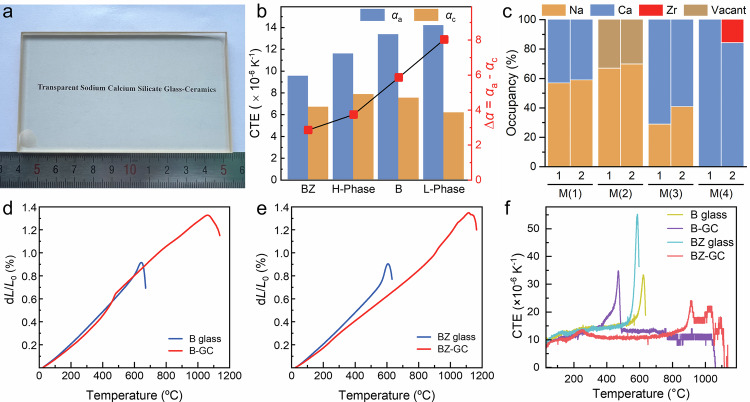
Scalable transparent BZ-GC with engineered thermal expansion anisotropy. **a** Macroscopic demonstration of optical-quality BZ-GC (11.3 × 6.5 × 0.6 cm^3^) exhibiting uniform light transmission. **b** Comparison of thermal expansion coefficients (CTE) along principal crystallographic axes and anisotropy parameter Δ*α* between BZ-GC, B-GC, and reference Na_4_Ca_4_Si_6_O_18_ polymorphs (high-temperature phase: H-phase; low-temperature phase: L-phase). **c** Structural refinement showing cation distribution at M(1)–M(4) sites in (1) standard reference Na_4_Ca_4_Si_6_O_18_ (PDF#79-1085) and (2) BZ-GC. Comparative thermal expansion behavior of: **d** B glass and B-GC, and **e** BZ glass and BZ-GC. **f** Derived CTE temperature dependence from dilatometric analyses. B-GC was prepared by 600 °C/20 h nucleation and 670 °C/20 h crystallization, and BZ-GC was prepared by 630 °C/20 h nucleation and 720 °C/20 h crystallization. Source data of Fig. 5a–f are provided as a Source data file.

The high transmittance of crack-free BZ-GC demonstrated reduced thermal expansion anisotropy, minimizing residual stress and stress-induced birefringence. This hypothesis of [ZrO_6_] polyhedra substituting [CaO_6_] polyhedra was confirmed through the successful fabrication of large-scale BZ-GC. Employing identical melt-forming and thermal treatment protocols, we produced transparent BZ-GC measuring 11.3 × 6.5 × 0.6 cm^3^ (Fig. 5a). To better demonstrate transparency, a photograph was also taken with the sample positioned ~1.5 cm away from the background (Supplementary Fig. 8). To quantitatively assess thermal expansion behavior, in situ temperature-dependent XRD patterns were acquired across 25–425 °C (Supplementary Fig. 9), with Rietveld refinement analysis determining lattice parameters (Supplementary Fig. 10). Linear regression analysis yielded thermal expansion coefficients of *α*_a_ = 9.54 × 10^−6^ K^−1^ and *α*_c_ = 6.68 × 10^−6^ K^−1^ (Fig. 5b), values notably lower than those characteristic of high-temperature Na_4_Ca_4_Si_6_O_18_. The derived anisotropy parameter $\Delta \alpha \,={\alpha }_{{\mbox{a}}}\,-{\alpha }_{{\mbox{c}}}$ = 2.86 × 10^−6^ K^−1^ demonstrated significant reduction compared with literature values for high-temperature (3.74 × 10^−6^ K^−1^) and low-temperature (8.03 × 10^−6^ K^−1^) polymorphs^16^, directly enabling the exceptional optical transmission. The derived anisotropy parameter Δ*α* (5.86 × 10^−6^ K^−1^) for crystals and the residual glass phases at grain boundaries in B-GC caused the low transmission.

Analysis of the thermal expansion characteristics suggested that six-coordinated Zr^4+^ ions preferentially substituted Ca^2+^, necessitating charge compensation through increased Na^+^ occupancy, resulting in the formation of Na^+^-enriched, Ca^2+^-deficient Na_4_Ca_4_Si_6_O_18_ crystals in BZ-GC. Rietveld refinement analysis suggested significant Na^+^ enhancement and approximately 15.6% Zr^4+^ substitution for Ca^2+^ at M(4) sites (Fig. 5c). The calculated cation distribution indicated that the crystalline phase in BZ-GC should be Na_4.506_Ca_3.435_Zr_0.156_Si_6_O_18_. This cationic redistribution reduced Na^+^ concentration in residual glassy phases of BZ-GC relative to B-GC, where the crystal phase should be Na_4_Ca_4_Si_6_O_18_, explaining the observed [BO_4_]^−^ enrichment in the latter system (Fig. 3b)^25^. Thermal dilatometry provided further validation: B-GC exhibited characteristic low-temperature polymorph transition at ~473 °C (Fig. 5d, f)^16^, while BZ-GC maintained high-temperature polymorph stability until ~910 °C (Fig. 5e, f)—a 437 °C elevation in transition temperature confirming that [ZrO_6_] substitution and non-stoichiometric Na/Ca ratios effectively stabilized the high-temperature phase. This stabilization yielded a reduced thermal expansion coefficient across the 25–780 °C range compared with B-GC containing the low-temperature polymorph.

### Potential applications of transparent SCS glass-ceramics

The transparent SCS glass-ceramics exhibit promise for multiple applications, including high-temperature structural materials and biomedical materials. Dilatometric measurements showed that BZ glasses softened at approximately 606 °C (Fig. 5e). In contrast, no softening was observed in BZ-GC below 900 °C. It also demonstrated a high phase transition temperature (~910 °C) and a significantly reduced thermal expansion coefficient across a wide temperature range (Fig. 5e, f). Notably, the thermal expansion coefficient of BZ-GC was comparable to that of commercial soda-lime glass (9.35 × 10^−6^ K^−1^, 0–300 °C)^30^. These enhanced thermal properties indicate high-temperature stability, making BZ-GC excellent candidate for high-temperature structural materials, such as fire-resistant windows.

**Fig. 6:**
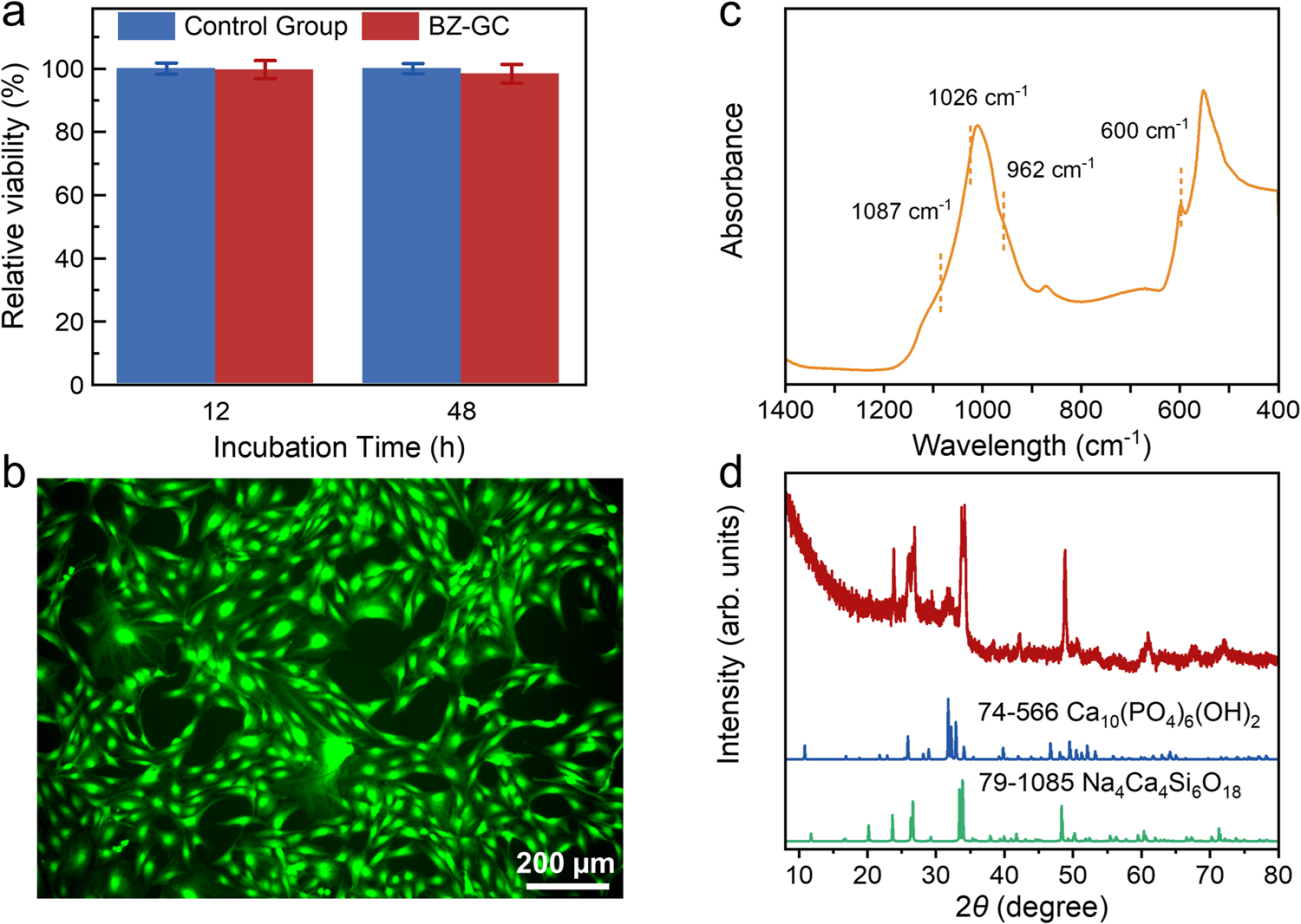
In vitro biocompatibility and bioactivity evaluation of BZ-GC. **a** Cell viability assessment via MTT assay showing comparable biocompatibility between control and BZ-GC groups at 12 and 48 h incubation periods (error bars represent standard deviation). **b** Fluorescent live/dead staining (calcein-AM/PI) of osteoblasts cultured on BZ-GC surfaces for 48 h, demonstrating ~100% viability (green: live cells; red: dead cells). **c** FTIR-ATR spectra and **d** XRD pattern of the BZ-GC surface after immersing in SBF for 7 days. BZ-GC was prepared by 630 °C/20 h nucleation and 720 °C/20 h crystallization. Source data of Fig. 6a–d are provided as a Source data file.

SCS glasses, particularly 45S5 Bioglass®^31^, are renowned for their biocompatibility and bioactivity. Crystallization further improves their mechanical properties, with Vickers hardness increasing from 6.2 ± 0.1 GPa (BZ glass) to 7.1 ± 0.1 GPa (BZ-GC), and three-point bending strength enhancing from 60.6 ± 4.61 to 114.4 ± 10.4 MPa. This trend extends to fracture toughness, which rises from 0.8 ± 0.3 MPa m^1/2^ (BZ glass) to 1.5 ± 0.2 MPa m^1/2^ (BZ-GC), and Young’s modulus, which increases from 88.8 ± 0.2 to 120.1 ± 0.5 GPa. These mechanical improvements, combined with high optical transmittance, significantly expand the biomedical application potential of BZ-GC. A comparative analysis of Vickers hardness and three-point bending strength among conventional transparent ceramics, glass-ceramics, and bioactive ceramics^32–39^ (Supplementary Fig. 11) further contextualizes this performance. While the BZ-GC does not reach the strength levels of conventional transparent ceramics, its Vickers hardness is higher than that of typical bioactive ceramics, highlighting distinct advantages for biomedical use. To further evaluate biocompatibility, we conducted toxicity assessments using 3-(4,5-dimethylthiazol-2-yl)-2,5-diphenyltetrazolium bromide (MTT) and calcein acetoxymethyl ester/propidium iodide (calcein-AM/PI) staining assays. The MTT assay revealed nearly 100% cell viability after both 12 and 48 h incubation periods compared with controls (Fig. 6a), demonstrating no toxic effects on cell proliferation. Confocal microscopy analysis of calcein-AM/PI stained samples showed no dead cells (Fig. 6b), corroborating the MTT results and confirming the excellent biocompatibility of BZ-GC. The bioactivity of the BZ-GC was evaluated by examining its ability to induce hydroxyapatite (HA) formation in simulated body fluid (SBF). HA is a key biomaterial known to form direct bonds with bone tissue^40^. The appearance of the specimen after immersion is illustrated in Supplementary Fig. 12. Attenuated total reflectance-Fourier transform infrared (ATR-FTIR) spectroscopy collected from the sample surface after 7 days of immersion (Fig. 6c) displayed characteristic absorption bands at 1087, 1026, 962, and 600 cm^−1^, which were attributed to phosphate (${{{{\rm{PO}}}}}_{4}^{3-}$) groups indicative of HA^40,41^. This finding was corroborated by XRD analysis (Fig. 6d), in which distinct diffraction peaks corresponding to crystalline HA were clearly observed. Furthermore, FE-SEM observation (Supplementary Fig. 13) revealed a dense and continuous layer of newly formed particles covering the surface of BZ-GC. EDS mapping confirmed the co-localization of Ca and P within this layer (Supplementary Fig. 13), providing unequivocal evidence of HA precipitation. This bioactive behavior is consistent with the previously reported properties of related crystalline phases, such as Na_2_Ca_2_Si_3_O_9_^42^, substantiating the potential of BZ-GC for biomedical applications. Hydrolytic stability tests further confirmed that crystallization significantly improves the material’s durability in aqueous environments. BZ-GC exhibited a mass loss of only ~5.1 g/m^2^ after 14 days in deionized water at 37 °C, compared to ~9.6 g/m^2^ for the BZ glass under the same conditions (Supplementary Fig. 14). These findings collectively demonstrate the significant potential of BZ-GC for biomedical applications, enabling non-invasive in situ monitoring of cell cultures and tissue engineering^43,44^.

In summary, we present a structural engineering approach that mitigates the thermal expansion anisotropy of crystals, leading to the preparation of highly crystalline SCS glass-ceramics with desirable microstructure and properties. Through targeted Zr^4+^ substitution at Ca^2+^ sites in the Na_4_Ca_4_Si_6_O_18_ crystal, we reduced thermal expansion anisotropy (Δ*α* = 2.86 × 10^−^^6^ K^−^^1^) while stabilizing the high-temperature polymorph, enabling large-sized transparent SCS glass-ceramics with homogeneous microstructure. Crucially, the methodology leverages existing industrial glass infrastructure to produce TGCs, demonstrating dual functionality through exceptional bioactivity and remarkable thermal stability across extended temperature ranges. This work expands the application range of low-cost soda-lime-silicate glass-ceramics and also provides a feasible strategy for the preparation of highly crystalline TGCs.

## Methods

### Materials and preparation

Based on previous research on SCS glass-ceramics^14,24^, precursor glasses with nominal composition (mol.%) of (1 − (*x* + *y*)/100)[49.27SiO_2_−34.48CaO-16.25Na_2_O]-*x*ZrO_2_-*y*B_2_O_3_ were designed through iterative optimization (*x* = 0, *y* = 2: B glass; *x* = 2, *y* = 2: BZ glass). To ensure the compositional accuracy, the as-annealed glasses were analyzed by inductively coupled plasma-optical emission spectrometry (ICP-OES, Prodigy 7, LEEMAN, USA). The measured compositions (in mol.%) were 48.57 SiO_2_-32.61 CaO-17.63 Na_2_O-1.19 B_2_O_3_ for the B glass and 47.49 SiO_2_-32.21 CaO-17.26 Na_2_O-1.69 ZrO_2_-1.35 B_2_O_3_ for the BZ glass. Both glasses show a slight enrichment in Na_2_O relative to the stoichiometric Na_2_O·2CaO·3SiO_2_ formulation, with Si/Ca molar ratios close to 1.5. This deliberate off-stoichiometry was adopted to stabilize the high-temperature polymorph and to tailor the glass-forming ability and crystallization, thereby enabling the fabrication of transparent and highly crystalline glass-ceramics. Commercial oxide powders (SiO_2_, CaCO_3_, Na_2_CO_3_, B_2_O_3_, ZrO_2_; 99.9% purity, Sinopharm Chemical Reagent Co. Ltd.) were homogenized in ~120 g batches, melted in platinum crucibles at 1550 °C for 2.5 h (ambient atmosphere), and cast into brass molds preheated at 500 °C to minimize thermal shock. Cast glasses were annealed at 530 °C for 5 h in a muffle furnace to relieve thermal stresses. Annealed B and BZ glasses were transparent. These glasses were sectioned into 1 cm × 1 cm × 2 mm specimens for thermal processing. Controlled crystallization was guided by differential scanning calorimetry (DSC, Netzsch STA 449F3; 10 °C/min heating rate, N_2_ atmosphere). Nucleation occurred at slightly above the glass transition temperature (*T*_g_) for 20 h, followed by crystal growth at temperatures close to the exothermic peak for variable durations. Post-treatment specimens were polished to ~1.5 mm thickness. Large-size transparent SCS glass-ceramics were prepared similarly using ~300 g batches.

### Microstructure characterization

XRD analysis was performed using a Bruker D8 Advance diffractometer with Cu Kα radiation, recording patterns across the 10–120° 2*θ* range at a 0.02° scanning step. Specimens were ground into fine powders prior to measurement. Rietveld refinement of diffraction data was conducted using the general structure analysis system (GSAS) program^45^. For quantitative Rietveld refinement, 30 wt% rhombohedral α-Al_2_O_3_ was added to the powder samples as an internal standard and thoroughly mixed by a mortar. In situ high-temperature XRD studies spanned 25–425 °C with measurements taken at 100 °C intervals, employing a heating rate of 10 °C/min and 60-s isothermal holds at each temperature for pattern acquisition. Lattice parameters were determined through GSAS peak fitting. Microstructural evaluation employed a Zeiss Ultra Plus FE-SEM for morphology characterization. HR-TEM was performed on a Talos-F200S microscope (ThermoFisher Scientific, USA). The TEM specimen was prepared by dimple grinding followed by Ar^+^ ion milling under liquid-nitrogen cooling to minimize damage and was subsequently coated with carbon on both sides to mitigate charging effects during observation. LA-ICP-MS was employed for multi-elemental mapping. The analysis was performed using an Iridia laser system equipped with a 193 nm ArF excimer laser, coupled via an aerosol rapid introduction system to a NexION 300D ICP-MS (PerkinElmer, Norwalk, USA). High-purity helium served as the carrier gas to transport ablated aerosols from the chamber to the ICP-MS. The laser spot size and line-scan spacing were both set to 1 µm. Operating conditions were optimized to a single-pulse response of ~10 ms and a laser repetition rate of 100 Hz. Data integration and image processing were carried out using Iolite software (v3.6) running under Igor Pro 7 (WaveMetrics, USA). Time of flight secondary ion mass spectrometry (TOF-SIMS, nanoTOF 3, CoreTech, China) with a Bi ion source was also used for positive ion TOF-SIMS imaging. Solid-state NMR spectroscopy was conducted on a Bruker AVANCE III 600 WB spectrometer operating at 14.09 T for ^11^B and a 400 WB spectrometer operating at 9.4 T for ^29^Si. ^11^B and ^29^Si NMR spectra were acquired at Larmor frequencies of 192.58 and 79.49 MHz, respectively. The ^11^B measurements utilized a Bruker CPMAS BL4 WVT probe with MgO stator (eliminating background signals) coupled with 4 mm zirconia rotors spinning at 12 kHz. ^29^Si NMR employed magic-angle spinning (MAS) at 5 kHz using a 7 mm widebody CP/MAS probe with corresponding zirconia rotors. All measurements were performed at ambient temperature unless otherwise specified. To evaluate the presence of pores, BZ-GC were examined using complementary microscopic techniques. FE-SEM was performed on freshly fractured surfaces. In addition, confocal laser scanning microscopy (CLSM, Zeiss LSM880, Germany) was employed on optically polished surfaces to detect sub-surface pores, which appear as bright spots against a dark background under the CLSM imaging mode. The spatial resolution of the CLSM system was estimated to be roughly <100 nm. For the CLSM analysis, a series of optical slices (each ~100 × 100 µm^2^) were acquired at a depth interval of 400 nm. Multiple slices (acquisition time: 30 s per slice) were then reconstructed to generate a three-dimensional volume of 100 × 100 × 20 µm^3^, enabling a volumetric inspection for pores within the detection limit of the instrument.

### Property measurements

Optical transmission properties were characterized using a PerkinElmer Lambda 750 s UV–vis-NIR spectrometer, with spectra acquired at room temperature for both as-prepared glasses and crystallized specimens. Mechanical properties were evaluated through microhardness testing on polished samples using a QNESS Q10A+ Vickers hardness tester. Five indentations spaced uniformly across each specimen were performed under a 1.96 N load to determine Vickers hardness (HV) values. Thermal expansion behavior was investigated via dilatometric analysis on a Netzsch DIL 402 C thermal dilatometer, with measurements conducted from 25 to 1200 °C at a heating rate of 5 °C/min. Specimens for dilatometry were fabricated as 25 × 4 × 4 mm^3^ rectangular bars with all surfaces mechanically polished to optical quality. The bending strength was measured using a three-point bending method on a universal testing machine (Instron 5967, USA). The support span was set to 30 mm, with a loading speed of 9.8 ± 0.1 N/s. The sample dimensions were 40 × 4 × 3 mm^3^. Fracture toughness was measured using the single-edge precracked beam method in accordance with ASTM C1421-99 (see Supplementary Fig. 15 for details). Five bar specimens with dimensions of 40 × 4 × 3 mm^3^ were tested using a universal testing machine (5967, Instron, USA). Young’s modulus was determined by the impulse excitation technique (ASTM E1876-22) using an IET-01 instrument (Zhuosheng, China) on rectangular specimens (55.8 × 14.8 × 2.6 mm^3^). Hydrolytic stability was evaluated by immersing polished samples (15 × 10 × 2 mm^3^) in 50 mL of deionized water with a sealed polytetrafluoroethylene (PTFE) reactor at 37 °C. Prior to immersion, the samples were cleaned, and their surface area and initial mass were recorded. After 7 and 14 days, the samples were removed, dried thoroughly, and reweighed to determine the mass loss per unit surface area.

### In vitro cytotoxicity and bioactivity assessment

Mouse embryonic osteoblast cells (MC3T3-E1) were employed for in vitro toxicity evaluation. Cells were maintained in culture medium supplemented with 10% fetal bovine serum under humidified conditions at 37 °C with 5% CO_2_. Following three sequential passages, cell proliferation was quantified at designated intervals prior to experimentation. Cytotoxicity assessment of the BZ-GC sample was conducted via MTT assay, with untreated cells serving as controls. This colorimetric method utilizes 3-(4,5-dimethylthiazol-2-yl)-2,5-diphenyltetrazolium bromide (MTT), where mitochondrial succinate dehydrogenase in viable cells reduces the yellow tetrazolium salt to purple formazan crystals, providing a quantitative measure of cellular viability. Experimental procedures included: seeding 10,000 cells per well in 48-well plates, followed by incubation at 37 °C/5% CO_2_. Sterilized samples were then introduced to designated wells. Triplicate assays were performed with viability measurements at 12 and 48 h timepoints. At each endpoint, 500 µL MTT solution (0.5 mg/mL in media) was administered per well for 4 h. Resultant formazan crystals were solubilized with 300 µL dimethyl sulfoxide (DMSO), and absorbance was quantified at 570 nm with 630 nm reference using a BioTek microplate reader (Vinooski, VT, USA). Complementary fluorescence imaging was performed using confocal laser microscopy. After 48 h exposure to BZ-GC, cells were stained with calcein-AM/PI dual fluorescence probe. Calcein-AM (488 nm excitation) identified viable cells through green fluorescence, while propidium iodide (PI) indicated dead cells via red fluorescence emission.

The in vitro bioactivity was assessed by immersing polished BZ-GC in SBF at 37 °C for 7 days. The SBF was prepared with an ionic composition closely matching that of human blood plasma^46^. After immersion, the samples were thoroughly rinsed with deionized water and acetone. Surface precipitates were subsequently analyzed using Fourier-transform infrared (FTIR) spectroscopy in ATR mode (iN10-iS50, ThermoFisher Scientific, USA), XRD, and FE-SEM coupled with EDS.

## Supplementary information

Supplementary Information

Transparent Peer Review File

## Source data

Source Data

## Data Availability

Source data are provided with this paper. Additional data generated during this study are available from the authors upon request.

## References

[1] Liu, X., Zhou, J., Zhou, S., Yue, Y. & Qiu, J. Transparent glass-ceramics functionalized by dispersed crystals. *Prog. Mater. Sci.* **97**, 38–96 (2018).

[2] Rüssel, C. & Wisniewski, W. Glass-ceramic engineering: tailoring the microstructure and properties. *Prog. Mater. Sci.* **152**, 101437 (2025).

[3] Venkateswaran, C., Sreemoolanadhan, H. & Vaish, R. Lithium aluminosilicate (LAS) glass-ceramics: a review of recent progress. *Int. Mater. Rev.* **67**, 620–657 (2022).

[4] Allix, M. et al. Highly transparent BaAl_4_O_7_ polycrystalline ceramic obtained by full crystallization from glass. *Adv. Mater.* **24**, 5570–5575 (2012). 22899502 10.1002/adma.201202282

[5] Wang, D. et al. Congruent glass composite scintillator for efficient high-energy ray detection. *Adv. Mater.* **37**, 2412661 (2025). 10.1002/adma.202412661 39501993

[6] You, F. et al. Amorphous engineering of transparent high-crystallinity luminescent nano-glass-ceramics for advanced photonic applications. *Adv. Mater.* **38**, e20325 (2026). 41441848 10.1002/adma.202520325

[7] Yang, Y. et al. Advances in glass-ceramics raw material selection-performance modulation-application expression: a review. *Mater. Today* **89**, 270–292 (2025).

[8] Ueno, S., Tanabe, Y. & Nakagawa, Z. E. Preparation of cordierite/mullite composites by solidification process. *J. Ceram. Soc. Jpn.* **111**, 528–532 (2003).

[9] Fokin, V. M. et al. Mutant crystals in Na_2_O·2CaO·3SiO_2_ glasses. *J. Non Cryst. Solids* **331**, 240–253 (2003).

[10] Fokin, V. M., Zanotto, E. D., Yuritsyn, N. S. & Schmelzer, J. W. P. Homogeneous crystal nucleation in silicate glasses: a 40 years perspective. *J. Non Cryst. Solids* **352**, 2681–2714 (2006).

[11] Gonzalez-Oliver, C. J. R. & James, P. F. Crystal nucleation and growth in a Na_2_O·2CaO·3SiO_2_ glass. *J. Non Cryst. Solids* **38**, 699–704 (1980).

[12] Macena, G. S., Abyzov, A. S., Fokin, V. M., Zanotto, E. D. & Ferreira, E. B. Off-stoichiometry effects on crystal nucleation and growth kinetics in soda-lime-silicate glasses. The combeite (Na_2_O·2CaO·3SiO_2_) - devitrite (Na_2_O·3CaO·6SiO_2_) joint. *Acta Mater.* **196**, 191–199 (2020).

[13] Segnit, E. R. Further data on the system Na_2_O-CaO-SiO_2_. *Am. J. Sci.* **251**, 586–601 (1953).

[14] Berthier, T., Fokin, V. M. & Zanotto, E. D. New large grain, highly crystalline, transparent glass-ceramics. *J. Non Cryst. Solids* **354**, 1721–1730 (2008).

[15] Peterson, T. D. Peralkaline nephelinites. I. Comparative petrology of Shombole and Oldoinyo L’engai, East Africa. *Contrib. Mineral. Petrol.* **101**, 458–478 (1989).

[16] Ohsato, H., Takéuchi, Y. & Maki, I. Structural study of the phase transition of Na_4_Ca_4_ [Si_6_O_18_]. *Acta Crystallogr. B* **46**, 125–131 (1990).

[17] Soboleva, E. N., Yuritsyn, N. S. & Ugolkov, V. L. Kinetics of crystal nucleation of Na_2_O·2CaO·3SiO_2_-based solid solutions in glasses of the Na_2_SiO_3_-CaSiO_3_ pseudobinary join. *Glass Phys. Chem.* **30**, 481–486 (2004).

[18] Mastelaro, V. R. & Zanotto, E. D. Residual stresses in a soda-lime-silica glass-ceramic. *J. Non Cryst. Solids* **194**, 297–304 (1996).

[19] Pekov, I. V. et al. Crystal chemistry and nomenclature of the lovozerite group. *Eur. J. Mineral.* **21**, 1061–1071 (2009).

[20] Yamnova, N. A., Egorov-Tismenko, Y. K. & Pekov, I. V. Refined crystal structure of lovozerite Na_2_CaZr[Si_6_O_12_(OH,O)_6_]·H_2_O. *Crystallogr. Rep.* **46**, 937–941 (2001).

[21] Chernitsova, N. M., Pudovkina, Z. V., Voronkov, A. A., Kapustin, Y. L. & Pyatenko, Y. A. On the new crystallochemical lovozerite family. *Zapiski VMO* **104**, 18–27 (1975).

[22] Fan, D. et al. Compressibility and equation of state of beryl (Be_3_Al_2_Si_6_O_18_) by using a diamond anvil cell and in situ synchrotron X-ray diffraction. *Phys. Chem. Miner.* **42**, 529–539 (2015).

[23] Ikawa, H. et al. Crystal structure and mechanism of thermal expansion of high cordierite and its solid solutions. *J. Am. Ceram. Soc.* **69**, 492–498 (1986).

[24] Liao, W., Guo, Y., Lu, Y. & Liu, C. Crystallization of sodium calcium silicate glass for highly transparent glass-ceramics. *J. Eur. Ceram. Soc.* **44**, 3218–3225 (2024).

[25] Du, L.-S. & Stebbins, J. F. Solid-state NMR study of metastable immiscibility in alkali borosilicate glasses. *J. Non Cryst. Solids* **315**, 239–255 (2003).

[26] Bradtmüller, H., Villas-Boas, M. C., Zanotto, E. D. & Eckert, H. Structural aspects of the glass-to-crystal transition in sodium-calcium silicate glasses. *J. Non Cryst. Solids* **535**, 119844 (2020).

[27] Schneider, J., Mastelaro, V. R., Panepucci, H. & Zanotto, E. D. ^29^Si MAS-NMR studies of Q^n^ structural units in metasilicate glasses and their nucleating ability. *J. Non Cryst. Solids* **273**, 8–18 (2000).

[28] Schneider, J. et al. Q^n^ distribution in stoichiometric silicate glasses: thermodynamic calculations and ^29^Si high resolution NMR measurements. *J. Non Cryst. Solids* **325**, 164–178 (2003).

[29] Wagner, A., Ratzker, B., Kalabukhov, S., Sokol, M. & Frage, N. Residual porosity and optical properties of spark plasma sintered transparent polycrystalline cerium-doped YAG. *J. Eur. Ceram. Soc.* **39**, 1436–1442 (2019).

[30] Varshneya, A. K. & Mauro, J. C. *Fundamentals of Inorganic Glasses* (Elsevier Inc., 2019).

[31] Chen, Q. Z., Thompson, I. D. & Boccaccini, A. R. 45S5 Bioglass®-derived glass-ceramic scaffolds for bone tissue engineering. *Biomaterials* **27**, 2414–2425 (2006). 16336997 10.1016/j.biomaterials.2005.11.025

[32] Mu, Y. et al. Ion-exchange induced mechanical properties enhancement and microstructural modifications of transparent lithium aluminosilicate glass-ceramics. *Ceram. Int.* **50**, 14878–14883 (2024).

[33] Zhou, Z. et al. Influences of Al_2_O_3_ content on crystallization and physical properties of LAS glass-ceramics prepared from spodumene. *J. Non Cryst. Solids* **576**, 121256 (2022).

[34] Hao, X. et al. Effect of replacement of B_2_O_3_ by ZnO on preparation and properties of transparent cordierite-based glass-ceramics. *J. Non Cryst. Solids* **432**, 265–270 (2016).

[35] Huang, J. et al. Transparent MgO-Al_2_O_3_-SiO_2_ glass-ceramics prepared with ZrO_2_ and SnO_2_ as nucleating agents. *J. Non Cryst. Solids* **588**, 121585 (2022).

[36] Chen, Y. et al. 8 mol% Y_2_O_3_-stabilized zirconia (8YSZ) transparent ceramics with high optical and mechanical performance through YF_3_ sintering additive. *J. Eur. Ceram. Soc.* **45**, 117621 (2025).

[37] Ma, B. et al. Hot isostatic pressing of MgAlON transparent ceramic from carbothermal powder. *Ceram. Int.* **44**, 4512–4515 (2018).

[38] Liu, X., Wang, H., Tu, B., Wang, W. & Fu, Z. Highly transparent Mg_0.27_Al_2.58_O_3.73_N_0.27_ ceramic prepared by pressureless sintering. *J. Am. Ceram. Soc.* **97**, 63–66 (2014).

[39] Boilet, L. et al. Processing and properties of transparent hydroxyapatite and β tricalcium phosphate obtained by HIP process. *Ceram. Int.* **39**, 283–288 (2013).

[40] Rapacz-Kmita, A., Paluszkiewicz, C., Ślósarczyk, A. & Paszkiewicz, Z. FTIR and XRD investigations on the thermal stability of hydroxyapatite during hot pressing and pressureless sintering processes. *J. Mol. Struct.* **744-747**, 653–656 (2005).

[41] Hossain, M. S. & Ahmed, S. FTIR spectrum analysis to predict the crystalline and amorphous phases of hydroxyapatite: a comparison of vibrational motion to reflection. *RSC Adv.* **13**, 14625–14630 (2023). 37197675 10.1039/d3ra02580b PMC10183800

[42] Du, R. & Chang, J. Preparation and characterization of bioactive sol-gel-derived Na_2_Ca_2_Si_3_O_9_. *J. Mater. Sci. Mater. Med.* **15**, 1285–1289 (2004). 15747180 10.1007/s10856-004-5736-2

[43] Kotobuki, N. et al. Observation of osteogenic differentiation cascade of living mesenchymal stem cells on transparent hydroxyapatite ceramics. *Biomaterials* **26**, 779–785 (2005). 15350783 10.1016/j.biomaterials.2004.03.020

[44] John, A. et al. In vitro investigations of bone remodeling on a transparent hydroxyapatite ceramic. *Biomed. Mater.* **4**, 015007 (2009). 19020346 10.1088/1748-6041/4/1/015007

[45] Toby, B. H. EXPGUI, a graphical user interface for GSAS. *J. Appl. Cryst.* **34**, 210–213 (2001).

[46] Kokubo, T. & Takadama, H. How useful is SBF in predicting in vivo bone bioactivity? *Biomaterials* **27**, 2907–2915 (2006). 16448693 10.1016/j.biomaterials.2006.01.017

