# Supplementary material for: Scalable fabrication of transparent sodium calcium silicate glass-ceramics by structural engineering overcoming anisotropy barriers
Source: Nat Commun. 2026 Jul 10;17:8531. doi: 10.1038/s41467-026-75474-y (PMC13482340; doi:10.1038/s41467-026-75474-y)
Supplement: Supplementary file 1 [file 41467_2026_75474_MOESM1_ESM.pdf]

Supplementary Information

Scalable fabrication of transparent sodium calcium silicate glass-ceramics by structural engineering overcoming anisotropy barriers

Weifan Liao[1,2], Yunlan Guo[1,2], Kaiwen Hu[2,3], Jong Heo[2,3], Xiujian Zhao[2,3], Jian Ruan[1,2], Chao Liu[1,2,*]

[1]State Key Laboratory of Advanced Glass Materials, Wuhan 430070, China

[2]Silicate Materials Engineering Research Center, Wuhan University of Technology, Wuhan 430070, China

[3]State Key Laboratory of Silicate Materials for Architectures, Wuhan University of Technology, Wuhan 430070, China

*Corresponding author. E-mail: hite@whut.edu.cn

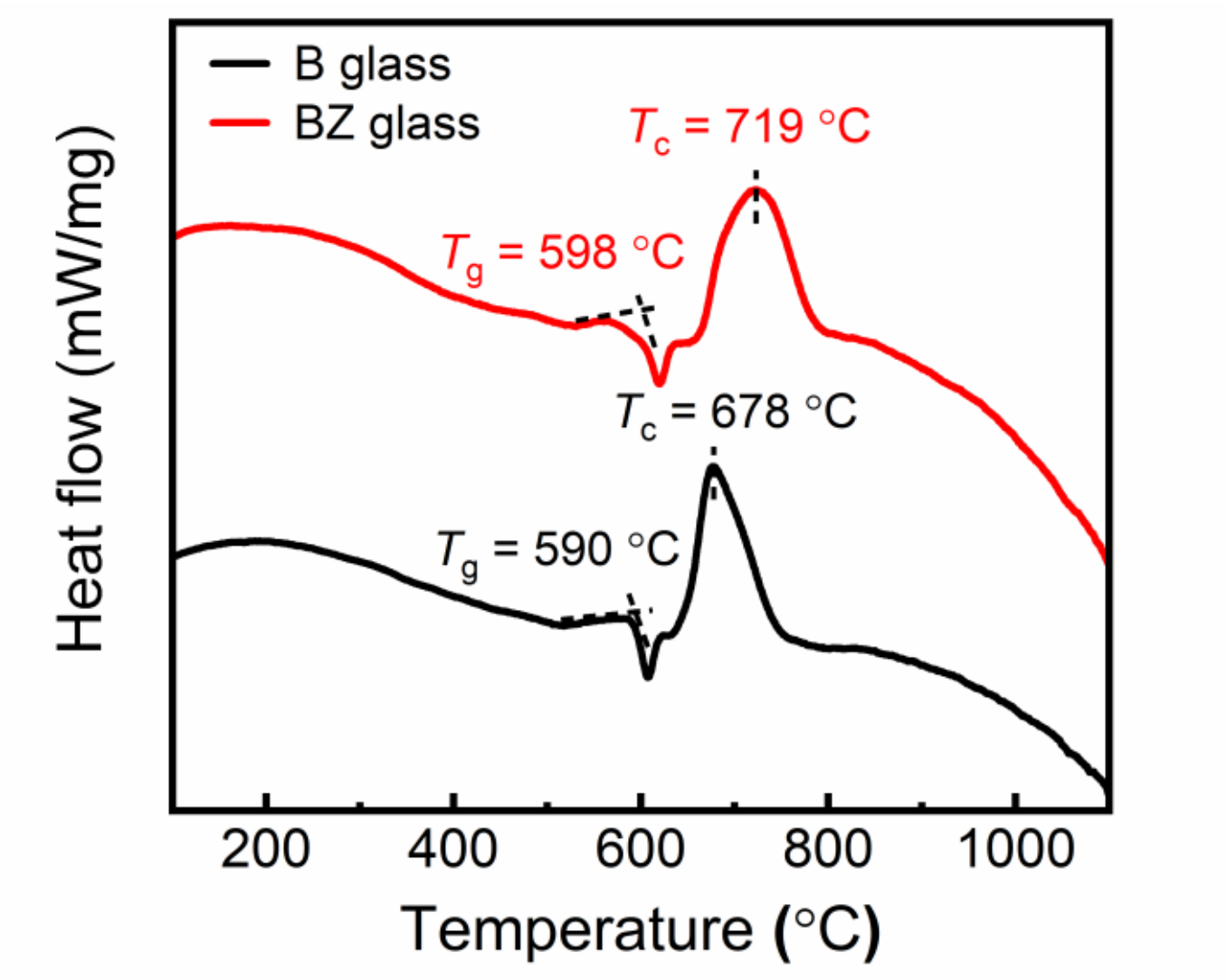

Supplementary Fig. 1. Thermal analysis of B and BZ precursor glasses by DSC. T_g represents glass transition temperature, and T_c represents the peak temperature of crystallization. Source data are provided as a Source Data file.

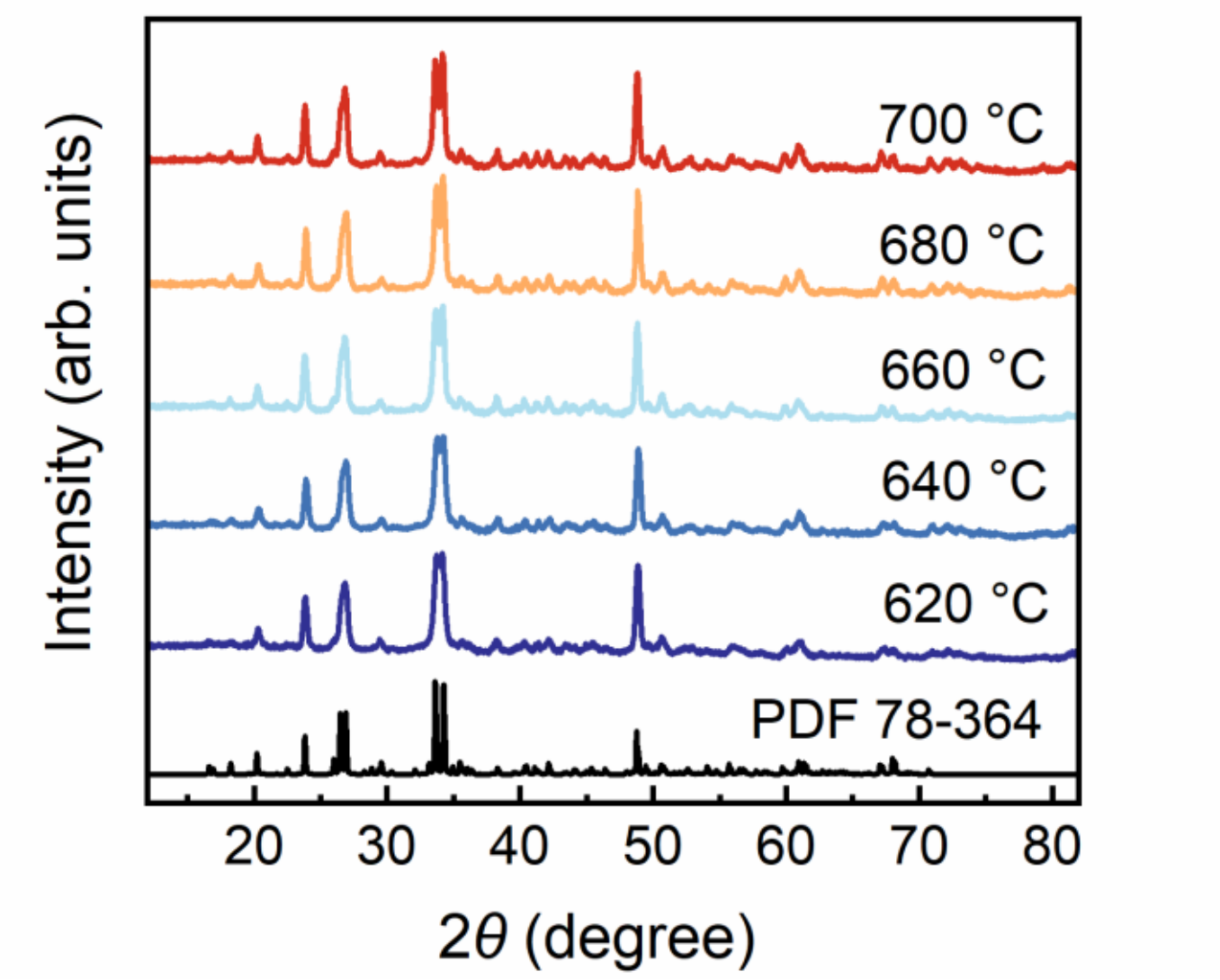

Supplementary Fig. 2. XRD patterns of B glasses crystallized at 620-700 °C for 20 h crystallization following 600 °C/20 h nucleation, with reference $Na_4Ca_4Si_6O_{18}$ low-temperature phase (PDF#78-364) shown for comparison. Source data are provided as a Source Data file.

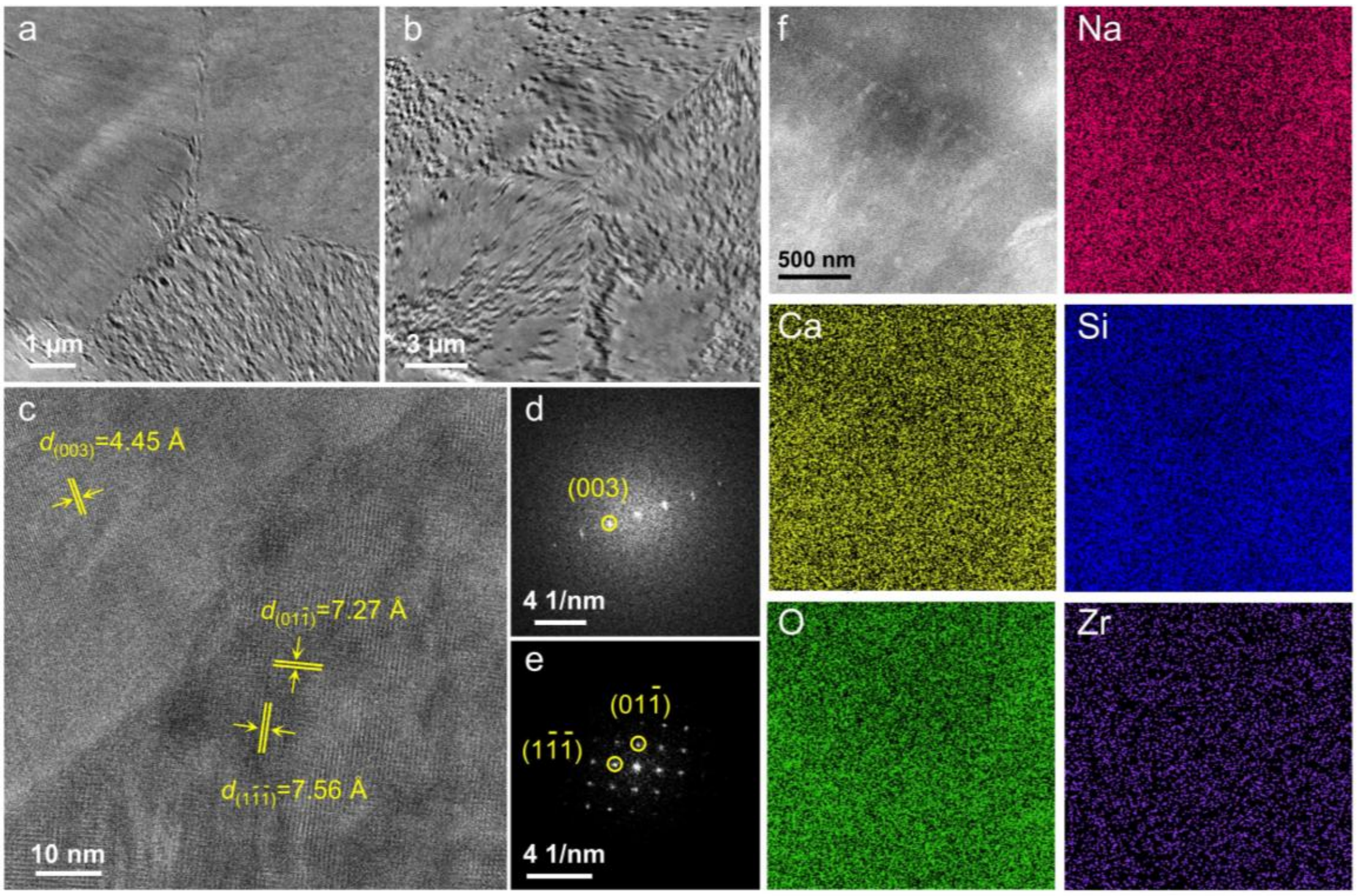

Supplementary Fig. 3. The microstructure of BZ-GC. a, **b** TEM image and (**c**) HR-TEM micrograph on the grain boundaries. **d**, **e** FFT patterns converted from the left-top and bottom-right region in (**c**), respectively. The interplanar distances shown in (**c**) are comparable to those of $Na_4Ca_4Si_6O_{18}$ crystal (PDF#79-1085) with $d_{(003)}$ = 4.40 Å, $d_{(01\bar{1})}$ = 7.52 Å, $d_{(1\bar{1}\bar{1})}$ = 7.52 Å. **f** Elemental mapping of Na, Ca, Si, O, and Zr on the grain boundary.

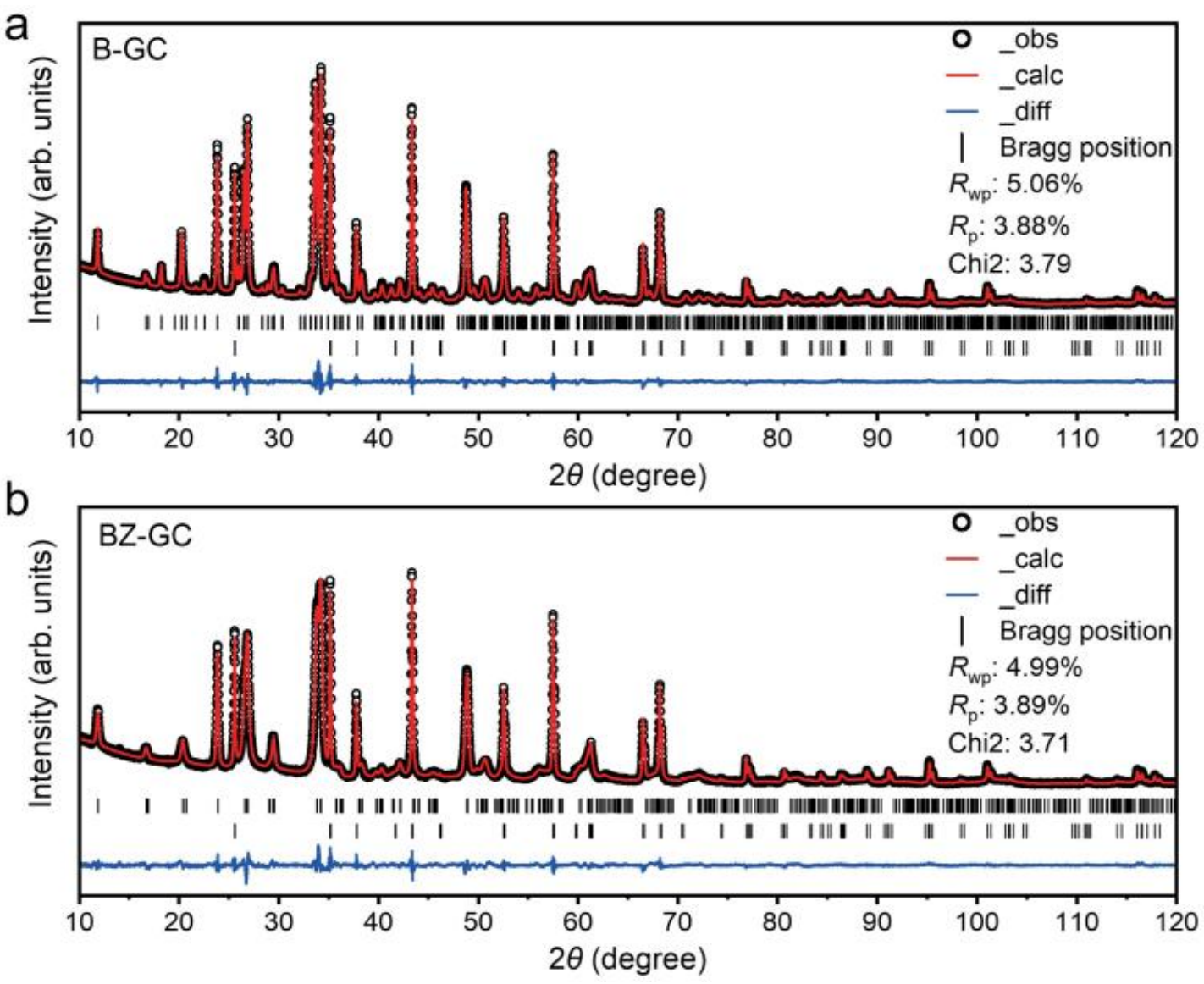

Supplementary Fig. 4. Rietveld refinement profiles of XRD patterns recorded from (**a**) B-GC and (**b**) BZ-GC with 30 wt% rhombohedral α-Al_2O_3 powder as an internal standard. Source data are provided as a Source Data file.

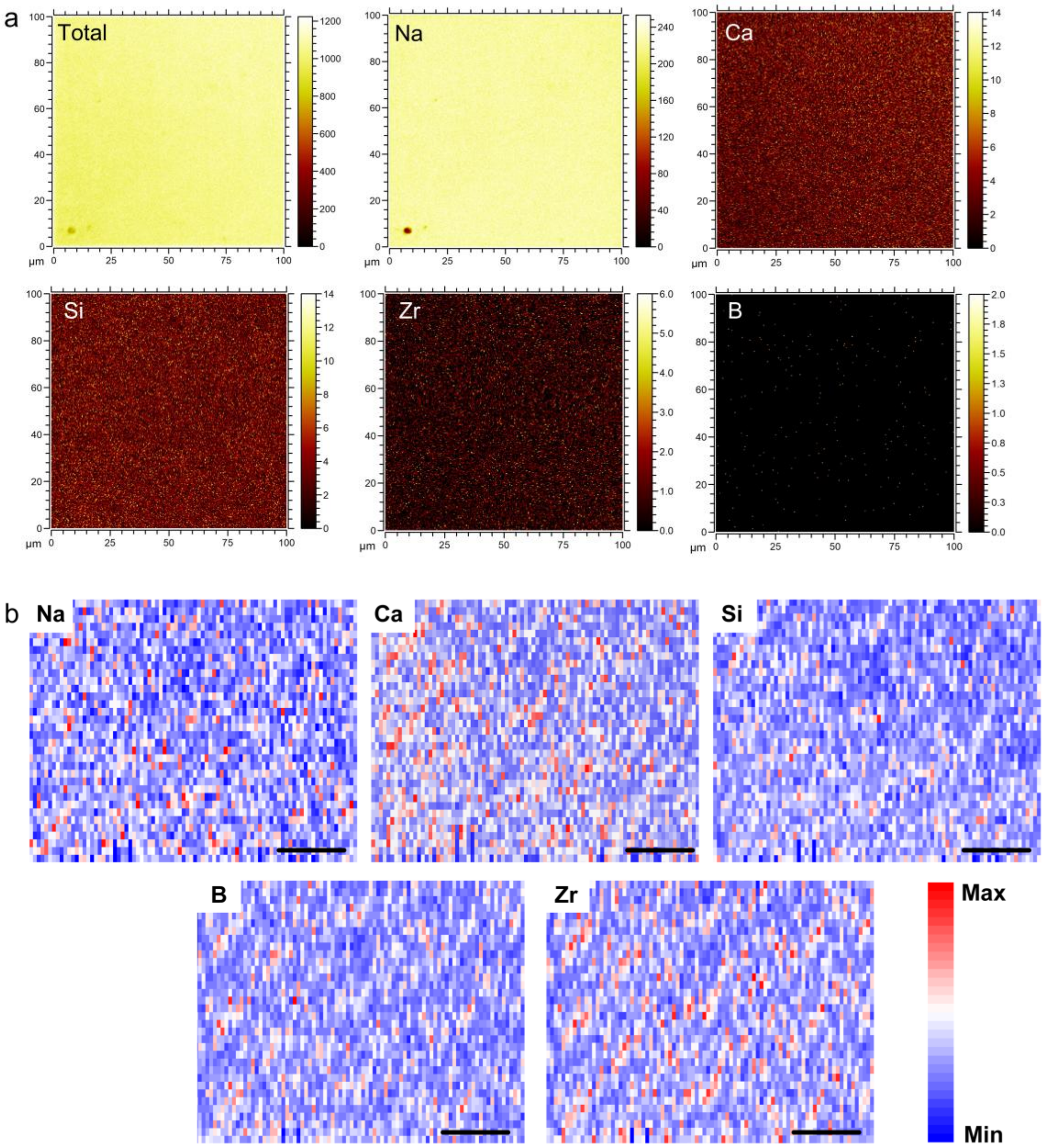

Supplementary Fig. 5. Elemental distribution in BZ-GC. a Positive ion TOF-SIMS maps of Na, Ca, Si, Zr, and B. Na, Ca, and Si distribute nearly homogeneously, whereas Zr and B show visibly inhomogeneous distributions (frame size: 100 μm). **b** LA-ICP-MS maps of Na, Ca, Si, B, and Zr in BZ-GC specimen (scale bar: 20 μm). The LA-ICP-MS results highlight the segregation of B and Zr along grain boundaries.

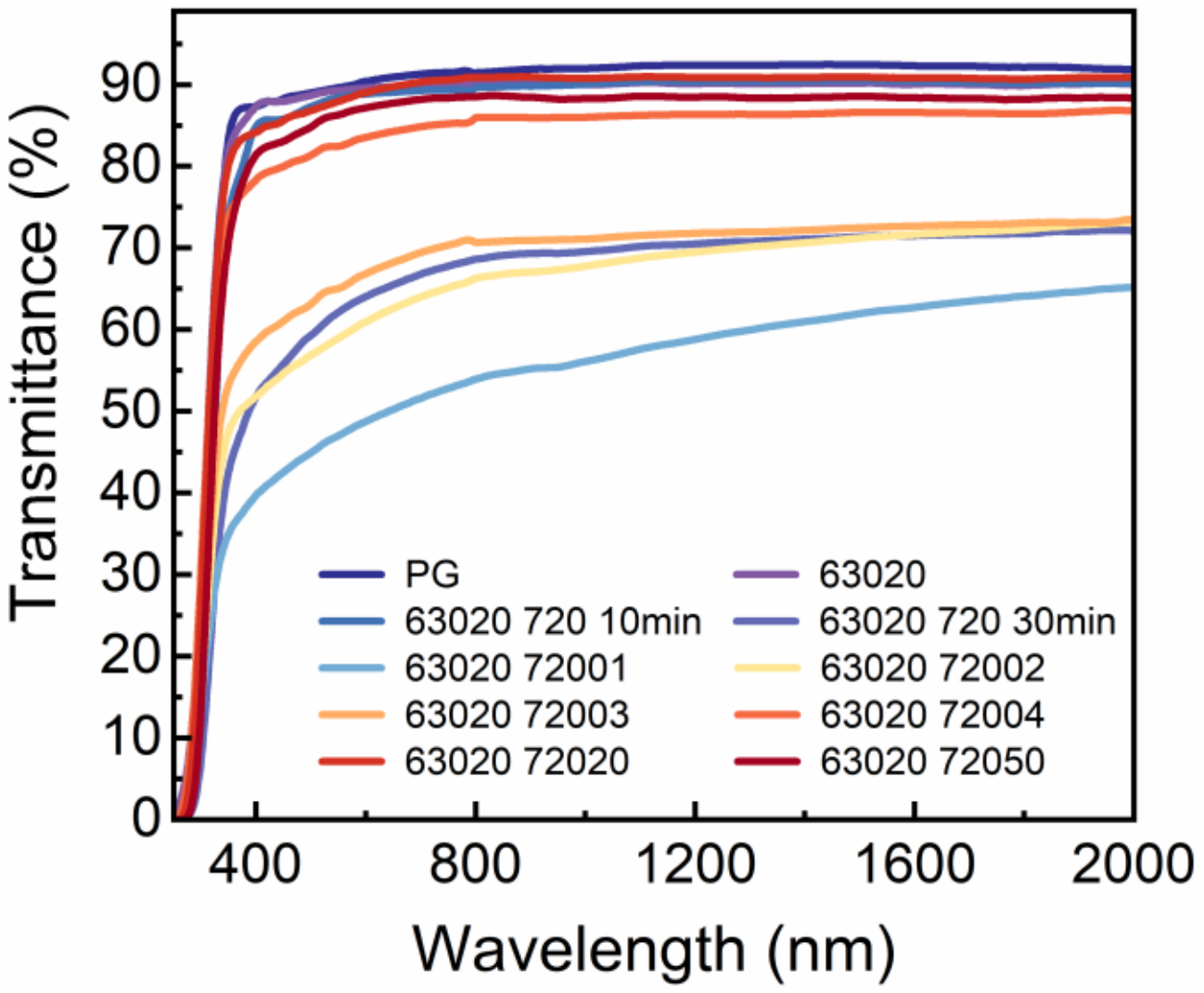

Supplementary Fig. 6. **Full-range optical transmittance.** UV-Vis-NIR transmission spectra (250-2000 nm) of the BZ precursor glass and the BZ samples subjected to various heat treatments. All samples were approximately 1 mm in thickness. Source data are provided as a Source Data file.

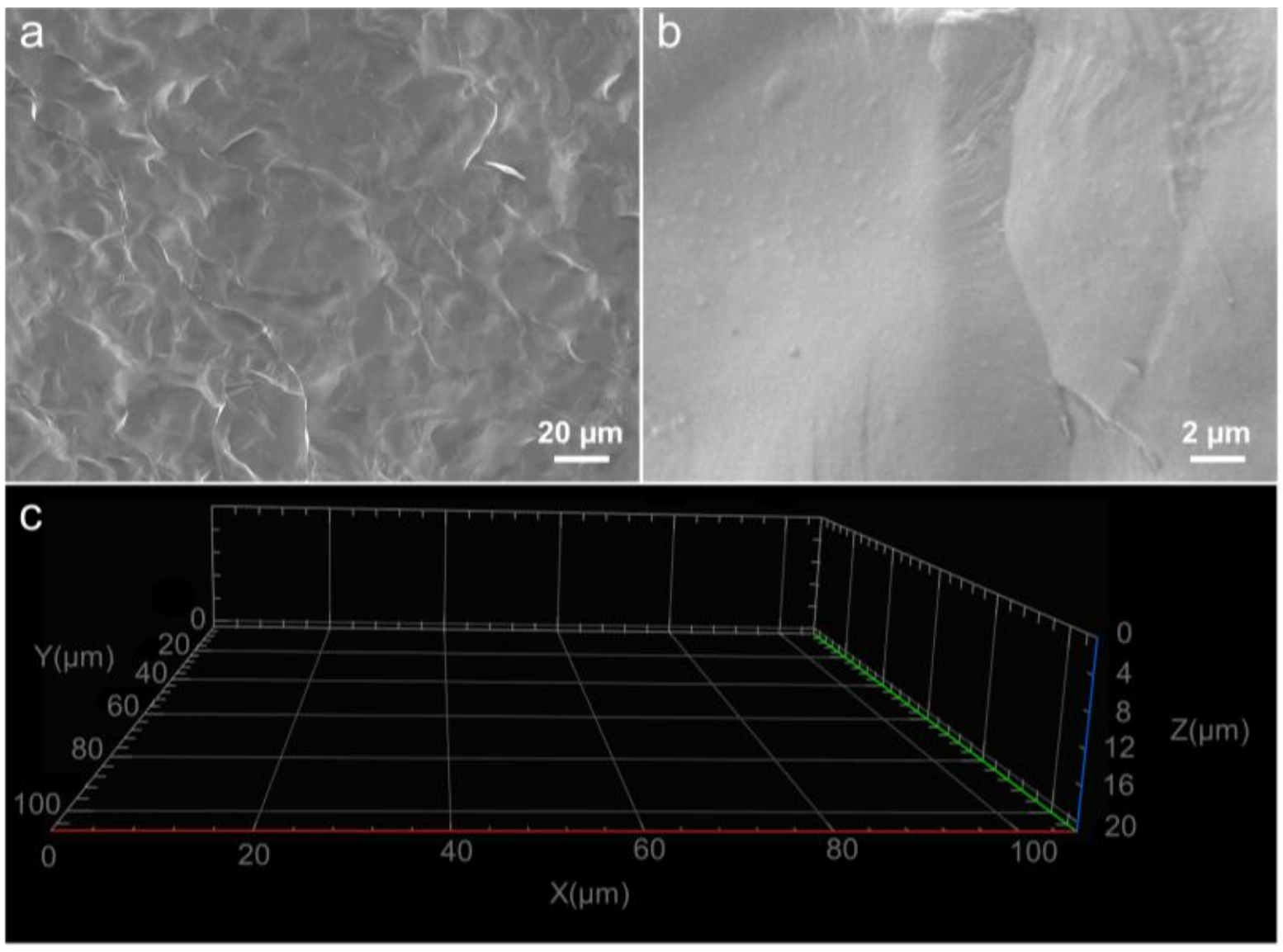

Supplementary Fig. 7. Microstructure and pore analysis of the BZ-GC. a, **b** SEM images (SE) of the fracture surface. The small round protrusions visible in (**b**) are induced artifacts that grow with prolonged irradiation time. **c** Three-dimensional confocal laser scanning microscopy (CLSM) reconstruction of a volume of 100×100×20 μm^3. No pores were detected within the instrument resolution of approximately <100 nm.

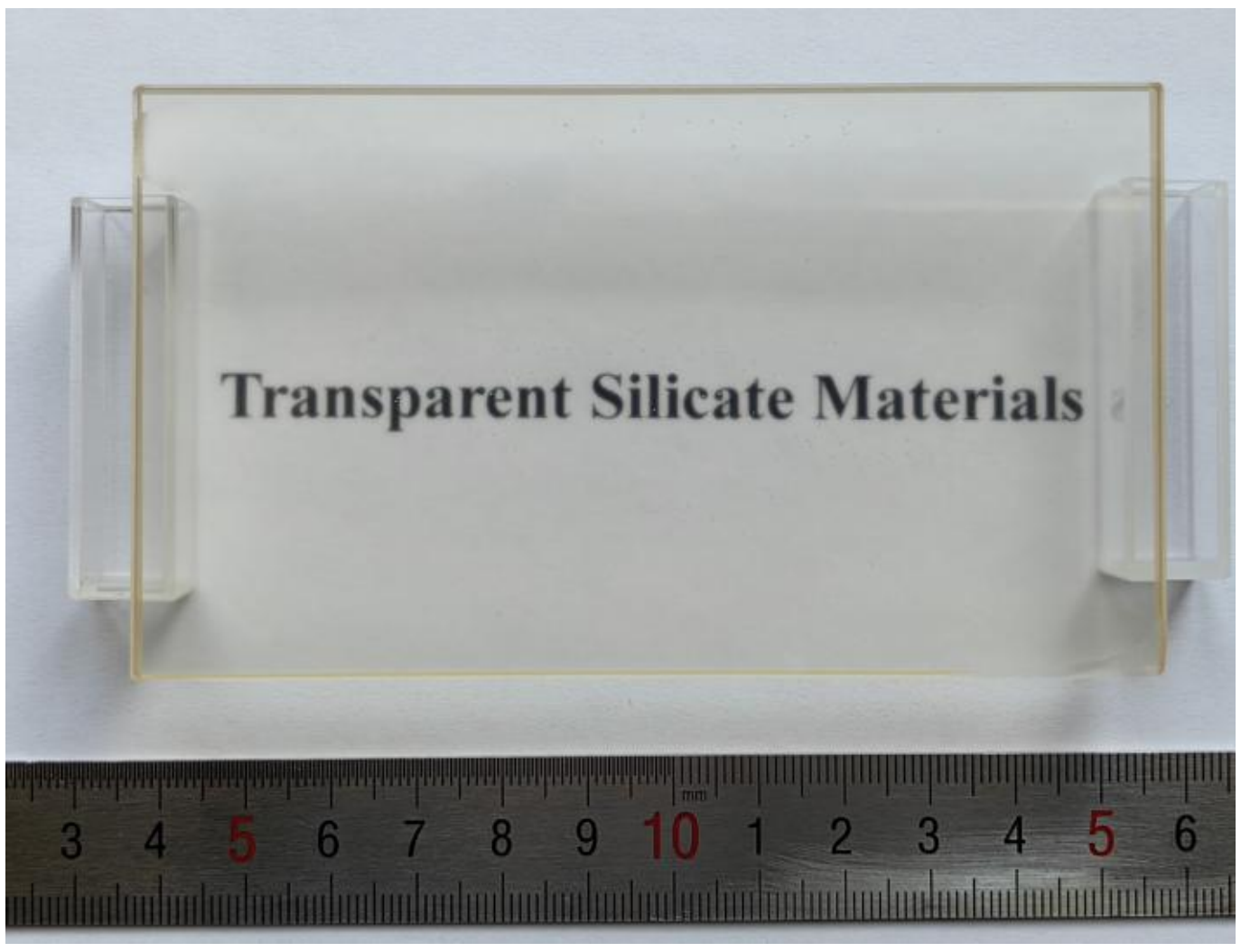

Supplementary Fig. 8. Demonstration of optical transparency. A photograph of the BZ-GC (11.3 cm×6.5 cm × 0.6 cm), positioned 1.5 cm above the underlying text using a transparent cuvette as support, clearly illustrates its high transparency.

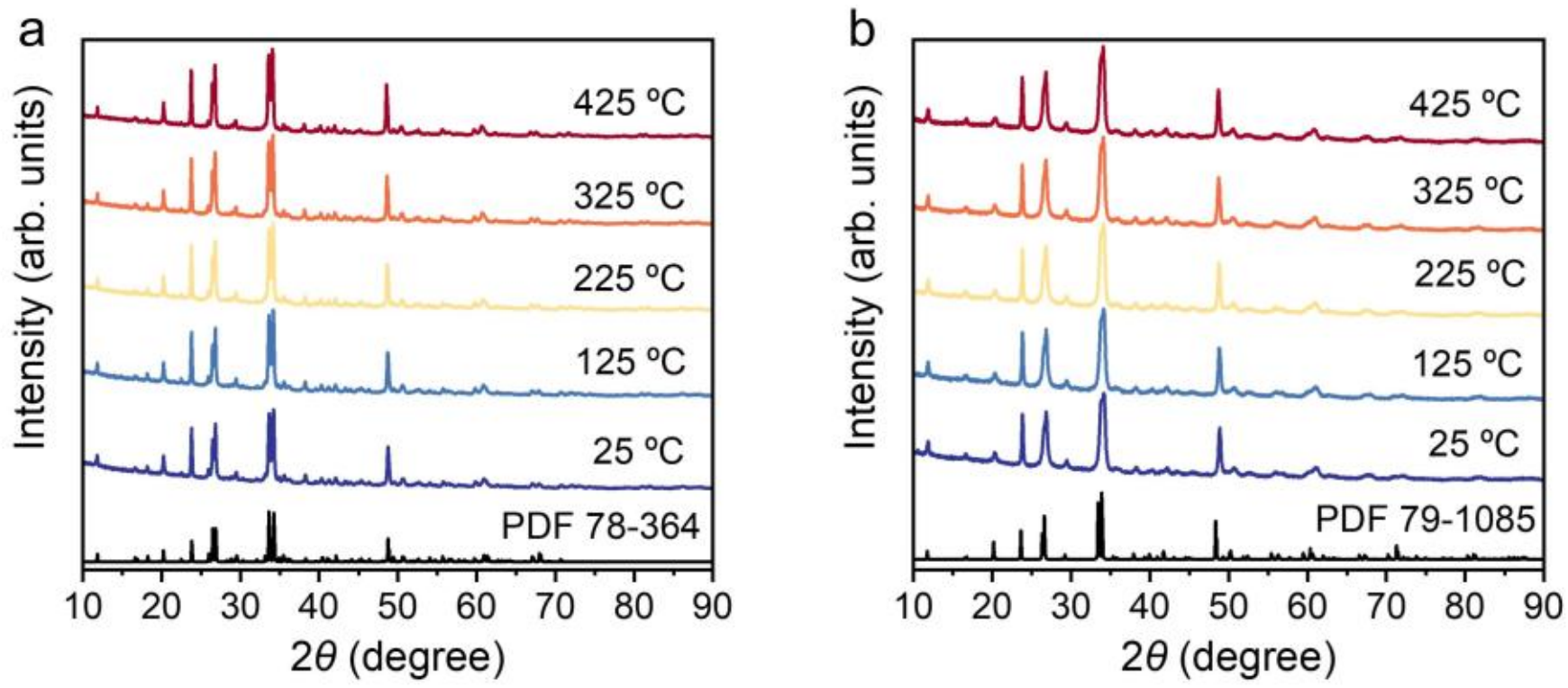

Supplementary Fig. 9. In-situ high-temperature XRD analysis. XRD patterns collected in situ from 25 °C to 425 °C for (**a**) B-GC (nucleated at 600 °C for 20 h and crystallized at 670 °C for 20 h) and (**b**) BZ-GC (nucleated at 630 °C for 20 h and crystallized at 720 °C for 20 h). Reference patterns for the low-temperature (PDF#78-364) and high-temperature (PDF#79-1085) polymorphs of $Na_4Ca_4Si_6O_{18}$ are plotted at the bottom for comparison. Source data are provided as a Source Data file.

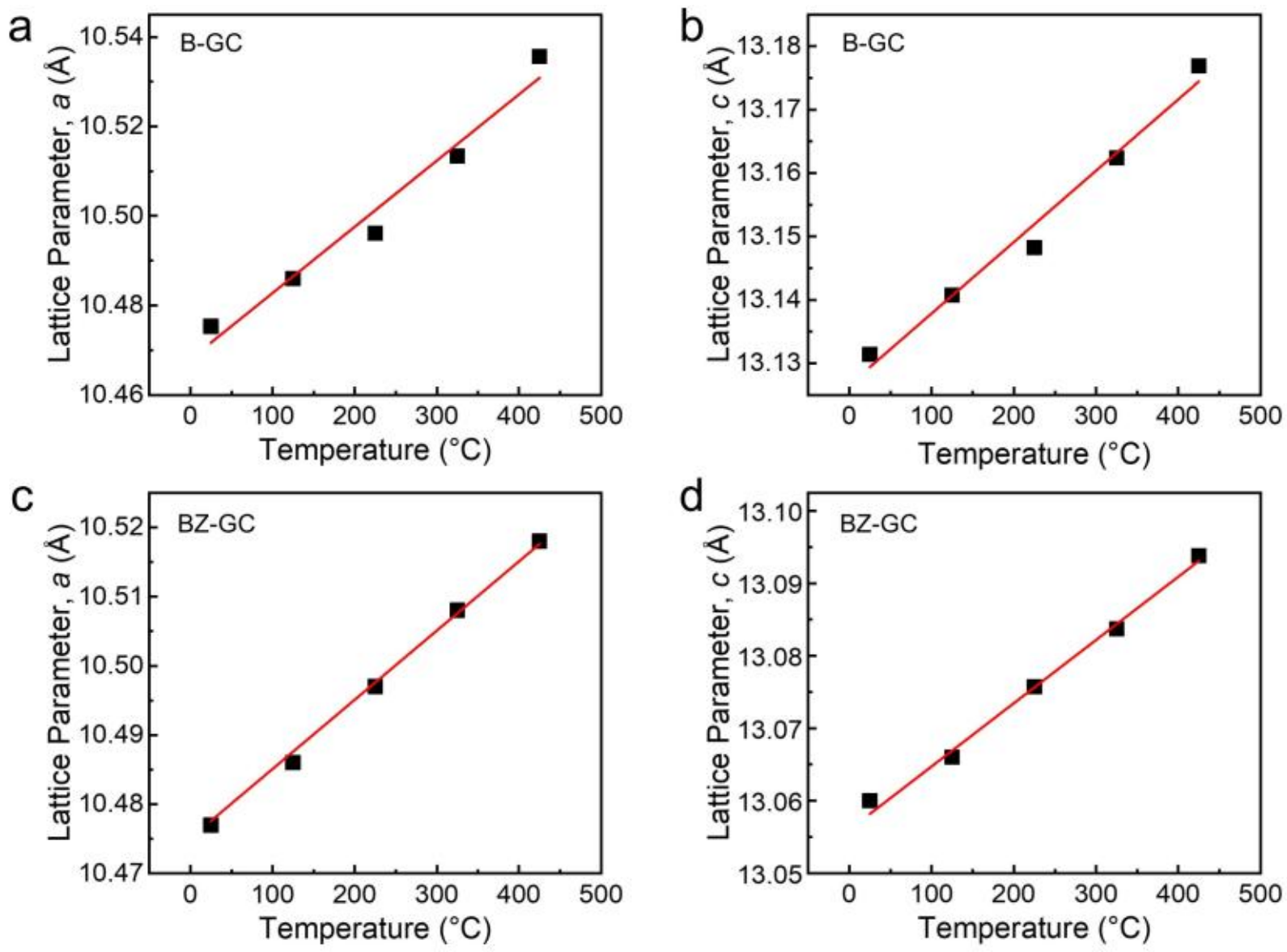

Supplementary Fig. 10. Temperature dependence of the unit cell parameters. a, **b** Evolution of the lattice parameters (*a* and *c*) of B-GC (nucleated at 600 °C for 20 h and crystallized at 670 °C for 20 h). **c**, **d** Corresponding lattice-parameter changes for the BZ-GC (nucleated at 630 °C for 20 h and crystallized at 720 °C for 20 h). Red lines represent linear fits to the thermal expansion trend. Source data are provided as a Source Data file.

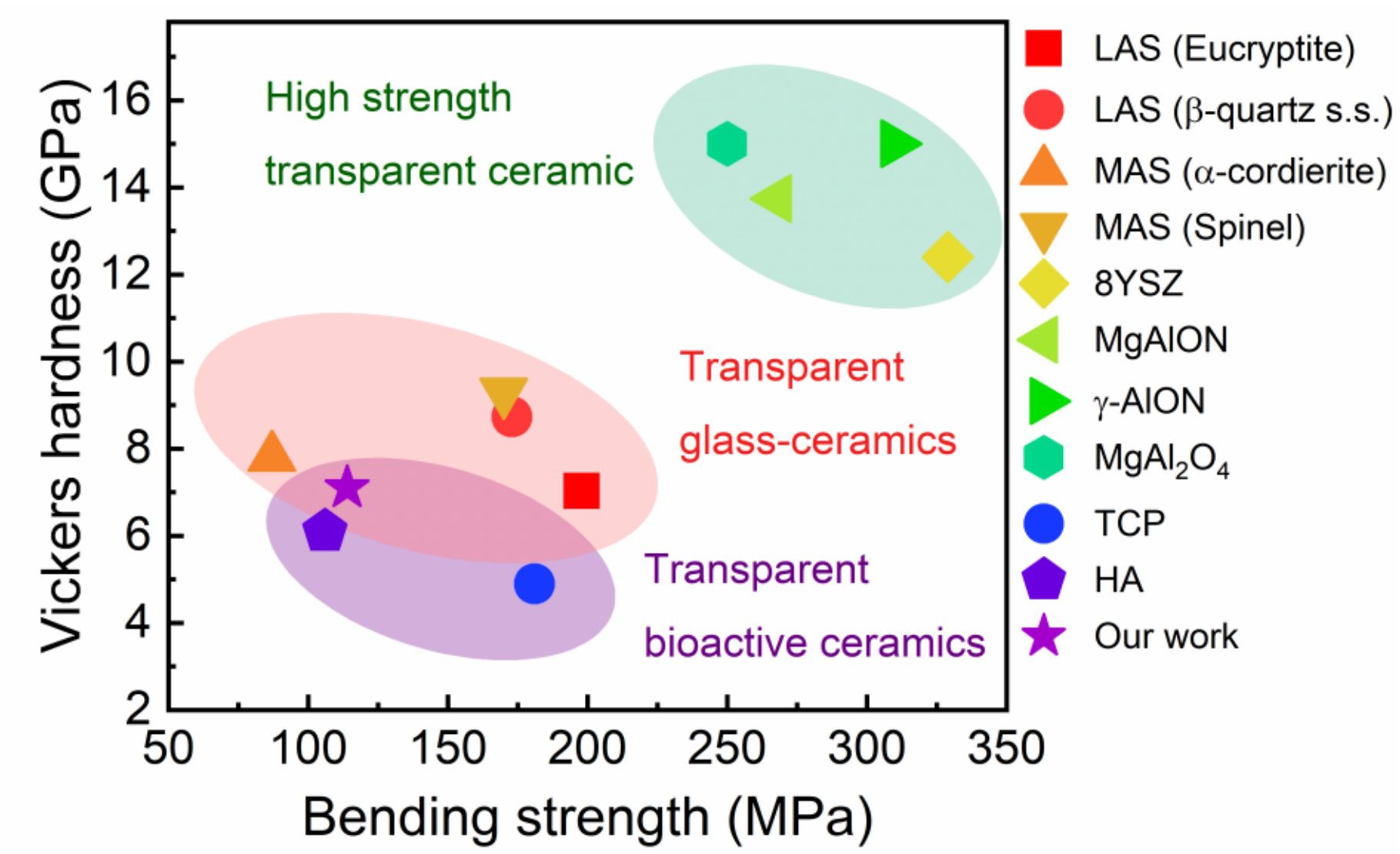

Supplementary Fig. 11. Comparative mechanical performance. The Vickers hardness and three-point bending strength of the BZ-GC are compared with those of conventional transparent ceramics, glass-ceramics, and bioactive ceramics (reference data are listed in the main text). Source data are provided as a Source Data file.

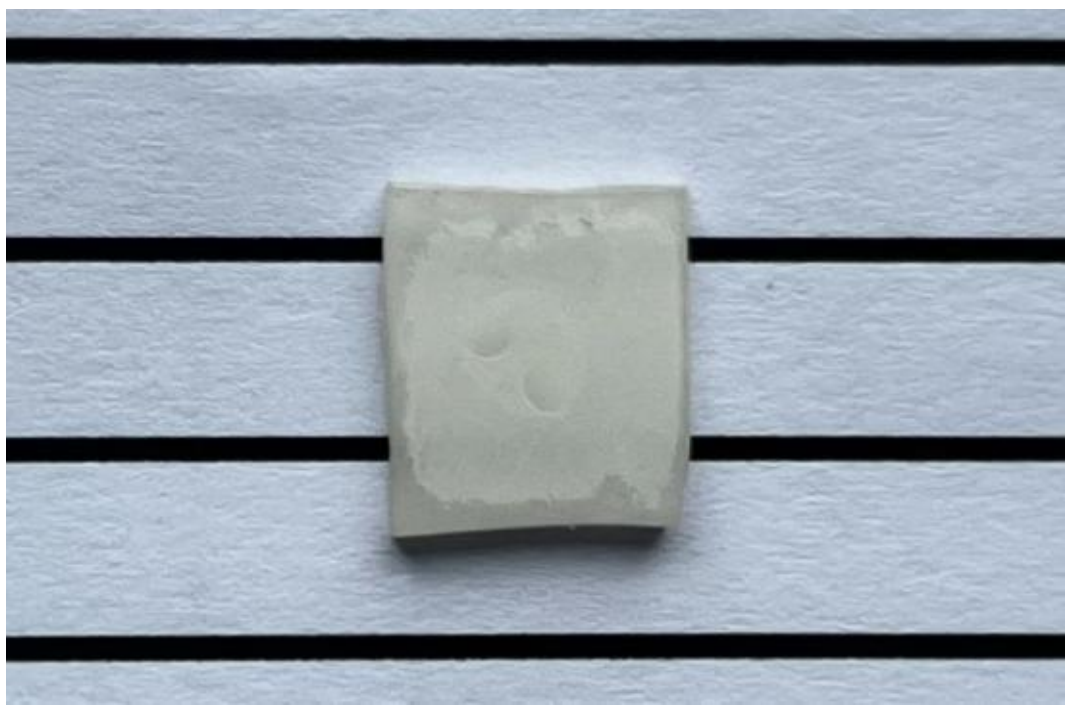

Supplementary Fig. 12. Surface appearance after SBF immersion. Photograph of the BZ-GC specimen following immersion in simulated body fluid (SBF) for 7 days at 37 °C to assess hydroxyapatite formation.

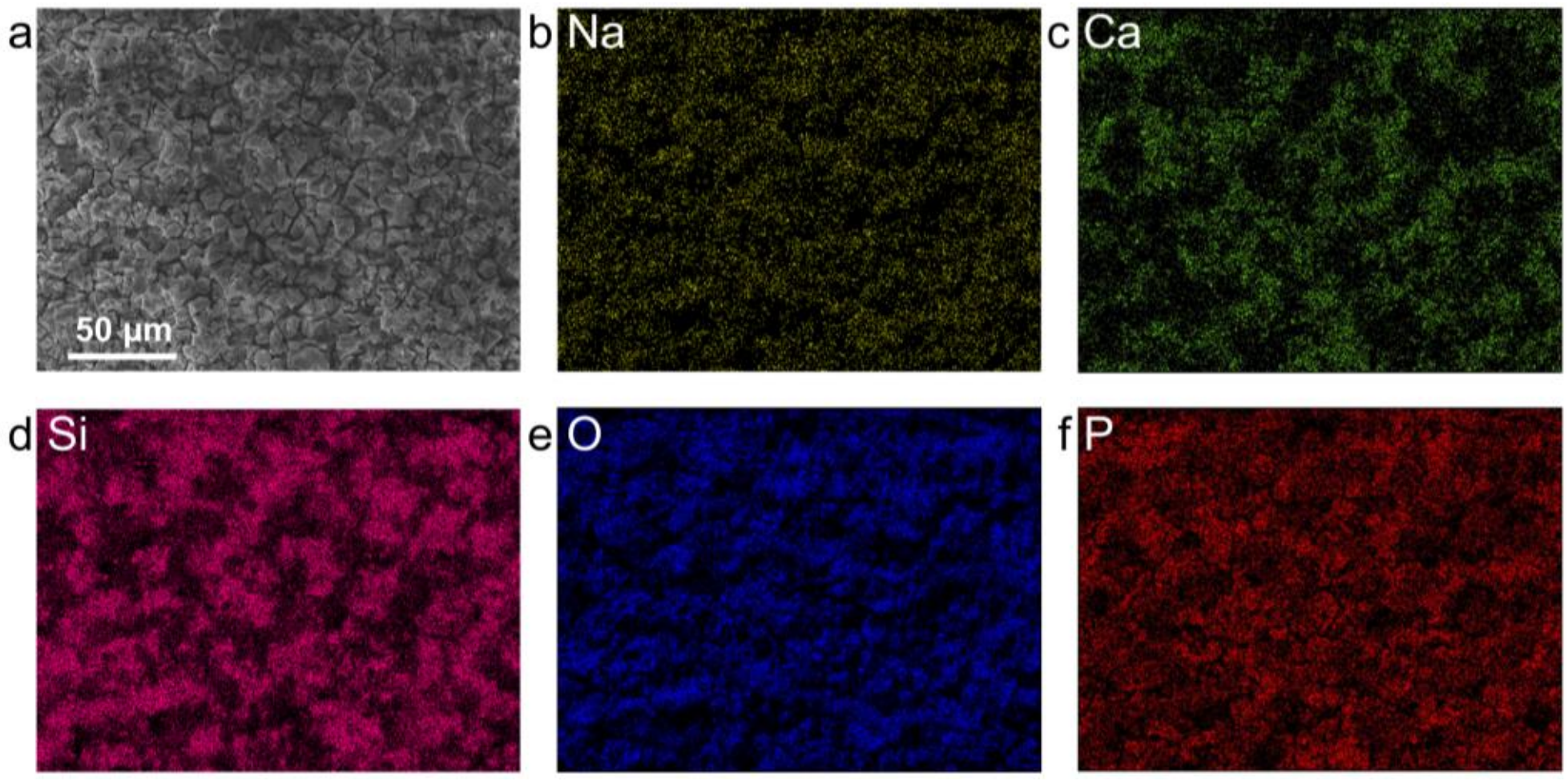

Supplementary Fig. 13. Surface morphology and elemental distribution after SBF immersion. a FE-SEM image (SE) of the BZ-GC surface following immersion in simulated body fluid (SBF). Corresponding EDS maps show the distribution of (**b**) Na, (**c**) Ca, (**d**) Si, (**e**) O, and (**f**) P in the region outlined in (**a**).

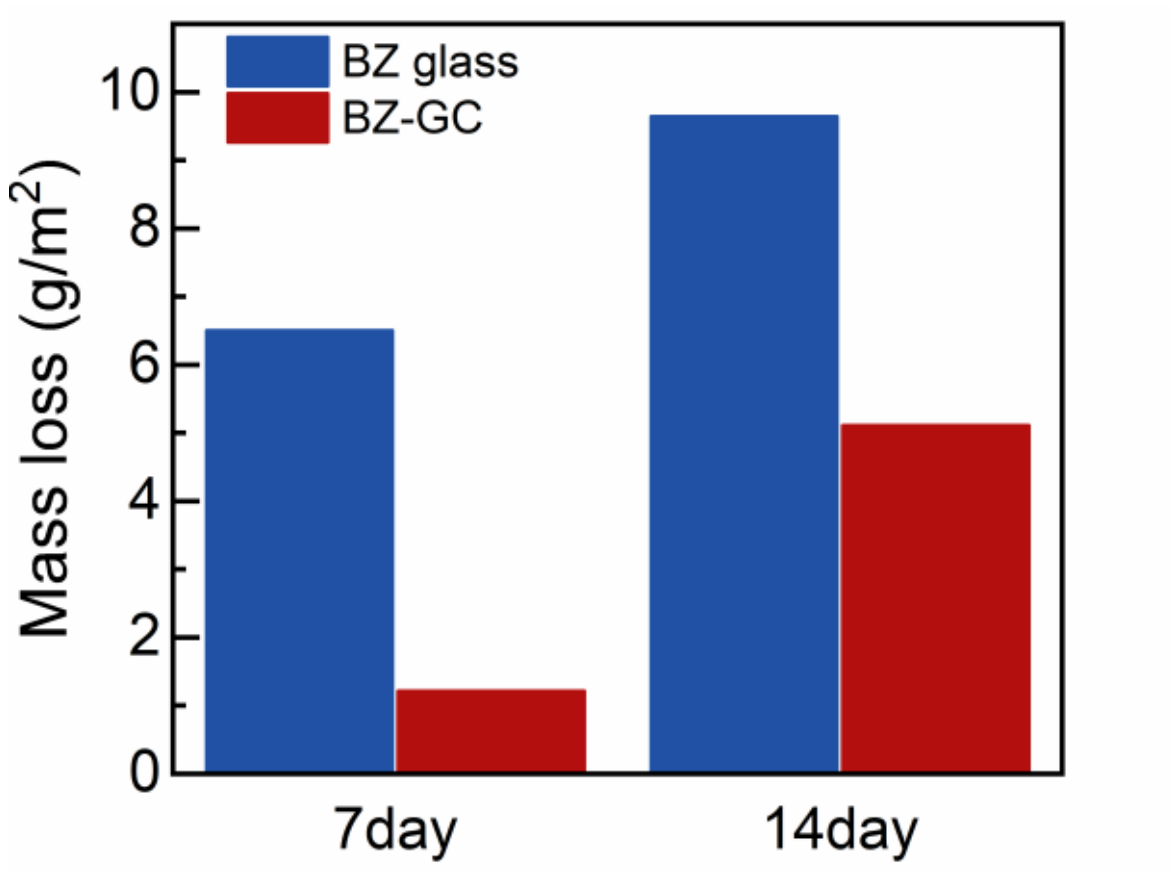

Supplementary Fig. 14. Hydrolytic stability in aqueous environment. Mass loss per unit surface area of BZ glass and BZ-GC samples after immersion in deionized water at 37 °C over periods of 7 days and 14 days. Source data are provided as a Source Data file.

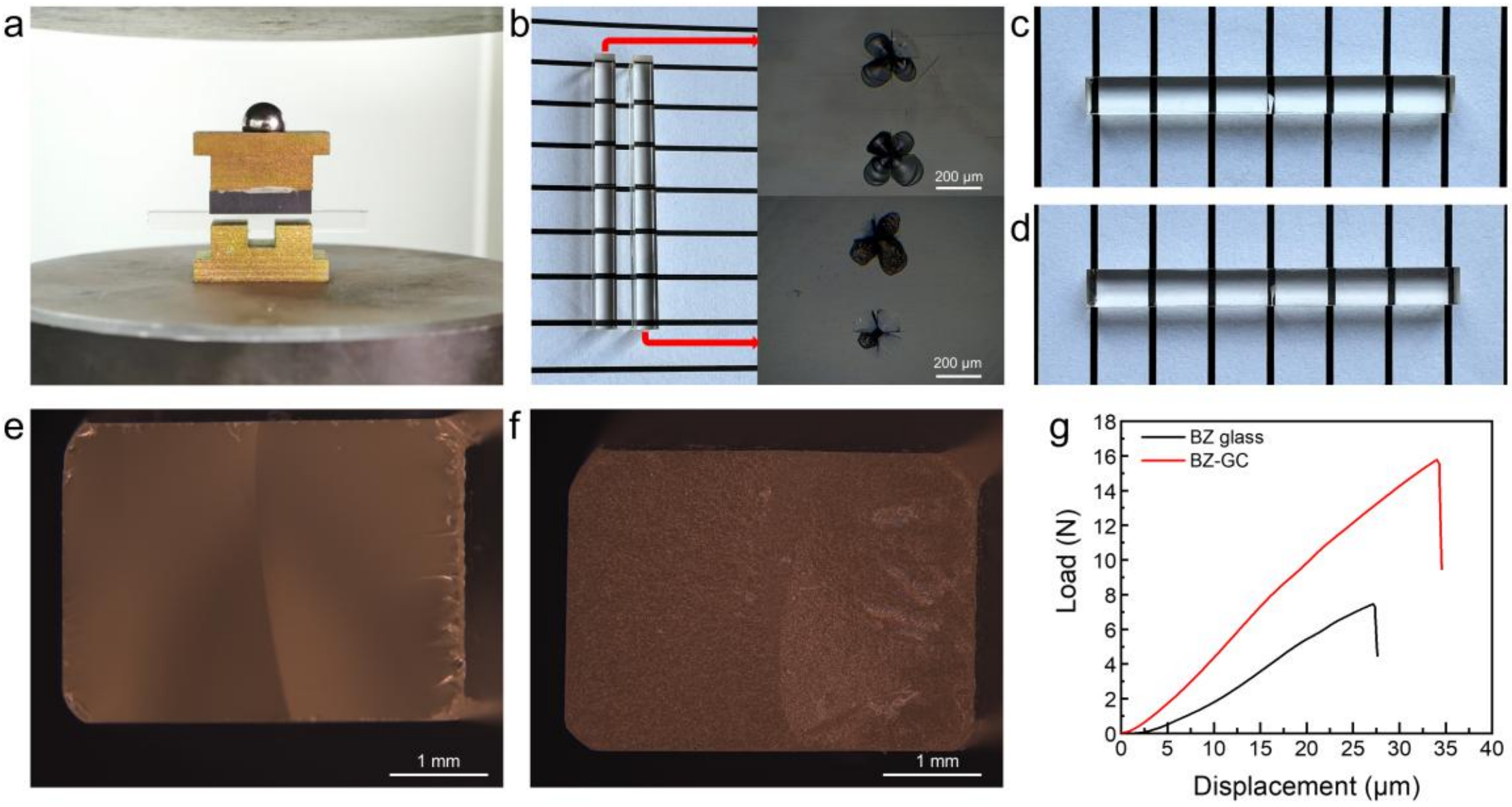

Supplementary Fig. 15. Detailed procedure of the single-edge precracked beam (SEPB) test. a Bridge compression fixture with the sample positioned between the upper (silicon carbide) and lower (high-carbon alloy steel) parts. **b** Vickers indentation lines on BZ glass (left) and BZ-GC (right) samples; the right insets show magnified views of the indentations produced under a load of 1 kgf (~9.8 N). **c**, **d** Precracks in the BZ glass (**c**) and BZ-GC (**d**) samples after bridge compression. **e**, **f** Fracture surfaces of the BZ glass (**e**) and BZ-GC (**f**) samples after three-point bending test with the precrack fronts clearly indicated. **g** Representative load-displacement curves recorded during the SEPB test for both materials. Source data are provided as a Source Data file.

As shown in Supplementary Fig. 15a, the upper part of the bridge compression fixture (in contact with the sample) is made of silicon carbide, and the lower part is made of high-carbon alloy steel; both components possess extremely high stiffness. Vickers indentations were used as efficient precrack precursors under a load of 1 kgf (~9.8 N). The load holding time was 10 s for the BZ glass sample and 20 s for the BZ-GC sample. Supplementary Fig. 15b displays the indentation lines on the BZ glass (left) and BZ-GC (right) samples, with magnified views of the indentation imprints provided as insets. Supplementary Fig. 15c,d show the precracks in the BZ glass and BZ-GC samples, respectively, after bridge compression.

A three-point bending fixture (identical to that used for flexural strength testing) was used to fracture the precracked samples at a cross-head speed of 0.2 mm/min. The bending span (S_0) was 28 mm. Supplementary Fig. 15e,f show the fracture surfaces after bending testing, where the precrack fronts are clearly visible. The average precrack lengths were 2.02 mm for the BZ glass sample (Supplementary Fig. 15e) and 1.90 mm for the BZ-GC sample (Supplementary Fig. 15f). Load-displacement curves are presented in Supplementary Fig. 15g. The maximum load (P_{max}) was 7.45 N for the BZ glass sample and 15.77 N for the BZ-GC sample. According to the calculation formulas specified in ASTM C1421-99, the fracture toughness values were approximately 0.7 MPa·m$^{1/2}$ for the BZ glass and 1.4 MPa·m$^{1/2}$ for the BZ-GC sample. Five specimens

for each material were tested, yielding average fracture toughness values of 0.8 ± 0.3 MPa·$m^{1/2}$ for BZ glass and 1.5 ± 0.2 MPa·$m^{1/2}$ for BZ-GC samples.

Supplementary Table 1. ^{29}Si MAS NMR deconvolution parameters. Isotropic chemical shift (δ_{iso}^{CS}) and full width at half maximum (FWHM) values for precursor B and BZ glasses, B-GC, and BZ-GC. Q^n(glass) denotes the [SiO_4] tetrahedra in the glass phase. Q^n is the standard notation in silicate spectroscopy, where *n* (ranging from 0 to 4) indicates the number of bridging oxygen atoms per [SiO_4] tetrahedron. $Q^2(i)$ (i = 1, 2, 3) refers to three distinct subsets of Q^2 units within the six-membered silicate ring structure.

Samples	Species	δ_{iso}^{CS} (ppm)	FWHM (ppm)
B glass	Q^1 (glass)	-74.5±0.3	4.0±1.0
	Q^2 (glass)	-80.5±0.7	10.4±0.8
	Q^3 (glass)	-89.8±0.2	10.7±0.3
B-GC	Q^n (glass)	-86.1±0.2	14.2±0.3
	$Q^2(1)$	-88.0±0.1	3.3±0.3
	$Q^2(2)$	-90.2±0.1	3.3±0.3
	$Q^2(3)$	-92.6±0.1	3.3±0.3
BZ glass	Q^1 (glass)	-74.5±0.3	8.0±0.6
	Q^2 (glass)	-80.5±0.6	11.4±0.2
	Q^3 (glass)	-89.8±0.3	13.2±0.4
BZ-GC	Q^n (glass)	-85.0±0.3	8.3±0.5
	$Q^2(1)$	-89.3±0.2	3.2±0.3
	$Q^2(2)$	-92.1±0.3	3.2±0.3
	$Q^2(3)$	-95.5±0.3	3.2±0.3
