# Supplementary material for: Scalable fabrication of transparent sodium calcium silicate glass-ceramics by structural engineering overcoming anisotropy barriers
Source: Nat Commun. 2026 Jul 10;17:8531. doi: 10.1038/s41467-026-75474-y (PMC13482340; doi:10.1038/s41467-026-75474-y)
Supplement: Supplementary file 2 [file 41467_2026_75474_MOESM2_ESM.pdf]

nature portfolio

Peer Review File

Scalable fabrication of transparent sodium calcium silicate glass-ceramics by structural engineering overcoming anisotropy barriers

Corresponding Author: Professor Chao Liu

Parts of this Peer Review File have been redacted as indicated to remove third-party material.

This file contains all reviewer reports in order by version, followed by all author rebuttals in order by version.

Version 0:

Reviewer comments:

Reviewer #1

(Remarks to the Author)
The authors report on the synthesis and structural characterisations of large transparent alkali silicate ceramics elaborated by crystallisation from glass.
Even though very close materials were reported by Berthier et al., the work is quite original and well presented. In my opinion this work will raise interest of not only people interested in glass crystallisation but also to a broader readership interested in functionalise ceramics. I stipulate that this work will generate follow-on research to describe in details the (micro)structure of these materials and to develop functionalities.
However, before the manuscript can be considered further for publication several points raised below must be considered. I hope that these comments can help to increase further the quality of the manuscript.
- The authors state that the $Na_4Ca_4Si_6O_{18}$ phase crystallises. However this is not exact as there are substitutions operating in this structural phase. Moreover the nominal molar composition ((1-(x+y)/100)[49.27SiO2-34.48CaO-16.25Na2O]-xZrO2-yB2O3) does not match this formulae (even without considering ZrO_2 and B_2O_3). Can the authors clearly establish the composition of the crystalline phase they synthesise. Also they should comment why non stoichiometry (or a nominal composition non matching the crystalline phase mentioned) was considered.
- The authors chose to call their crystalline material a ceramic but they state that a glass phase remains in the final material. I guess that the term 'glass-ceramic" should be used then. How much glass remains in the final material? I reckon deeper microstructural investigations should be performed to clear this point.
- The microstructure is a very important factor governing transparency (but also physical properties such as mechanical properties). However I think that it is unsufficiently described in this manuscript. Only large scale SEM images are provided. A multi-scale analysis should be performed. For example the authors could focus on the presence or absence of porosity. Indeed they state that anisotropic CTE leads to porosity but there are no porosity characterisation reported in their work. Also I reckon that an EBSD study would clearly illustrate the glass crystallisation mechanism at ply in these materials. Last I think that a proper TEM study would be required here (along with a thin foil sample preparation) to focus on the grain boundaries (residual glass phase, possible different composition leading to birefringence effects, etc.). EELS could be envisaged to work out boron localisation and role.
- The authors mention CTE anisotropy as the origin of transparency loss in ceramics elaborated by glass crystallisation. I am very surprised that they do not mention birefringence effect, especially as their crystalline phase is not cubic! To me this point should be discussed and if possible the birefringence should be estimated.
- As it is written in the manuscript, I understand that the XRD data were only recorded up to 90° (Cu radiation) for Rietveld refinement. Why did the authors skip high angle information as this is where occupation information is located?
- Are the elaborated 'ceramic" materials stable in water? I think that this point should be made clear given the proposed applications (and the large Na content).
- Among the proposed applications laser and mechanics are proposed. This seems a bit enthusiastic given that the sample is not cubic (birefringence effect) and the crystals are micrometer size.
- The authors use Tp for the maximum of the crystallisation peak on their DSC data. I guess p stands for peak but Tc would seem more appropriate (also please make clear that it is not onset).
- Abstract section: the authors state "we resolved three core challenges: achieving critical thermal expansion isotropy

control...". Actually the authors report 2 composition and note that there is a difference in CTE values. In my opinion this cannot be named "control". They also claim to have produced "record-sized (transparent) ceramics". This is not true as larger transparent ceramics have been elaborated. In the case the authors mean by crystallisation from glass then this should be mentioned and large scale tellurite ceramics have been reported (as cited by the authors).
- In the introduction section the authors state that current glass crystallisation reports require exceptionally high melting temperatures and rapid cooling rates. Once again I remind the authors that strontium alumiosilicate and tellurite compositions have been reported (in the case of tellurite the melting T is lower than the one used by the authors).
- Figure 5a: a photograph of the sample not lying on the text (let's say rather standing a few centimeters from the text) would clearly illustrate better the transparency of these materials. This figure could be added in SI section

Reviewer #2

(Remarks to the Author)
The manuscript of Liu and co-workers presents a method for producing transparent soda-lime silicate ceramics by incorporating ZrO_2 and B_2O_3 into melt-formed glass precursors, followed by controlled crystallization. The authors claim that Zr^{4+} substitution at Ca^{2+} sites reduces thermal expansion anisotropy and stabilizes the high-temperature $Na_4Ca_4Si_6O_{18}$ polymorph, enabling ceramics with >84% visible transmittance and length up to 11 cm. Mechanical and biocompatibility tests suggest potential for biomedical and high-temperature applications. The work builds on that of Berthier et al. [ref 14] and should be of interest to researchers working on transparent ceramics and glass-ceramics, but in my view, it lacks the innovation and novelty required for publication in a multidisciplinary journal such as Nature Communications.

The authors refer to their samples as ceramics (which would indicate 100% crystallinity), but aren't they glass-ceramics (i.e., with a small residual glass phase)? And if they are fully crystalline, what is the advantage of this compared to a glass-ceramic? In any case, several statements by the authors about novelty are, to the best of my knowledge, incorrect. First, they claim to have produced "record-sized ceramics (11.3 cm × 6.5 cm × 0.6 cm) with >84% visible transmittance". However, Corning, Schott and other glass companies produce transparent (>90% transmittance) glass-ceramics in much larger sizes, which have already been used on commercial products (e.g. smartphones) for many years. For example, see the production information sheet of Corning Gorilla Glass Ceramic and DOI: 10.1002/adem.202200350. Second, statements such as "first viable pathway from alkali containing glass to transparent ceramics" and "commercially viable glass compositions typically contain alkali elements, which fundamentally impede controlled crystallization" are then also incorrect, as the composition studied in the article cited above is based on lithium. Third, it is not clear to me how the authors can support the claim of a "transformative approach" when they use the traditional approach of "melt-forming followed by controlled thermal treatment". Fourth, the work builds on Berthier et al., but what is fundamentally new beyond scaling and Zr doping? Characterization of additional samples with varying Zr content would strengthen the claim of anisotropy control. The structural origin of how Zr substitution modifies thermal expansion behavior and stabilizes the high-temperature polymorph is also not provided.

Additional suggestions for clarification and improvement.

- The authors are correct that crystallization of a precursor glass can "minimize pore formation during the crystallization", but it is not a guarantee. What has the authors done to confirm that the present samples are fully dense and do not contain a significant fraction of pores?

- The glass transition in the "B glass" is not obvious (Supp. Fig. 1). Also, is there a second crystallization peak in this sample at around 950 C? More detailed characterization is needed.

- Why does the 11B MAS NMR data have so low signal-to-noise ratio? This is not typically observed in such samples. I was also surprised that the fitted boron coordination numbers were not reported.

- The assignment of the crystal phase based on PDF card 79-1085 seems inconsistent with all the peaks in the crystallized samples (Fig. 4a). Specifically, the authors' samples have a relatively strong peak at around 61-62 degrees, which does not appear in the PDF card.

- The authors refer to their samples as "crack-free" but only minimal mechanical characterization of the samples is reported. Most importantly, fracture toughness testing should be performed (using a self-consistent method, i.e. not indentation) to confirm that the samples have the required damage tolerance for the proposed applications, such as "transparent armor". Comparison of the mechanical properties (including hardness and bending strength) should also be done to other ceramics and glass-ceramics.

- Similarly, to evaluate the samples' potential for biomedical applications, specifically the comparison to 45S5 Bioglass, requires testing of their ability to precipitate hydroxyapatite.

- The chemical composition of the melt-quenched samples should be tested (e.g. using ICP-OES) as especially B_2O_3 and Na_2CO_3 are prone to evaporate at high temperature and B_2O_3 is very hygroscopic. Also, is it correct that the so-called "B glass" has zero B_2O_3 as stated in the Methods section (i.e. "x=0, y=0: B glass")?

Reviewer #3

(Remarks to the Author)
This study represents a substantial advance in transparent ceramics research by demonstrating a melt-forming approach to

fabricate centimeter-scale sodium calcium silicate transparent ceramics—a longstanding challenge for alkali-containing systems. The work holds significant applied value through its utilization of industrial glass manufacturing techniques to achieve unprecedented specimen dimensions (11.3 cm ×6.5 cm) while maintaining dual functionality (biocompatibility and fire resistance). Academically, it provides novel insights into crystallization control in alkali glasses through strategic Zr^{4+} substitution, establishing a new paradigm for anisotropy reduction via crystallographic site engineering. The correlation between structural modification and functional properties offers valuable design principles for advanced optical materials.
I recommend Minor Revision to enhance the manuscript's impact and clarity. The study is well-conceived and experimentally thorough, but would benefit from the following specific improvement:
1.The reasons for selecting a nucleation temperature of 600 °C and a crystallization temperature of 700 °C for 20 h for B glass, as well as a nucleation temperature of 620 °C and a crystallization temperature of 720 °C for 20 h for BZ glass. Meanwhile, please reconfirm whether the nucleation temperature for BZ glass is 620 °C or 630 °C to ensure consistency with the temperature indicated in the legend.
2.Please standardize the graph preparation by using different colors to distinguish Q2(1), Q2(2), and Q2(3) in Fig. 3c, and provide an explanation for the meaning of Qn.
3.The image results for (iv) 2 h and (v) 4 h in Fig. 4b are similar. It is recommended to replace (v) with the image for 20 h.
4.Provide full UV-Vis-NIR transmission spectra rather than single-wavelength data to better evaluate optical performance for practical applications.
The manuscript is generally well-written and the data support the conclusions. The combination of scalability, dual functionality, and fundamental mechanistic insight makes this work highly suitable for Nature Communications. With the requested minor revisions, this study will provide a valuable contribution to the materials science community and offer tangible pathways for industrial translation.

Version 1:

Reviewer comments:

Reviewer #1

(Remarks to the Author)
The authors have made a subsequent effort to answer the reviewers' remarks. To me there is one major point/question remaining : should the sample be named a ceramic or a glass-ceramic?
To me this question can be answered by referring to the 2018 JNCS paper cited by the authors: if the authors can observe some remaining glass in their sample then it is a glass-ceramic and if there is no more glass in the sample (full crystallisation process) then it is a ceramic. I would not refer to the book from the 1960s cited by the authors in their response letter (characterisation tools have much developed since then). At the bottom of page 14 of their response letter they claim that both GC an C materials can contain glass. I don't agree with that and this is in contradiction with the recent definition of a GC.
Also a material cannot be defined depending on the process used to prepare it (behind is the idea that a glass-cermaic sample should be elaborated by crystallisation from glass). There is a simple reason for this: if you find a material (in nature for example) and have no idea how it was processed then you cannot name this material. Also the JNCS 2018 paper mentions GC materials elaborated differently.
The 2018 JNCS paper managed to reach a consensus between many academic researchers and industrials regarding the definition of a GC (TC7 committee), I reckon this is the definition to use today.
Now, is there or not glass in the authors' sample? From electron microscopy it seems that there is not. I understand that the authors also performed quantitative amorphous phase content by XRD but really I would not trust much this approach as it is not precise, especially in this case where the authors did not determine exactly the population on each crystallographic site. If the authors stand on saying that there is glass in their sample then they must change their claims regarding "largest ceramic sample prepared..." etc. as compared to existing glass-ceramic sample (see exmaple cited by reviewer 2) their sample is not any larger or more transparent.
I hope these comment can help to further improve the manuscript.

Reviewer #2

(Remarks to the Author)
I thank the authors for their responses and revisions. For me, the conceptual novelty is still much too limited to warrant publication in Nature Communications. It was also very hard for me to follow the authors' logic in their response regarding glass-ceramic vs. ceramic. They state that "Given the presence of a glassy phase, the BZ "ceramic" should indeed be termed as a "glass-ceramic".", but still insist to refer to it as a ceramic… If a ceramic phase contains a residual glass phase, it is per definition a glass-ceramic, i.e., it is incorrect to state that "both material classes can contain a glass phase". Transparent glass-ceramics with better properties, larger sizes and made with similar raw materials are already fully established in the glass/ceramic literature and indeed already on the market. I think this alone proves my point of limited novelty, since the authors are not proposing a new technique/concept to produce such materials and microstructures, and they do not offer any advantages compared to the existing materials. Even if we follow the authors' argument that the novelty comes because the present materials are "ceramics", this does not make it novel since the improved performance is still due to the same/similar microstructures as can and have been induced in glass-ceramics and formed by the same process of heat-treatment.

Minor point: regarding the SEPB testing, the authors provide only very minimal details on how these experiments were done. Please show images of the bridge compression setup (stiffness of this is setup is e.g. crucial), indentations used to make precrack, precrack front variation, and load-displacement curves.

I think the work is of interest to other researchers working on glass-ceramics and deserves publication in a materials journal focusing on glasses and ceramics, such as Journal of the American Ceramic Society or Acta Materialia.

Reviewer #3

(Remarks to the Author)
The authors have satisfactorily addressed all the comments and suggestions raised during the review process. The revisions made have significantly improved the clarity and quality of the manuscript. Therefore, I recommend the manuscript for acceptance in its current form.

Version 2:

Reviewer comments:

Reviewer #1

(Remarks to the Author)
The authors have made appropriate modifications to the manuscript. I am now happy with the clarifications regarding the materials (glass-ceramic vs ceramic).
In the end the material developed here is very similar to the one reported by Berthier et al (2006). Could the authors comment on the requirement to use a nominal composition different from the aimed crystalline phase? I guess this composition difference explains the glass-ceramic nature of the material (full and congruent crystallisaiton is not possible). In a way developing a ceramic material would have been a clear progress and differentiation to Berthier's work.

Reviewer #2

(Remarks to the Author)
I thank the authors again for their responses and revisions. My opinion remains that the conceptual novelty is too limited for publication in Nature Communications given the existing literature on transparent and tough glass-ceramics, including commercial products. However, at least the current manuscript is now much clearer, follows the correct terminology and some claims have been toned down.

Open Access This Peer Review File is licensed under a Creative Commons Attribution 4.0 International License, which permits use, sharing, adaptation, distribution and reproduction in any medium or format, as long as you give appropriate credit to the original author(s) and the source, provide a link to the Creative Commons license, and indicate if changes were made.
In cases where reviewers are anonymous, credit should be given to 'Anonymous Referee' and the source.
The images or other third party material in this Peer Review File are included in the article’s Creative Commons license, unless indicated otherwise in a credit line to the material. If material is not included in the article’s Creative Commons license and your intended use is not permitted by statutory regulation or exceeds the permitted use, you will need to obtain permission directly from the copyright holder.

To view a copy of this license, visit https://creativecommons.org/licenses/by/4.0/

Response to Reviewers

Dear Reviewers,

We are sincerely grateful for your thorough and insightful review of our manuscript. Your constructive comments and thoughtful suggestions have been invaluable in guiding us to improve the quality and clarify of our work.

In response to your feedback, we have conducted extensive additional experiments and further verification, including complementary microstructural analyses, quantitative assessment of residual glass content, full-range optical transmittance measurements, hydrolytic stability tests, and detailed mechanical characterization. We have also refined our interpretations and clarified several key points regarding terminology, novelty claims, and structural mechanisms. We hope that the newly provided data and revised text address your concerns and strengthen the manuscript significantly.

Should any remaining points of divergence or ambiguity persist, we remain fully open to further discussion and are prepared to undertake additional revisions based on your guidance to ensure the manuscript meets the standards of the journal.

Once again, we deeply appreciate your time, expertise, and invaluable assistance in advancing this work.

Sincerely,

Chao Liu

On half of all co-authors

Wuhan University of Technology

Reviewer #1 (Remarks to the Author):

The authors report on the synthesis and structural characterisations of large transparent alkali silicate ceramics elaborated by crystallization from glass. Even though very close materials were reported by Berthier et al., the work is quite original and well presented. In my opinion this work will raise interest of not only people interested in glass crystallization but also to a broader readership interested in functionalise ceramics. I stipulate that this work will generate follow-on research to describe in details the (micro)structure of these materials and to develop functionalities. However, before the manuscript can be considered further for publication several points raised below must be considered. I hope that these comments can help to increase further the quality of the manuscript.

(1) The authors state that the $Na_4Ca_4Si_6O_{18}$ phase crystallises. However this is not exact as there are substitutions operating in this structural phase. Moreover the nominal molar composition ((1-(x+y)/100)[49.27SiO2-34.48CaO-16.25Na2O]-xZrO2-yB2O3) does not match this formulae (even without considering ZrO2 and B2O3). Can the authors clearly establish the composition of the crystalline phase they synthesise. Also they should comment why non stoichiometry (or a nominal composition non matching the

crystalline phase mentioned) was considered.

Response: We thank the reviewer for raising these essential points regarding the precise composition and stoichiometry of the crystalline phase. The reviewer is correct in both observations, and we appreciate the opportunity to provide clarification.

The reviewer is right that the crystalline phase is not ideal $Na_4Ca_4Si_6O_{18}$. As indicated in the manuscript (and in prior work, e.g., Berthier et al.), the lovozerite-type phase in this system is more accurately described as a solid solution with the formula $Na_{4+2x}Ca_{4-x}[Si_6O_{18}]$ ($0 \leq x \leq 1$). In our BZ ceramic specimen, this is further modified by cationic substitutions: Zr^{4+} partially replaces Ca^{2+} on the M(4) site, and Na^+ may also substitute for Ca^{2+} on the M(1) and M(3) site. Therefore, the phase is a Zr-bearing derivative of the Na-Ca-silicate solid solution.

While direct atom-scale chemical analysis was hindered by the material's beam-sensitivity (as discussed in response to Comment 3), we performed quantitative Rietveld refinement on the BZ ceramic. The refinement, which accounts for site occupancies, yields an estimated crystalline phase composition of $Na_{4.506}Ca_{3.435}Zr_{0.156}Si_6O_{18}$. This result confirms the significant incorporation of Zr and the non-stoichiometry ion Na/Ca ratio. We have revised the relevant descriptions in the manuscript (highlighted in red). (Line 23-27, Page 8)

The deviation from the simple $Na_2O{\cdot}2CaO{\cdot}3SiO_2$ stoichiometry was intentional and based on the two key considerations. Our design strategy aimed to stabilize the high-temperature polymorph, which exhibits lower thermal expansion anisotropy. Cationic substitution (especially Zr^{4+}) and a non-stoichiometric Na/Ca ratio are essential for achieving this stabilization, as they influence the site occupancy and lattice energy. The nominal composition was adjusted based on melting trials and the analysis of the actual glass composition via ICP-OES. The measured glass compositions are slightly enriched in Na_2O, and the Si/Ca molar ratio is close to 1.5. This adjusted composition was found to promote the homogeneous melting, improve the glass stability and ultimately yield a transparent ceramic after prescribed thermal treatment.

In summary, the non-stoichiometry is a deliberate component of our materials design, enabling both the stabilization of the desired crystal structure and the fabrication of a dense, transparent material. We believe these clarifications accurately reflect the synthesized phase and justify our compositional choices. We have added a sentence in the manuscript to briefly explain this design rationale.

"To ensure the compositional accuracy, the as-annealed glasses were analyzed by inductively coupled plasma-optical emission spectrometry (ICP-OES, Prodigy 7, LEEMAN, USA). The measured compositions (in mol.%) were 48.57 SiO_2-32.61 CaO-17.63 Na_2O-1.19 B_2O_3 for the B glass and 47.49 SiO_2-32.21 CaO-17.26 Na_2O-1.69 ZrO_2-1.35 B_2O_3 for the BZ glass. Both glasses show a slight enrichment in Na_2O relative to the stoichiometric $Na_2O{\cdot}2CaO{\cdot}3SiO_2$ formulation, with Si/Ca molar ratios close to 1.5. This deliberate off-stoichiometry was adopted to stabilize the high-temperature polymorph and to tailor the glass-forming ability and crystallization, thereby enabling the fabrication of transparent ceramic." (Line 25, Page 11 to Line 5,

Page 12)

(2) The authors chose to call their crystalline material a ceramic but they state that a glass phase remains in the final material. I guess that the term 'glass-ceramic" should be used then. How much glass remains in the final material? I reckon deeper microstructural investigations should be performed to clear this point.

Response: Thank you for your valuable suggestions.

Firstly, let me reply to the crystallinity issue. For quantitative Rietveld refinement, 30 wt% rhombohedral α-Al_2O_3 was added to the powder samples as an internal standard and thoroughly mixed by a mortar. The results (Supplementary Fig. 4) show that crystalline phase is around 93 wt% and glassy phase is around 7 wt% by the calculation of Rietveld refinement. Both SEM and TEM (Supplementary Fig. 3a and also discussion shown below) observation show the BZ ceramic is composed of multiple close packed grain, and no obvious glass phases along the grain boundaries was observed. TOF-SIMS (Supplementary Fig. 5a) and LA-ICP-MS spectroscopy analysis results (Supplementary Fig. 5b) evidenced that the glassy phase probably locate at the grain boundaries.

Regarding the terms “glass-ceramics” or “ceramics”, both Reviewer #1 and Reviewer #2 raised similar concerns. These concerns are very important and highly instructive. We hope to engage in a constructive discussion on the difference between glass-ceramics and ceramics and, based on this discussion to have an opportunity to revise the manuscript accordingly.

First, is the presence of a glassy phase the criterion for differentiating glass-ceramics from ceramics? Concerning glass-ceramics, we completely agree with the reviewers’ opinion. The International Commission on Glass has provided an updated definition: “Glass-ceramics are inorganic, non-metallic materials prepared by controlled crystallization of glasses via different processing methods. They contain at least one type of functional crystalline phase and a residual glass. The volume fraction crystallized may vary from ppm to almost 100%” (J. Deubener et al., J. Non-Cryst. Solids, 2018, 501, 3-10). Given the presence of a glassy phase, the BZ “ceramic” should indeed be termed as a “glass-ceramic”.

However, regarding ceramics, it is also known that ceramics can contain a glassy phase. For example, in Kingery’s classic text, it is stated: Analysis of ceramics shows them to be a mixture of crystalline phases and glasses, each of many different compositions, usually combined with porosity, in a wide variety of proportions and arrangements” (W.D. Kingery, Introduction to Ceramics, Copyright © 1960, 1976 by John Wiley & Sons, Inc.; Page 1). Consequently, the mere presence or amount of a glassy phases may not be a precise point of differentiation.

Second, could the preparation method serve as the distinguishing criterion? We have consulted several experts in the fields of glass-ceramics and ceramics. There a general tendency to consider materials prepared through the crystallization of glass as glass-ceramics, and ceramics are mainly prepared through sintering. Sintering of glass

powders has also been employed to prepare glass-ceramics. those prepared through the sintering of powders as ceramics. Based on the consensus, our BZ material, prepared via crystallization from a glass, should be called a glass-ceramic.

Nevertheless, this distinction is not absolute. Ceramics can also be obtained through the crystallization of glass. For instance, Allix et al. prepared a transparent $BaAl_4O_7$ ceramic via the crystallization of glass (Adv. Mater., 2012, 24, 5570-5575). Furthermore, ceramics can be derived from polymers---polymer-derived ceramics (PDCs). Most PDCs are amorphous after the polymer-to-ceramic transformation, and (poly)crystalline PDCs can be obtained by subsequent annealing of the amorphous ceramics at elevated temperatures (see Q. Wen et al., Prog. Mater. Sci., 2020, 109, 100623), a process analogous to the crystallization of glasses. Therefore, preparation methods alone may not be sufficient to definitively differentiate the two categories.

Given the above discussion, it seems necessary to consider additional distinguishing characteristics. Let us compare the microstructure of glass-ceramics and ceramics, specifically how the residual glassy phases are distributed.

In typical glass-ceramics, crystals are dispersed randomly within a continuous glass matrix. Even in glass-ceramics with a very high crystallinity of 97 vol.% (see Fig. 1 in ref. Cunha et al., Adv. Eng. Mater. 2007, 9, 191), the residual glassy phase remains continuous and irregular in shape (left panel in Fig. R1). In contrast, in conventional sintered ceramics, crystals are closely packed together, and no obvious, continuous glassy phase is easily observed (for example, right panel in Rig. R1). In such ceramics, any residual glassy phase typically resides only at the grain boundaries, forming a very thin amorphous layer (for example, Gilbert et al., J. Am. Ceram. Soc., 1997, 80, 2253-2261).

The microstructure of our BZ ceramic specimen (Fig. 2d) is more similar to that of conventional ceramics, as shown in the right panel of Fig. R1 (from Wagner et al., J. Euro. Ceram. Soc. 2019, 39, 1436-1442). Additionally, in the TEM image (Supplementary Fig. 3), clear grain boundaries were observed, and no continuous and irregular glassy phase was present. Consequently, the microstructure of the BZ ceramic specimen is significantly different from that typical glass-ceramics and aligns more closely with conventional ceramics.

[Figure Redacted]

Fig. R1 Left panel: SEM micrographs showing the typical microstructure of the glass-ceramics (96 vol.% crystallized) with cubic crystals (crystal size >10 µm) (cited from Cunha et al., Adv. Eng. Mater. 2007, 9, 191). **Right panel**: HRSEM images of the

microstructure of sintered cerium-doped YAG ceramics (cited from Wagner et al., J. Euro. Ceram. Soc. 2019, 39, 1436-1442)

Difference in microstructure also lead to distinct fracture surface features. For glasses and fine-grained glass-ceramics, fracture surfaces typically exhibit mirror, mist, and hackle regions (see, for example, Fig. 18.12 and Fig. 18.24 in book-Fundamentals of Inorganic Glasses by A.K. Varshneya, J.C. Mauro, Third Edition; Fig. 1 in Kelly et al., Facture-surface analysis of dental ceramics, The Journal of Prosthetic Dentistry, 1989, 62, 536-541; Fig. R2). As noted by Kelly et al., "Glasses, glass-bonded ceramics, and fine-grained polycrystalline ceramics yield these features. Coarse-grained polycrystalline ceramics and weaker multiphase ceramics do not provide such details."

For conventional ceramics, either intergranular fracture (as seen in the right panel of Fig. R1) or transgranular fracture is observed. The fracture surface of our BZ ceramic specimen (Supplementary Fig. 7a) shows features characteristic of transgranular fracture, not the typical fracture pattern of glasses or glass-ceramics.

[Figure Redacted]

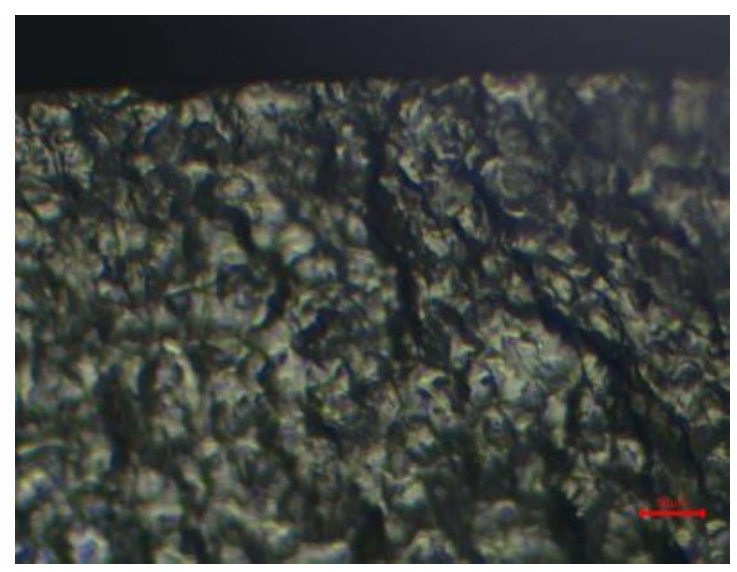

Fig. R2 **Left panel:** Typical facture-surface features (Kelly et al., Facture-surface analysis of dental ceramics, The Journal of Prosthetic Dentistry, 1989, 62, 536-541). **Right panel:** Optical microscope image of fracture surface of BZ ceramic specimen (Scale bar: 50 µm).

In summary, while we agree with the reviewers that our BZ ceramic specimen can logically be called a glass-ceramic based on its formation route, its microstructural and fractographic characteristics are more aligned with those of conventional ceramics. This presents an interesting point for discussion, and we do not intend to dismiss the reviewers' suggestion. If the reviewers have further differing viewpoints or suggestions regarding the terminology, we are willing to reconsider and revise accordingly.

(3) The microstructure is a very important factor governing transparency (but also physical properties such as mechanical properties). However I think that it is unsufficiently described in this manuscript. Only large scale SEM images are provided. A multi-scale analysis should be performed. For example the authors could focus on the presence or absence of porosity. Indeed they state that anisotropic CTE leads to porosity but there are no porosity characterisation reported in their work. Also I reckon that an EBSD study would clearly illustrate the glass crystallisation mechanism at ply in these materials. Last I think that a proper TEM study would be required here (along

with a thin foil sample preparation) to focus on the grain boundaries (residual glass phase, possible different composition leading to birefringence effects, etc.). EELS could be envisaged to work out boron localisation and role.

Response: We are sincerely grateful for the reviewer for these exceptionally constructive and challenging suggestions, which have prompted us to conduct extensive, multi-scale microstructural characterization. We have added substantial new data and discussion to the revised manuscript to address these points.

To verify the absence of significant porosity in the fabricated BZ ceramics, we employed two complementary microscopic techniques:

(1) FE-SEM on freshly fractured surfaces (Supplementary Fig. 7a, b) revealed a dense, coherent microstructure with no detectable pores.

(2) Confocal laser scanning microscopy (CLSM), which has detection limit of roughly <100 nm (Wagner et al., Journal of the European Ceramic Society, 2019, 39, 1436), was used to scan a volume of 100 × 100× 2 μm^3. No pores were observed within this detection limit (Supplementary Fig. 7c).

Together, these observations confirm that the prepared BZ ceramics are fully dense at the micro- and sub-micrometer scale, supporting the statement that crystallization under our optimized conditions effectively reduces pore formation.

In the revised manuscript, the following content was added.

Taking the BZ ceramic specimen (Fig. 2d) as a representative example, both FE-SEM of its freshly fractured surface and confocal laser scanning microscopy (CLSM) of optically polished specimen confirmed a dense, pore-free microstructure (Supplementary Fig. 7). The CLSM analysis, with a detection resolution of roughly <100 nm[29], scanned a three-dimensional volume of 100 × 100 × 2 μm^3 and revealed no evidence of pores (Supplementary Fig. 7c), indicating that pore formation was effectively suppressed. (Line 17-23, Page 7)

In the "Microstructure characterization" section, the following content was added.

To evaluate the presence of pores, the BZ ceramics were examined using complementary microscopic techniques. FE-SEM was performed on freshly fractured surfaces. In addition, confocal laser scanning microscopy (CLSM, Zeiss LSM880, Germany) was employed on optically polished surfaces to detect sub-surface pores, which appear as bright spots against a dark background under the CLSM imaging mode. The spatial resolution of the CLSM system was estimated to be roughly <100 nm. For the CLSM analysis, a series of optical slices (each ~100 × 100 μm^2) were acquired at a depth interval of 400 nm. Multiple slices (acquisition time: 30 s per slice) were then reconstructed to generate a three-dimensional volume of 100 × 100 × 2 μm^3, enabling a volumetric inspection for pores within the detection limit of the instrument. (Line 26, Page 13 to Line 8, Page 14)

Concerning the attempts at advanced microstructural characterization:

We fully agree with the reviewer that the microstructural characterization of the BZ ceramic, especially regarding the structure and composition at the grain boundaries,

is of utmost importance. To this end, we systematically pursued the suggested advanced techniques, including EBSD, TEM, EDS, EELS, LA-ICP-MS, and TOF-SIMS. However, the intrinsic properties of the material—its insulating nature and high alkali content—posed substantial experimental challenges, leading to significant electron-beam-induced damage (e.g., charging, structural degradation, and even local melting) that prevent us from acquiring reliable, high-resolution data. We have documented these beam-sensitivity issues with supplementary video recordings submitted as "Supporting Information for Review".

In the case of EBSD, multiple attempts (more than three separate trials) were made, even with conductive coating applied to the specimens. Nevertheless, no clear diffraction patterns could be obtained, which we attribute to the instability of the crystalline phase under the high-current electron beam required for EBSD acquisition.

For TEM, EDS, and EELS analysis, conventional focused-ion-beam (FIB) preparation method – even with the specimen cooled to –170 ºC – was found to be unsuitable, as the lamella incurred severe damage during milling (see Fig. R3). Moreover, when a lamella containing grain boundaries was thinned to the thickness required for EELS, it became highly prone fracture. Out of six specimens prepared, only one survived the thinning processes; EELS analysis of this lamella did not detect noticeable enrichment of B or Zr at the grain boundaries. We therefore adopted a cryogenic preparation route involving dimple grinding followed by argon-ion milling at liquid-nitrogen temperature. The resulting TEM observation are presented in Supplementary Fig. 3. Low-magnification images (Supplementary Fig. 3a,b) reveal a polycrystalline microstructure with impinging grains and well-defined grain boundaries, consistent with the SEM observations in Fig. 2d. Despite using low-temperature preparation with gentle ion milling, a significant amount of beam-induced damage was still observed in the lamella (Supplementary Fig. 3b). High-resolution imaging across a representative grain boundary (Supplementary Fig. 3c) shows clear lattice fringes on both sides, indicating highly crystalline domains. Fast Fourier transform (FFT) patterns from adjacent grains (Supplementary Fig. 3d,e) confirm that the crystals have structure consistent with the $Na_4Ca_4Si_6O_{18}$ (PDF#79-1085). EDS elemental mapping (Supplementary Fig. 3f) demonstrates a nearly homogeneous distribution of Na, Ca, Si, Zr, and O at the micrometer scale, with no pronounced segregation of any element at the grain boundaries. Attempts to acquire higher-magnification or longer-exposure time led to rapid beam-induced damage, limiting further atomic-scale analysis.

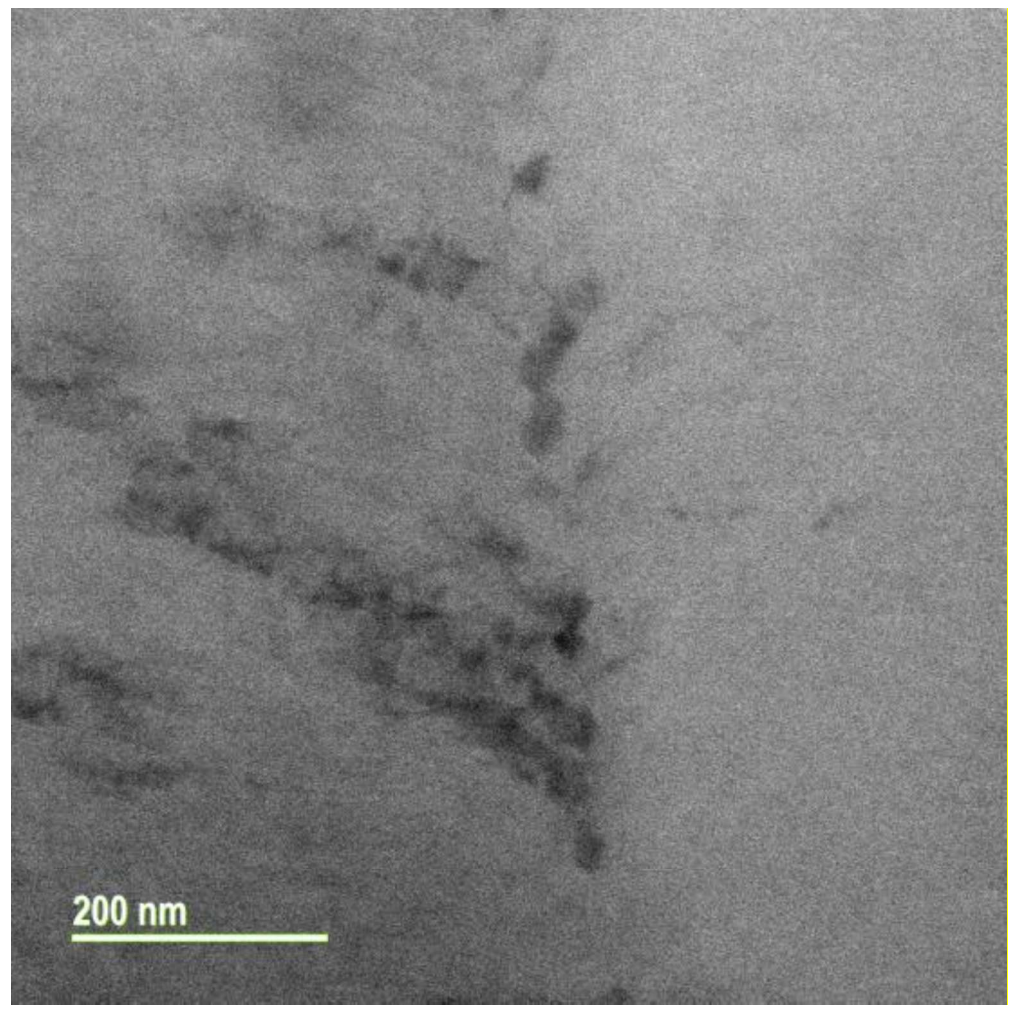

Fig. R3 FIB-prepared TEM lamella of the BZ ceramic. The dark contrast regions are attributed to ion-beam-induced damage incurred during focused-ion-beam (FIB) milling.

TOF-SIMS analysis was also attempted to probe finer elemental distributions, particularly for light element such as boron. However, both techniques were severely hampered by charging effects and sample damage arising from the insulating nature and high alkali content of the material, preventing the acquisition of interpretable spectra or maps (see representative TOF-SIMS data in Supplementary Fig. 5a). These results underscore the pronounced electron- and ion-beam sensitivity of the present alkali-rich silicate ceramics. Despite the charging and potential damage, weak contrast variations in the TOF-SIMS signals suggest a possible segregation of B and Zr at the grain boundaries.

In the case of implementation of LA-ICP-MS:

To circumvent the electron-beam-induced challenges, we employed laser ablation inductively coupled plasma mass spectrometry (LA-ICP-MS). This technique provided chemically specific mapping without charging issues. Even though the spatial resolution is relatively low, the results reveal a clear enrichment of B and Zr along the grain boundaries, offering direct evidence for the segregation of these additives, which aligns with their intended roles in modifying crystallization behavior and reducing anisotropy. This key finding has been integrated into the Supplementary Fig. 5b.

In the revised manuscript, the following sentences are added.

TEM observation of the BZ ceramic specimen revealed clear grain boundaries with sharp interfacial contrast and no obvious presence of a continuous glass phase (Supplementary Fig. 3a,b). HR-TEM imaging and corresponding fast Fourier transformation (FFT) patterns (Supplementary Fig. 3c-e) further confirmed that the grains are highly crystalline and structurally consistent with the high-temperature $Na_4Ca_4Si_6O_{18}$ polymorph. (Line 12-17, Page 5)

Owing to pronounced beam-sensitivity (charging and damage) caused by the

material's insulating nature and high alkali content, energy dispersive X-ray spectroscopy (EDS) mapping (Supplementary Fig. 3f) could not conclusively detect a thin glass phase at the grain boundaries. However, time of flight secondary ion mass spectrometry (TOF-SIMS) mapping (Supplementary Fig. 5a) and inductively coupled plasma mass spectrometry (LA-ICP-MS) analysis (Supplementary Fig. 5b) suggested that the residual glass phase, enriched in B and Zr, is likely distributed along the grain boundaries. (Line 20-28, Page 5)

In the microstructural characterization section, the following information was added:

High-resolution transmission electron microscopy (HR-TEM) was performed on a Talos-F200S microscope (ThermoFisher Scientific, USA). The TEM specimen was prepared by dimple grinding followed by Ar^+ ion milling under liquid-nitrogen cooling to minimize the damage, and was subsequently coated with carbon on both sides to mitigate charging effects during observation. Laser ablation inductively coupled plasma mass spectrometry (LA-ICP-MS) was employed for multi-elemental mapping. The analysis was performed using an Iridia laser system equipped with a 193 nm ArF excimer laser, coupled via an aerosol rapid introduction system (ARIS) to a NexION 300D ICP-MS (PerkinElmer, Norwalk, USA). High-purity helium served as the carrier gas to transport ablated aerosols from the chamber to the ICP-MS. The laser spot size and line-scan spacing were both set to 1 μm. Operating conditions were optimized to a single-pulse response of ~10 ms and a laser repetition rate of 100 Hz. Data integration and image processing were carried out using Iolite software (v3.6) running under Igor Pro 7 (WaveMetrics, USA). Time of flight secondary ion mass spectrometry (TOF-SIMS) imaging in positive-ion mode was performed using a nanaoTOF3 instrument (ULVAC-PHI, Japan) equipped with a Bi^+ primary ion source. (Line 2-18, Page 13).

(4) The authors mention CTE anisotropy as the origin of transparency loss in ceramics elaborated by glass crystallisation. I am very surprised that they do not mention birefringence effect, especially as their crystalline phase is not cubic! To me this point should be discussed and if possible the birefringence should be estimated.

Response: We thank the reviewer for raising this important point. The reviewer is absolutely correct that birefringence in non-cubic crystalline phases can significantly affect the transparency of polycrystalline ceramics.

The primary crystalline phase in our ceramics, $Na_4Ca_4Si_6O_{18}$, belongs to the trigonal system (space group $R\bar{3}m$). As noted by the reviewer, its non-cubic symmetry does introduce birefringence. However, literature reports consistently indicate that the birefringence of this phase is low (E. R. Segnit, Am. J. Sci., 1953, 251, 586-601; Peterson et al., Contrib. Mineral. Petrol., 1989, 101, 458-478). For example, Peterson et al. (Contrib. Mineral. Petrol., 1989, 101, 458-478) cite a value of ~0.003, which is even lower than that of many common glasses. This low intrinsic birefringence means that in a well-densified, pore-free ceramic with uniform grain size, its contribution to light scattering is minimal compared with other factors.

In the revised manuscript, we have revised the relevant sentence in the relevant discussion section (Line 14-15, Page 3) to acknowledge the role of birefringence: "Although the phase is non-cubic, its birefringence is low[13-15]; therefore, mitigating this *a*/*c*-axis thermal expansion anisotropy is therefore essential for obtaining transparent $Na_{4+2x}Ca_{4-x}[Si_6O_{18}]$ ceramics".

We agree with the reviewer that a full quantitative estimation of birefringence scattering in our polycrystalline microstructure would be valuable, and we note this as a direction for future detailed optical modelling. For the present work, the key finding remains that by mitigating CTE anisotropy through Zr^{4+} substitution, we successfully obtained transparent ceramics despite the non-cubic crystal structure, precisely because the intrinsic birefringence of the phase is so low.

(5) As it is written in the manuscript, I understand that the XRD data were only recorded up to 90° (Cu radiation) for Rietveld refinement. Why did the authors skip high angle information as this is where occupation information is located?

Response: We thank the reviewer for this important observation. The reviewer is correct that high-angle XRD data are valuable for refining site-occupancy details. For the Rietveld refinement reported in our study, the XRD pattern was actually measured over the full 2θ range of 10° to 120° (Cu Kα radiation). In the original figures, only the 10° to 90° region was displayed for graphical clarity, as no strong characteristic peaks appear above 90°. In response to the comment, we have revised Fig. 2a and 2b in the manuscript to show the complete diffraction pattern up to 120°, ensuring that all available structural information is visually presented. The refinement itself was performed using the full dataset, and the reported reliability factors reflect the fit across the entire measured angular range.

(6) Are the elaborated 'ceramic" materials stable in water? I think that this point should be made clear given the proposed applications (and the large Na content).

Response: We thank the reviewer for raising this essential point regarding aqueous stability, which is indeed critical for the proposed biomedical and high-temperature applications given material's high alkali content.

In direct response, we conducted hydrolytic stability tests by immersing both the BZ glass and the BZ ceramic specimens in deionized water at 37 °C for periods of 7 and 14 days. The mass loss per unit surface area was quantified as a metric of dissolution (Supplementary Fig. 14). The results show a marked improvement upon crystallization: the BZ glass exhibited mass losses of ~6.5 g/m^2 (7 days) and ~9.6 g/m^2 (14 days), whereas the BZ ceramic showed significantly lower losses of ~1.2 g/m^2 (7 days) and ~5.1 g/m^2 (14 days). This demonstrates that the crystallized material possesses substantially enhanced resistance to aqueous dissolution, which strengthens its suitability for applications requiring environmental stability. It should be noted that bioactive materials do not require absolute stability; rather, a controlled degree of degradability is essential to release ions such as Si^{4+} and Ca^{2+}, which in turn stimulate

bone-cell proliferation and differentiation (M. Schumacher et al., Bioact. Mater., 2021, 6, 1921-1931).

In the main text, the following sentences were added:

Hydrolytic stability tests further confirmed that crystallization significantly improves the material's durability in aqueous environments. The BZ ceramic exhibited a mass loss of only ~5.1 g/m^2 after 14 days in deionized water at 37 °C, compared to ~9.6 g/m^2 for the BZ glass under the same conditions (Supplementary Fig. 14). (Line 27, Page 10 to Line 2, Page 11).

In the "Property measurement" section, the following sentences were added:

Hydrolytic stability was evaluated by immersing polished samples (15 mm × 10 mm × 2 mm) in 50 mL of deionized water with a sealed polytetrafluoroethylene (PTEE) reactor at 37 °C. Prior to immersion, the samples were cleaned, and their surface area and initial mass were recorded. After 7 and 14 days, the samples were removed, dried thoroughly, and reweighed to determine the mass loss per unit surface area. (Line 28, Page 14 to Line 5, Page 15).

(7) Among the proposed applications laser and mechanics are proposed. This seems a bit enthusiastic given that the sample is not cubic (birefringence effect) and the crystals are micrometer size.

Response: We thank the reviewer for this constructive feedback regarding the proposed applications. We acknowledge that the non-cubic nature of the $Na_4Ca_4Si_6O_{18}$ crystals introduces birefringence, and that applications such as laser hosts and transparent armor require specific material properties beyond high transparency.

In terms of laser hosts: While the intrinsic birefringence of $Na_4Ca_4Si_6O_{18}$ is low (E. R. Segnit, Am. J. Sci., 1953, 251, 586-601; Peterson et al., Contrib. Mineral. Petrol. 101, 458-478, 1989), the polycrystalline nature of our ceramics with grain sizes around 30 μm would indeed lead to scattering losses that are detrimental for lasing. Although rare-earth doped sodium calcium silicate materials have been studied, a practical laser has not been demonstrated to data. In light of this, and in agreement with the reviewer's point, we have removed "solid-state laser hosts" from the proposed applications in the revised manuscript.

In terms of the mechanics: The reviewer is correct that our current work does not provide the comprehensive mechanical characterization (e.g., high-impact testing) required to substantiate a claim for armor applications. Therefore, we have also removed the specific reference to "transparent armor" from the manuscript.

The revised focus of the work is now more accurately centered on the demonstrated applications: biomedical implants (supported by ~100% cell viability and hydroxyapatite formation) and fire-resistant/high-temperature windows (supported by thermal stability data above 900 °C). We believe this refined scope more faithfully reflects the evidence presented and the reviewer's guidance.

(8) The authors use Tp for the maximum of the crystallisation peak on their DSC data.

I guess p stands for peak but Tc would seem more appropriate (also please make clear that it is not onset).

Response: We thank the reviewer for this precise suggestion. The reviewer is correct that T_c is a more conventional and clearer notation for the crystallization peak temperature. In response, we have updated both the figure and the main text: in the revised Supplementary Fig. 1, T_p has been relabeled as T_c to explicitly denote the peak (not onset) temperature of crystallization, and all corresponding instances of T_p in the main text have been consistently changed into T_c. Furthermore, the T_g and T_c values for both B and BZ glasses have been updated based on the newly measured DSC curves. We believe this adjustment aligns with normal practice and helps to avoid potential confusion.

(9) Abstract section: the authors state "we resolved three core challenges: achieving critical thermal expansion isotropy control...". Actually the authors report 2 composition and note that there is a difference in CTE values. In my opinion this cannot be named "control". They also claim to have produced "record-sized (transparent) ceramics". This is not true as larger transparent ceramics have been elaborated. In the case the authors mean by crystallisation from glass then this should be mentioned and large scale tellurite ceramics have been reported (as cited by the authors).

Response: We sincerely thank the reviewer for these critical observations, which have prompted us to refine our claims for greater precision and accuracy. We have modified the abstract and corresponding manuscript text accordingly.

Regarding "control" of thermal expansion anisotropy: the reviewer is correct that the simple comparison of two compositions does not, in itself, constitute "control". We have therefore revised the phrasing to more accurately reflect our achievement. The key sentence in the abstract now reads:

"We resolved three core challenges: mitigating thermal expansion anisotropy through Zr^{4+} substitution at Ca^{2+} sites……" (Line 17-18, Page 1)

This change replaces the overreaching term "control" with the more factual description "mitigating", which is supported by the measured reduction in $\Delta\alpha$ and the associated suppression of microcracking.

Regarding "record-sized" ceramics: We apologize for the imprecise and overstated claim. The reviewer is right to note the existence of large transparent ceramics, including those derived from glass crystallization (e.g., tellurite systems). To correct this and properly contextualize our work, we have revised the statement to specify the materials system and fabrication route. The updated text in the abstract is:

"… to produce large sodium calcium silicate ceramics (11.3 cm × 6.5 cm × 0.6 cm) with >84% visible transmittance." (Line 20-21, Page 1)

Furthermore, in the main manuscript, we have clarified that, to the best of our knowledge, these dimensions represent the largest transparent ceramics reported to date for the alkali-containing sodium calcium silicate system fabricated via glass crystallization. The revised text reads:

“Employing identical melt-forming and thermal treatment protocols, we produced transparent SCS ceramics measuring 11.3 cm × 6.5 cm × 0.6 cm (Fig. 5a). To the best of our knowledge, this represents the largest transparent ceramics fabricated from alkali-containing SCS glass via the crystallization route.” (Line 1-5, Page 8)

We believe these revisions address the reviewer’s concerns directly, eliminate overstatement, and frame our contributions within their appropriate technical context. We are grateful for the reviewer’s rigor, which has strengthened the manuscript.

(10) In the introduction section the authors state that current glass crystallisation reports require exceptionally high melting temperatures and rapid cooling rates. Once again I remind the authors that strontium alumiosilicate and tellurite compositions have been reported (in the case of tellurite the melting T is lower than the one used by the authors).

Response: We thank the reviewer for this important clarification and for pointing out the oversight in our original statement. The reviewer is correct that tellurite-based glass systems, such as those referenced in our manuscript (Refs. 5 and 7), can indeed be processed at significantly lower melting temperatures. To accurately reflect the broader literature and address this comment, we have revised the relevant sentence in the introduction to read:

“Most reported precursor glasses for crystallization still require high melting temperatures or rapid cooling rates, which limits their scalability inherent to conventional glass manufacturing.” (Line 17-20, Page 2)

This revision acknowledges the exception of tellurite systems and others while maintaining the core argument regarding the scalability challenges associated with many other glass-ceramic fabrication routes. We appreciate the reviewer’s careful reading, which has helped us present a more precise and balanced background.

(11) Figure 5a: a photograph of the sample not lying on the text (let's say rather standing a few centimeters from the text) would clearly illustrate better the transparency of these materials. This figure could be added in SI section

Response: We thank the reviewer for this helpful suggestion to visually enhance the demonstration of transparency. Following the recommendation, we have prepared an additional photograph in which the BZ ceramic specimen is elevated approximately 1.5 cm above the underlying text using transparent cuvettes as support. This new image, which clearly shows the text readable through the material, has been included as Supplementary Fig. 8. We believe this addition provides a more direct and convincing illustration of the optical quality of the fabricated ceramics.

Reviewer #2 (Remarks to the Author):

The manuscript of Liu and co-workers presents a method for producing transparent soda-lime silicate ceramics by incorporating ZrO_2 and B_2O_3 into melt-formed glass precursors, followed by controlled crystallization. The authors claim that Zr^{4+}

substitution at Ca^{2+} sites reduces thermal expansion anisotropy and stabilizes the high-temperature $Na_4Ca_4Si_6O_{18}$ polymorph, enabling ceramics with >84% visible transmittance and length up to 11 cm. Mechanical and biocompatibility tests suggest potential for biomedical and high-temperature applications. The work builds on that of Berthier et al. [ref 14] and should be of interest to researchers working on transparent ceramics and glass-ceramics, but in my view, it lacks the innovation and novelty required for publication in a multidisciplinary journal such as Nature Communications.

The authors refer to their samples as ceramics (which would indicate 100% crystallinity), but aren't they glass-ceramics (i.e., with a small residual glass phase)? And if they are fully crystalline, what is the advantage of this compared to a glass-ceramic? In any case, several statements by the authors about novelty are, to the best of my knowledge, incorrect. First, they claim to have produced "record-sized ceramics (11.3 cm × 6.5 cm × 0.6 cm) with >84% visible transmittance". However, Corning, Schott and other glass companies produce transparent (>90% transmittance) glass-ceramics in much larger sizes, which have already been used on commercial products (e.g. smartphones) for many years. For example, see the production information sheet of Corning Gorilla Glass Ceramic and DOI: 10.1002/adem.202200350. Second, statements such as "first viable pathway from alkali containing glass to transparent ceramics" and "commercially viable glass compositions typically contain alkali elements, which fundamentally impede controlled crystallization" are then also incorrect, as the composition studied in the article cited above is based on lithium. Third, it is not clear to me how the authors can support the claim of a "transformative approach" when they use the traditional approach of "melt-forming followed by controlled thermal treatment". Fourth, the work builds on Berthier et al., but what is fundamentally new beyond scaling and Zr doping? Characterization of additional samples with varying Zr content would strengthen the claim of anisotropy control. The structural origin of how Zr substitution modifies thermal expansion behavior and stabilizes the high-temperature polymorph is also not provided.

Response: We sincerely thank the reviewer for the thorough and constructive evaluation of our work. The reviewer raises critical points regarding terminology, novelty, and mechanistic depth, which we have carefully considered. Below, we provide a point-by-point response and outline the corresponding revision made to the manuscript.

Both Reviewers #2 and #1 have raised a shared concern regarding the terminology of "glass-ceramics" versus "ceramics." We acknowledge that both material classes can contain a glass phase, and we agree that the mere presence of a glass phase or the specific processing route alone may not suffice to definitively distinguish them. In our view, the microstructural arrangement-specifically, how the glass phase is distributed within the material-together with the characteristic fracture surface morphology, provide additional criteria for differentiation. A detailed discussion on this point is provided in our response to Comment 2 of Reviewer #1, which we hope clarifies our perspective.

We sincerely appreciate the reviewer's attention to this point and welcome the opportunity for further instructive discussion. It is our hope that a consensus can be reached whereby the BZ specimens (e.g., those heat-treated at 630 °C for 20 h and 720 °C for 20 h or longer) may be appropriately regarded as ceramics rather than glass-ceramics, based on their observed microstructure and fracture behavior. If a consensus proves difficult to achieve, we remain ready to adjust the terminology based on your further guidance.

Converting the BZ glass into transparent ceramics exhibits several distinct advantages. First, the BZ ceramic exhibits higher transmittance in the visible spectrum. This is primarily attributed to the reduced anisotropy in CTE, achieved through partial substitution of Zr^{4+} for Ca^{2+} at the M(4) site and the enhanced occupation of Na^{+} in M(1), M(2), and M(3) site. This effectively suppresses microcrack formation and enhances optical homogeneity. Second, the BZ ceramic possesses a higher phase transition temperature. This results from the stabilization of the high-temperature polymorph induced by the aforementioned cationic substitutions and off-stoichiometric design, making it more suitable for high-temperature applications such as fire-resistant windows. Third, converting the glass into transparent ceramic significantly improves mechanical properties, including Vickers hardness, fracture toughness, and Young's modulus. These enhancements stem not only from the high degree of crystallinity but also from the dense and uniform microstructure. Finally, the crystallization process also markedly improved the chemical stability of the precursor glass. These properties collectively highlight the integrated advantages of BZ ceramics in optical, thermal, mechanical, and biocompatible performance, extending the potential of SCS systems for use as transparent components.

In terms of novelty and record size.

Regarding the question of novelty, we have revised the original statement the "record-sized ceramics (11.3 cm × 6.5 cm × 0.6 cm) with >84% visible transmittance" into "to produce large sodium calcium silicate ceramics (11.3 cm × 6.5 cm × 0.6 cm) with >84% visible transmittance". This adjustment is made because we believe the specimens we prepared can be classified as ceramics, based on their microstructure and fracture-surface characteristics, which closely resemble those of conventional ceramics.

To our knowledge, previously reported transparent **ceramics** derived from glass crystallization are alkali-free. The reviewer is correct that companies such as Corning and Schott have commercialized large, transparent glass-ceramics containing alkali ions, which have been widely used in consumer products for years. In our view, however, these commercial products are glass-ceramics, not ceramics in the stricter microstructural sense. For instance, in the paper cited by the reviewer (DOI: 10.1002/adem.202200350), the crystals in the heat-treated specimens (850 °C for 5 min) do not show a close-packed microstructure, and a continuous, irregular glass phase is clearly visible (the light-grey regions in their Fig. 2). Similarly, even in highly crystalline (96 vol.%) sodium calcium silicate glass-ceramics (Cunha et al., Adv. Eng. Mater. 2007, 9, 191; see also Fig. R1 in our response to Comment 2 of Reviewer #1), a

continuous and irregular glass phase remains evident.

These microstructures differ markedly from that our BZ specimen, where close-packed grains are observed with no visually distinct glass phase at the boundaries (Fig. 2d and 3b in the manuscript). Therefore, we consider the BZ material to represent a distinct structural class-ceramics derived from an alkali-containing glass precursor-rather than a conventional glass-ceramic.

In terms of "First viable pathway" and alkali challenge.

The reviewer correctly notes the existence of lithium-based glass-ceramics. Our intended point was that commercially dominant SCS glasses (constituting >80% of global production) have historically been considered incompatible with the fabrication of highly crystalline, transparent monoliths due to the challenges of uncontrolled crystallization and anisotropic cracking. Our work demonstrates a viable compositional and processing strategy to overcome these barriers specifically within this ubiquitous, alkali-containing system. The relevant sentence has been revised for clarity: "which fundamentally impede controlled crystallization for transparent ceramics." (Line 24-25, Page 2)

In terms of "transformative" nature of the approach.

Regarding the "transformative" nature of our approach, while the individual processing steps – melt-forming and controlled crystallization – are well established, their integrated application to commercial SCS glass delivers a transformative outcome. The key advances are as follows:

Scalability: We employ standard, large-scale glass melting (without the need for rapid quenching) and conventional thermal treatments, enabling the fabrication of large monolithic samples from industrial SCS glass.

Material transformation: The process converts SCS glass into a highly crystalline, transparent ceramic with significantly enhanced optical and mechanical properties.

Compositional innovation: In most previously reported glass-to-ceramic conversions, the compositions are strictly limited to elements that fully incorporate into the crystalline phase, which minimizes residual glass but also restricts the approach to very few systems. In contrast, our strategy intentionally introduces extrinsic elements (Zr, B) to modify the crystal structure and reduce thermal expansion anisotropy. Although this leads to a minor intergranular glass phase, the obtained ceramic retains high transparency. It is noteworthy that the addition of small amounts of sintering aids – which often form a glassy grain-boundary phase – is a common practice in conventional transparent-ceramic sintering to achieve full densification and improved transparency. Our approach follows a similar principle: rather than avoiding any glass phase, we focus on whether superior overall performance (transparency, thermal stability, mechanical strength) can be attained. Thus, we believe the term "transformative" is well-justified, and our findings offer valuable insights for both transparent-ceramic and glass-ceramic research.

In terms of mechanistic insight beyond scaling and Zr doping.

The reviewer rightly calls for a deeper mechanistic understanding. Our work

advances beyond Berthier et al. not only in scaling but also in providing a functional design strategy:

Anisotropy mitigation: We demonstrate that targeted Zr^{4+} substitution at the Ca^{2+} site, coupled with non-stoichiometric Na/Ca ratios, directly reduces thermal expansion anisotropy (Δα) by 42%, which is the key to suppressing microcracking at high crystallinity.

Polymorph stabilization: The same substitutions stabilize the high-temperature polymorph, which exhibits a lower intrinsic anisotropy.

Composition-property link: Rietveld refinement suggests a crystalline phase composition of ~ $Na_{4.506}Ca_{3.435}Zr_{0.156}Si_6O_{18}$. The Na-enrichment and Zr incorporation make the reversal to the low-temperature polymorph unfavorable, explaining the high thermal stability observed (~910 °C phase transition).

To strengthen this claim, we have included additional data for a sample with higher ZrO_2 content (x=4, y=2) here (Fig. R4), confirming that the high-temperature polymorph is maintained even when slight porosity begins to appear. We have revised the original statement into "a 437 °C elevation in transition temperature confirming that [ZrO_6] substitution and non-stoichiometric Na/Ca ratios effectively stabilized the high-temperature phase" (Line 2-4, Page 9) to more explicitly link the cationic substitution to the observed reduction in anisotropy and polymorph stabilization.

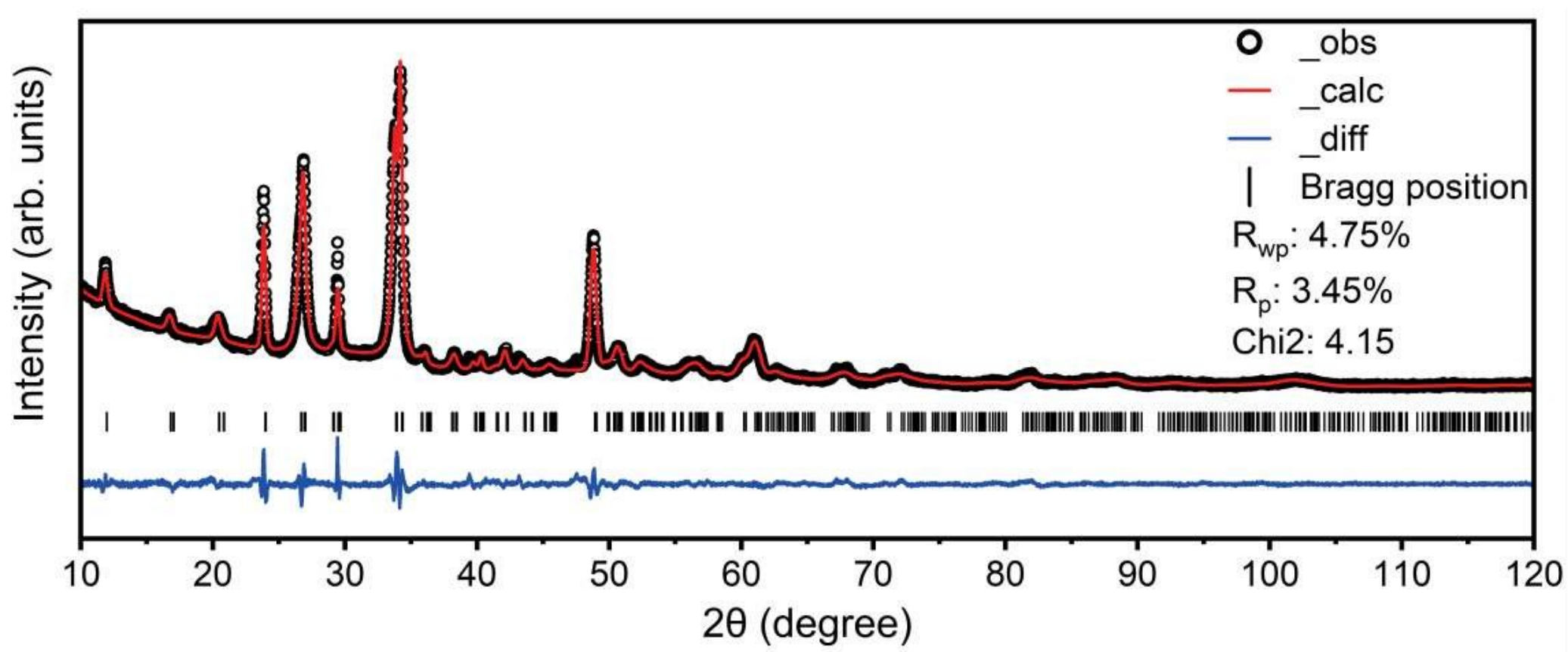

Fig. R4. Rietveld refinement profiles of powdered X-ray diffraction patterns for crystallized glass with x=4, y=2. The heat treatment condition is 660 °C/20 h for nucleation and 760 °C/20 h for crystallization. The refinement results indicate that the XRD profile of sample matches with high-temperature polymorph of $Na_4Ca_4Si_6O_{18}$ (PDF#79-1085).

Additional suggestions for clarification and improvement.

(1) The authors are correct that crystallization of a precursor glass can "minimize pore formation during the crystallization", but it is not a guarantee. What has the authors done to confirm that the present samples are fully dense and do not contain a significant fraction of pores?

Response: We thank the reviewer for this insightful comment and the opportunity to

clarify our efforts regarding densification. The reviewer is correct that the glass crystallization does not automatically ensure a pore-free microstructure; pore formation is influenced by multiple factors such as dissolved gases, crystal size, density mismatch between phases, and the viscosity of the residual glass.

In this study, we systematically screened Na_2O-CaO-SiO_2 glasses with varying contents of B_2O_3 and ZrO_2. We found that higher B_2O_3 content aids in pore suppression, whereas excessive ZrO_2 can promote pore formation-in extreme cases leading to interconnected porosity (a subject for further study). The final composition (BZ glass) was optimized to balance these effects.

To verify the absence of significant porosity in the fabricated BZ ceramics, we employed two complementary microscopic techniques:

(1) FE-SEM on freshly fractured surfaces (Supplementary Fig. 7a, b) revealed a dense, coherent microstructure with no detectable pores.

(2) Confocal laser scanning microscopy (CLSM), which has detection limit of roughly <100 nm (Wagner et al., Journal of the European Ceramic Society, 2019, 39, 1436), was used to scan a volume of $100 \times 100 \times 2\ \mu m^3$. No pores were observed within this detection limit (Supplementary Fig. 7c).

Together, these observations confirm that the prepared BZ ceramics are fully dense at the micro- and sub-micrometer scale, supporting the statement that crystallization under our optimized conditions effectively reduces pore formation.

In the revised manuscript, the following content was added.

Taking the BZ ceramic specimen (Fig. 2d) as a representative example, both FE-SEM of its freshly fractured surface and confocal laser scanning microscopy (CLSM) of optically polished specimen confirmed a dense, pore-free microstructure (Supplementary Fig. 7). The CLSM analysis, with a detection resolution of roughly <100 nm[29], scanned a three-dimensional volume of $100 \times 100 \times 2\ \mu m^3$ and revealed no evidence of pores (Supplementary Fig. 7c), indicating that pore formation was effectively suppressed. (Line 17-23, Page 7)

In the "Microstructure characterization" section, the following content was added.

To evaluate the presence of pores, the BZ ceramics were examined using complementary microscopic techniques. FE-SEM was performed on freshly fractured surfaces. In addition, confocal laser scanning microscopy (CLSM, Zeiss LSM880, Germany) was employed on optically polished surfaces to detect sub-surface pores, which appear as bright spots against a dark background under the CLSM imaging mode. The spatial resolution of the CLSM system was estimated to be roughly <100 nm. For the CLSM analysis, a series of optical slices (each ~$100 \times 100\ \mu m^2$) were acquired at a depth interval of 400 nm. Multiple slices (acquisition time: 30 s per slice) were then reconstructed to generate a three-dimensional volume of $100 \times 100 \times 2\ \mu m^3$, enabling a volumetric inspection for pores within the detection limit of the instrument. (Line 26, Page 13 to Line 8, Page 14)

(2) The glass transition in the "B glass" is not obvious (Supp. Fig. 1). Also, is there a

second crystallization peak in this sample at around 950 ºC? More detailed characterization is needed.

Response: We thank the reviewer for this careful observation and the opportunity to clarify the thermal analysis data. The reviewer is correct to note the ambiguity in the original DSC curves. We acknowledge that the baseline features and the strong signal near 950 °C in the initial measurement likely arose from instrumental contamination, as the calorimeter has been used frequently for a variety of materials prior to this study.

In direct response, we have acquired the DSC curves using a newly installed and clean DSC instrument. The updated thermograms are presented in the revised Supplementary Fig. 1. As shown, the glass transition temperature for the B glass is now clearly defined. Furthermore, the previously noted broad feature near 950 °C is absent in the new measurement, confirming it was an artifact. The characteristic glass transition and primary crystallization temperatures for both B and BZ glasses remain consistent with our previously reported values. We believe that the new, clean DSC data provide a reliable and unambiguous basis for our thermal analysis and discussion.

(3) Why does the 11B MAS NMR data have so low signal-to-noise ratio? This is not typically observed in such samples. I was also surprised that the fitted boron coordination numbers were not reported.

Response: We thank the reviewer for raising these points, which allow us to clarify the experimental conditions and spectral interpretation. The relatively low signal-to-noise (S/N) ratio in our ^{11}B MAS NMR spectra is primarily due to the low concentrations of B_2O_3 in the sample (1.19 mol.% in B specimen and 1.35 mol.% in BZ specimen, as determined by ICP-OES analysis), which results in a weak signal. The spectra were acquired on a high-field spectrometer (14.09 T), and despite the lower S/N, the characteristic resonances for different boron coordination environment remain clearly discernible.

As indicated in Fig. 3, the broad signal located 20-5 ppm corresponds to trigonal [BO_3] units (shaded yellow), while the sharper peak around 0 ppm corresponds to tetrahedral $[BO_4]^-$ units (shaded green). These assignments are consistent with established literatures. Given that the two coordination states are well resolved in the spectra and the weak S/N ratio, deconvoluting the relative areas through peak fitting would not add significant additional insight for the current study. Furthermore, the coordination of boron in this multi-cation system is simultaneously influenced by both Na^+ and Ca^{2+}, which complicates the quantitative interpretation of site proportions. Therefore, we focused our discussion on the qualitative identification of both coordination types present, which confirms the dual role of boron as a network former and modifier in the glass structure. We acknowledge that higher B_2O_3 content or longer acquisition times could improve the S/N ration, but the present data adequately support the key structural conclusion regarding boron incorporation. In the revised Fig. 3, the key structural units have been clearly labeled.

(4) The assignment of the crystal phase based on PDF card 79-1085 seems inconsistent with all the peaks in the crystallized samples (Fig. 4a). Specifically, the authors' samples have a relatively strong peak at around 61-62 degrees, which does not appear in the PDF card.

Response: We thank the reviewer for this careful observation regarding the XRD pattern. We appreciate the opportunity to clarify this point. First, we confirm thar the primary crystalline phases in our samples is consistent with the reference structure corresponding to PDF#79-1085 ($Na_4Ca_4Si_6O_{18}$). This assignment is strongly supported by the Rietveld refinement of the diffraction pattern, which yields good reliability factors (R_{wp} = 5.26%, R_p = 4.09%), as shown in Fig. 2b of the manuscript.

Regarding the peak near 61-62°, the reference PDF card (79-1085) does indeed list several peaks in this angular region, including reflections at approximately at 60.34°, 60.5°, 60.62°, 60.78°, 61.99°, and 62.16°. In our samples, the observed strong peak around 61-62° likely corresponds to a subset of these closely spaced reflections. The relative intensities and exact positions of these peaks can be influenced by cationic substitutions within the crystal structure. In the BZ ceramic, Zr^{4+} partially substitutes for Ca^{2+} on the M(4) site, while Na^+ may also occupy some Ca^{2+} sites (Fig. 5c) could change the relative diffraction peak intensities and even small shift in diffraction peaks. Such substitutions can alter lattice parameters, cause slight peak shifts, and modify relative diffraction intensities compared to the ideal reference pattern. Therefore, the observed diffraction profile, including the prominent feature near 61-62°, is fully consistent with a Zr-modified solid solution based on the $Na_4Ca_4Si_6O_{18}$ structure, rather than indicating a different phase.

(5) The authors refer to their samples as "crack-free" but only minimal mechanical characterization of the samples is reported. Most importantly, fracture toughness testing should be performed (using a self-consistent method, i.e. not indentation) to confirm that the samples have the required damage tolerance for the proposed applications, such as "transparent armor". Comparison of the mechanical properties (including hardness and bending strength) should also be done to other ceramics and glass-ceramics.

Response: We sincerely thank the reviewer for these critical and constructive comments, which have greatly helped to clarify our claims and significantly strengthen the mechanical characterization in our manuscript. We have thoroughly revised the text and added new data in response.

First, we apologize for the unclear use of the term "crack-free". Our intended meaning was that the reduction in thermal expansion anisotropy minimizes internal stresses during crystallization, thereby preventing the formation of microcracks that typically degrade transparency in highly crystalline Na-Ca-silicate systems, as documented in prior studies (e.g., Berthier et al., Journal of Non-Crystalline Solids, 208, 254, 1721; Mastelaro et al., Journal of Non-Crystalline Solids, 1996, 194, 297). For clarity, we have removed the absolute term "crack-free" from the revised manuscript.

Second, we agree with the reviewer that the term "transparent armor" was used

without sufficient mechanical justification. The BZ ceramic reported here are not optimized for such high-impact applications. Consequently, we have removed the reference to "transparent armor" from the manuscript.

Most importantly, following the reviewer's recommendation, we have performed comprehensive mechanical testing using standard methods. Facture toughness was measured via the sing-edge precracked beam (SEPB) method (ASTM C1421), a self-consistent technique, yielding a value of 1.5 ± 0.2 $MPa \cdot m^{1/2}$ for the BZ ceramic, which represents a significant improvement over the BZ glass (0.8 ± 0.3 $MPa \cdot m^{1/2}$). Young's modulus, measured by impulse excitation technique (ASTM E1876), increased from 88.8 ± 0.2 GPa to 120.1 ± 0.5 GPa. These new data are now reported in the main text. Furthermore, we have added a comparative analysis of Vickers hardness and three-point bending strength against other classes of transparent materials, including commercial transparent ceramics, transparent glass-ceramics, and bioactive ceramics, in Supplementary Fig. 11. While the strength of the BZ ceramic does not reach that of high-performance engineering ceramics, its Vickers hardness surpasses that of many bioactive ceramics, highlighting a clear mechanical advantage for potential biomedical applications. The detailed experimental methods for fracture toughness, Young's modulus, and hydrolytic stability testing have been added to the "Property measurements" section.

We believe these revisions address the reviewer's concerns directly, replace overstatements with precise descriptions, and provide a robust, comparative mechanical property dataset that solidifies the value proposition of our material for its most promising applications.

In the revised manuscript, the following content was added.

This trend extends to fracture toughness, which rises from 0.8±0.3 $MPa \cdot m^{1/2}$ (BZ glass) to 1.5±0.2 $MPa \cdot m^{1/2}$ (BZ ceramic), and Young's modulus, which increases from 88.8 ± 0.2 GPa (BZ glass) to 120.1 ± 0.5 GPa (BZ ceramic). (Line 22-25, Page 9)

A comparative analysis of Vickers hardness and three-point bending strength among conventional transparent ceramics, glass-ceramics, and bioactive ceramics[32-39] (Supplementary Fig. 11) further contextualizes this performance. While the BZ ceramic does not reach the strength levels of conventional transparent ceramics, its Vickers hardness is higher than that of typical bioceramics, highlighting distinct advantages for biomedical use. (Line 26, Page 9 to Line 4, Page 10)

In the property measurements section, the following content was added.

Fracture toughness was measured using the single-edge precracked beam (SEPB) method in accordance with ASTM C1421-99. Five bar specimens with dimensions of 25 mm × 4 mm × 3 mm were tested using a universal testing machine (5967, Instron, USA). Young's modulus was determined by the impulse excitation technique (ASTM E1876-22) using an IET-01 instrument (Zhuosheng, China) on rectangular specimens (55.8 mm × 14.8 mm × 2.6 mm). (Line 22-28, Page 14)

(6) Similarly, to evaluate the samples' potential for biomedical applications, specifically

the comparison to 45S5 Bioglass, requires testing of their ability to precipitate hydroxyapatite.

Response: We sincerely thank the reviewer for this important suggestion, which has significantly strengthened the biomedical evaluation in our work. In direct response, we have conducted comprehensive in-vitro bioactivity tests by immersing the BZ ceramic specimen in simulated body fluid (SBF) for 7 days at 37 °C. The formation of hydroxyapatite (HA) on the material surface was robustly confirmed through a combination of analytical techniques, and these new findings have been integrated into the revised manuscript. The experimental details are now included in the "In-vitro cytotoxicity and bioactivity assessment" section, while the results are presented and discussed with new Fig. 6c, d and Supplementary Figs. 12 and 13.

Specifically, Fourier-transform infrared spectroscopy in attenuated total reflectance mode (ATR-FTIR) revealed characteristic peaks of phosphate (PO_4^{3-}) groups at 1087, 1026, 962, and 600 cm^{-1}, indicative of HA formation[40,41]. This was corroborated by XRD patterns, which clearly displayed the characteristic peaks of crystalline HA. Furthermore, FE-SEM showed a dense layer of newly formed particles covering the sample surface after immersion. EDS mapping confirmed the co-localized enrichment of Ca and P within these regions, providing direct elemental evidence for HA precipitation. This bioactive behavior is consistent with the reported properties of related calcium silicate crystalline phases (R. Du et al., J. Mater. Sci.-Mater. M., 2004, 15, 1285), thereby substantiating the bone-bonding potential of our BZ transparent ceramics and enabling a more meaningful comparison with established bioactive materials like 45S5 bioglass.

In the revised main text, the following content was added.

The bioactivity of the BZ ceramic was evaluated by examining its ability to induced hydroxyapatite (HA) formation in simulated body fluid (SBF). HA is a key biomaterial known to form direct bonds with bone tissue[40]. The appearance of the specimen after immersion was illustrated in Supplementary Fig. 12. ATR-FTIR spectra collected from the sample surface after 7 days of immersion (Fig. 6c) displayed characteristic absorption bands at 1087 cm^{-1}, 1026 cm^{-1}, and 600 cm^{-1}, which were attributed to phosphate (PO_4^{3-}) groups indicative of HA[40,41]. This finding was corroborated by XRD analysis (Fig. 6d), in which distinct diffraction peaks corresponding to crystalline HA were clearly observed. Furthermore, FE-SEM observation (Supplementary Fig. 13) revealed a dense and continuous layer of newly formed particles covering the ceramic surface. EDS elemental mapping confirmed the co-localization of Ca and P within this layer (Supplementary Fig. 13), providing unequivocally evidence of HA precipitation. This bioactive behavior is consistent with the previously reported properties of related crystalline phases such as $Na_2Ca_2Si_3O_9$[42], substantiating the potential of BZ transparent ceramics for biomedical applications. (Line 11-26, Page 10)

In the "In-vitro cytotoxicity and bioactivity assessment" section, the following content was added.

The in-vitro bioactivity was assessed by immersing polished BZ ceramic specimens in simulated body fluid (SBF) at 37 °C for 7 days. The SBF was prepared with an ionic composition closely matching that of human blood plasma[46]. After immersion, the samples were thoroughly rinsed with deionized water and acetone. Surface precipitates were subsequently analyzed using Fourier-transform infrared (FTIR) spectroscopy in attenuated total reflectance (ATR) mode (iN10-iS50, ThermoFisher Scientific, USA), XRD, and FE-SEM coupled with EDS. (Line 1-7, Page 16)

(7) The chemical composition of the melt-quenched samples should be tested (e.g. using ICP-OES) as especially B2O3 and Na2CO3 are prone to evaporate at high temperature and B2O3 is very hygroscopic. Also, is it correct that the so-called "B glass" has zero B2O3 as stated in the Methods section (i.e. "x=0, y=0: B glass")?

Response: We thank the reviewer for raising these critical points regarding compositional accuracy and verification. The reviewer is absolutely correct to note the potential for volatilization of B_2O_3 and Na_2O during high-temperature melting, necessitating empirical verification.

In direct response to this comment, we have performed ICP-OES analysis on the as-annealed glasses. The resulting compositions (in mol.%) are:

B glass: 48.57 SiO_2 – 32.61 CaO – 17.63 Na_2O – 1.19 B_2O_3

BZ glass: 47.49 SiO_2 – 32.21 CaO – 17.26 Na_2O – 1.69 ZrO_2 – 1.35 B_2O_3

This data has been added to the revised Materials and preparation section. The results confirm the effective retention of the key components.

We sincerely apologize for the error in the original description. The "B glass" indeed contains B_2O_3. The corrected nominal composition in the Methods and preparation is now x=0, y=2 (i.e., 2 mol.% B_2O_3).

We believe the inclusion of the verified ICP-OES data and the corrected compositional description significantly enhance the experimental rigor and reproducibility of our study.

In the "Materials and preparation" sections, the following sentences were added:

To ensure the compositional accuracy, the as-annealed glasses were analyzed by inductively coupled plasma-optical emission spectrometry (ICP-OES, Prodigy 7, LEEMAN, USA). The measured compositions (in mol.%) were 48.57 SiO_2-32.61 CaO-17.63 Na_2O-1.19 B_2O_3 for the B glass and 47.49 SiO_2-32.21 CaO-17.26 Na_2O-1.69 ZrO_2-1.35 B_2O_3 for the BZ glass. Both glasses show a slight enrichment in Na_2O relative to the stoichiometric $Na_2O{\cdot}2CaO{\cdot}3SiO_2$ formulation, with Si/Ca molar ratios close to 1.5. This deliberate off-stoichiometry was adopted to stabilize the high-temperature polymorph and to tailor the glass-forming ability and crystallization, thereby enabling the fabrication of transparent ceramic. (Line 25, Page 11 to Line 5, Page 12)

Reviewer #3 (Remarks to the Author):

This study represents a substantial advance in transparent ceramics research by demonstrating a melt-forming approach to fabricate centimeter-scale sodium calcium silicate transparent ceramics—a longstanding challenge for alkali-containing systems. The work holds significant applied value through its utilization of industrial glass manufacturing techniques to achieve unprecedented specimen dimensions (11.3 cm ×6.5 cm) while maintaining dual functionality (biocompatibility and fire resistance). Academically, it provides novel insights into crystallization control in alkali glasses through strategic Zr^{4+} substitution, establishing a new paradigm for anisotropy reduction via crystallographic site engineering. The correlation between structural modification and functional properties offers valuable design principles for advanced optical materials.

I recommend Minor Revision to enhance the manuscript's impact and clarity. The study is well-conceived and experimentally thorough, but would benefit from the following specific improvement:

(1) The reasons for selecting a nucleation temperature of 600 ℃ and a crystallization temperature of 700 ℃ for 20 h for B glass, as well as a nucleation temperature of 620 ℃ and a crystallization temperature of 720 ℃ for 20 h for BZ glass. Meanwhile, please reconfirm whether the nucleation temperature for BZ glass is 620 ℃ or 630 ℃ to ensure consistency with the temperature indicated in the legend.

Response: We sincerely thank the reviewer for this important observation and the opportunity to clarify our thermal treatment strategy. The reviewer is correct to point out the inconsistency. There was an error in the originally stated nucleation temperature for BZ glass. The accurate and consistent values are: 630 °C for nucleation and 720 °C for crystallization for BZ glass, as indicated in the figure legend. We have corrected this typo in the revised manuscript (marked red) and sincerely apologize for any confusion caused.

(2) Please standardize the graph preparation by using different colors to distinguish Q2(1), Q2(2), and Q2(3) in Fig. 3c, and provide an explanation for the meaning of Qn.

Response: We appreciate the reviewer's suggestion to enhance the clarity of our spectroscopic data presentation. We have revised Fig. 3c in accordance with the comment. The deconvoluted peaks corresponding to Q^2 silicate species (specifically $Q^2(1)$, $Q^2(2)$, $Q^2(3)$) in Fig. 3c are now distinctly represented using three different colors for immediate visual differentiation. All Q^n species are also clearly marked in the revised Fig. 3b and 3c.

Regarding the meaning of Q^n, it is the standard notation in silicate glass/ceramic spectroscopy, where n (ranging from 0 to 4) represents the number of bridging oxygen atoms per $[SiO_4]$ tetrahedron. In our study, the dominance of Q^2 species in the spectra confirms the formation of the targeted ring-silicate structure ($Na_4Ca_4Si_6O_{18}$), which is consistent with our structural model. Specifically, $Q^2(i)$ (i=1, 2, 3) denotes three distinct sets of Q^2 species within the six-membered silicate ring. To ensure clarity, a full explanation of Q^n and $Q^2(i)$ notation has been added to Supplementary Table 1.

We believe these modifications improve the figure's interpretability and mark the

manuscript more accessibility to a broader readership in materials science.

(3) The image results for (iv) 2 h and (v) 4 h in Fig. 4b are similar. It is recommended to replace (v) with the image for 20 h.

Response: We thank the reviewer for this attentive suggestion to optimize the figure representation. We agree that images (iv) and (v) in the original Fig. 4b depict similar microstructural stages. Regarding the suggestion to include the 20-hour sample, we would like to clarify that a representative microstructure image of the sample crystallized for 20 hours is already presented in Fig. 2d of the manuscript, where it serves to illustrate the final structure achieved under the optimized thermal schedule. To avoid redundancy and maintain the logical flow of each figure (Fig. 4b focuses on the kinetic evolution of microstructure over shorter timescales), we have chosen to retain the current selection for Fig. 4b. We hope this explanation clarifies our figure arrangement, and we appreciate the reviewer's understanding.

(4) Provide full UV-Vis-NIR transmission spectra rather than single-wavelength data to better evaluate optical performance for practical applications.

Response: We thank the reviewer for this constructive suggestion. Following the recommendation, we have extended our optical characterization to include full-range transmission spectra. The complete UV-Vis-NIR spectra (250-2000 nm) of BZ ceramics are now provided as Supplementary Fig. 6, offering a comprehensive assessment of its optical performance across the relevant wavelength range. To maintain clarity in presenting the key visible-range transparency that is central to our discussion, the original figure focusing on this region (Fig. 4c) has been retained in the revised manuscript. Together, these figures provide both a detailed view of the visible performance and a complete spectral profile, significantly strengthening the manuscripts optical analysis and its relevance to practical applications.

In the revised manuscript, the following content was added.

This microstructural progression directly modulated optical transmittance (Fig. 4c, d, and Supplementary Fig. 6) (Line 12-13, Page 7)

The manuscript is generally well-written and the data support the conclusions. The combination of scalability, dual functionality, and fundamental mechanistic insight makes this work highly suitable for Nature Communications. With the requested minor revisions, this study will provide a valuable contribution to the materials science community and offer tangible pathways for industrial translation.

Response to Reviewers

Reviewer #1 (Remarks to the Author):

The authors have made a subsequent effort to answer the reviewers' remarks. To me there is one major point/question remaining: should the sample be named a ceramic or a glass-ceramic?

To me this question can be answered by referring to the 2018 JNCS paper cited by the authors: if the authors can observe some remaining glass in their sample then it is a glass-ceramic and if there is no more glass in the sample (full crystallisation process) then it is a ceramic. I would not refer to the book from the 1960s cited by the authors in their response letter (characterisation tools have much developed since then). At the bottom of page 14 of their response letter they claim that both GC an C materials can contain glass. I don't agree with that and this is in contradiction with the recent definition of a GC.

Also a material cannot be defined depending on the process used to prepare it (behind is the idea that a glass-cermaic sample should be elaborated by crystallisation from glass). There is a simple reason for this: if you find a material (in nature for example) and have no idea how it was processed then you cannot name this material. Also the JNCS 2018 paper mentions GC materials elaborated differently.

The 2018 JNCS paper managed to reach a consensus between many academic researchers and industrials regarding the definition of a GC (TC7 committee), I reckon this is the definition to use today.

Now, is there or not glass in the authors' sample? From electron microscopy it seems that there is not. I understand that the authors also performed quantitative amorphous phase content by XRD but really I would not trust much this approach as it is not precise, especially in this case where the authors did not determine exactly the population on each crystallographic site.

If the authors stand on saying that there is glass in their sample then they must change their claims regarding "largest ceramic sample prepared..." etc. as compared to existing glass-ceramic sample (see exmaple cited by reviewer 2) their sample is not any larger or more transparent.

I hope these comment can help to further improve the manuscript.

Response:

We thank the reviewer for this definitive and highly constructive guidance. We fully accept the reviewer's position that the 2018 JNCS consensus definition (Deubener et al., J. Non-Cryst. Solids, 2018, 501, 3-10) should be adopted as the current standard. According to this definition, a material derived from controlled glass crystallization that contains any residual glass phase is properly termed a glass-ceramic. Our BZ material contains ~7 wt% residual glass (as quantified by Rietveld refinement, albeit with the acknowledged limitations), and therefore, we have **systematically changed the terminology throughout the manuscript from "ceramic" to "glass-ceramic".** Following the reviewer's suggestion, the title was changed into "Scalable fabrication of transparent sodium calcium silicate glass-ceramics by structural engineering overcoming anisotropy barriers". Correspondingly, we have revised the abstract, main

text, and all figure captions. All claims regarding "record-sized" or "largest ceramic" have been removed or explicitly qualified as "large SCS glass-ceramic". All the changes are highlighted in red in the revised manuscript.

We are deeply grateful for the reviewer for helping us align with the internationally accepted nomenclature and for the sustained effort to improve our manuscript.

Reviewer #2 (Remarks to the Author):

I thank the authors for their responses and revisions. For me, the conceptual novelty is still much too limited to warrant publication in Nature Communications. It was also very hard for me to follow the authors' logic in their response regarding glass-ceramic vs. ceramic. They state that "Given the presence of a glassy phase, the BZ "ceramic" should indeed be termed as a "glass-ceramic".", but still insist to refer to it as a ceramic… If a ceramic phase contains a residual glass phase, it is per definition a glass-ceramic, i.e., it is incorrect to state that "both material classes can contain a glass phase". Transparent glass-ceramics with better properties, larger sizes and made with similar raw materials are already fully established in the glass/ceramic literature and indeed already on the market. I think this alone proves my point of limited novelty, since the authors are not proposing a new technique/concept to produce such materials and microstructures, and they do not offer any advantages compared to the existing materials. Even if we follow the authors' argument that the novelty comes because the present materials are "ceramics", this does not make it novel since the improved performance is still due to the same/similar microstructures as can and have been induced in glass-ceramics and formed by the same process of heat-treatment.

Minor point: regarding the SEPB testing, the authors provide only very minimal details on how these experiments were done. Please show images of the bridge compression setup (stiffness of this is setup is e.g. crucial), indentations used to make precrack, precrack front variation, and load-displacement curves.

I think the work is of interest to other researchers working on glass-ceramics and deserves publication in a materials journal focusing on glasses and ceramics, such as Journal of the American Ceramic Society or Acta Materialia.

Response:

We thank the reviewer for the continued rigorous assessment of our manuscript and for the valuable time and expertise invested. We appreciate the critical comments, which have helped us to further improve the clarity and accuracy of our work. Below, we address the remaining concerns point by point.

1. Terminology: "ceramic" has been changed to "glass-ceramic"

We full accept the reviewer's position that the 2018 JNCS consensus definition (Deubener et al., J. Non-Cryst. Solids, 2018, 501, 3-10) should be the current standard. According to this definition, a material derived from controlled glass crystallization that contains any residual glass phase is properly termed a glass-ceramic. Our BZ material contains approximately 7 wt% residual glass (as quantified by Rietveld refinement). Therefore, we have **systematically changed the terminology throughout the manuscript from "ceramic" to "glass-ceramic"**. Following the suggestion, the title was changed into "Scalable fabrication of transparent sodium calcium silicate glass-

ceramics by structural engineering overcoming anisotropy barriers". Correspondingly, we have revised the abstract, main text, and all figure captions. All claims referring to "record-sized" or "largest ceramic" have been removed or explicitly qualified as "large SCS glass-ceramic". All changes are highlighted in red in the revised manuscript. We thank the reviewer for helping us align with the internationally accepted nomenclature.

2. Novelty and suitability for *Nature Communications*

We respectfully acknowledge the reviewer's concern regarding conceptual novelty and the comparison with existing commercial glass-ceramics. We have carefully reconsidered our position and wish to explain why we believe the work is still meets the high standards of Nature Communications, with fully respecting the reviewer's viewpoint.

The reviewer correctly notes that transparent glass-ceramics with excellent properties exist in other compositional systems (e.g., lithium aluminosilicates). Our claim of novelty is **system-specific**: we address the **sodium calcium silicate (SCS) system**, which account for >80% of global glass production but has remained notoriously difficult to crystallize into a crack-free, highly transparent monolith for nearly two decades. We respectfully summarize the core innovations as follows:

First crack-free, highly crystalline (>90 wt%) transparent glass-ceramic from SCS glass. Since the work of Berthier et al. (2008), it has been known that increasing the crystallinity of SCS glass beyond ~97 vol% leads to severe microcracking and loss of transparency due to thermal expansion anisotropy (Berthier et al., 2008). Our work demonstrates that this barrier can be overcome by a targeted Zr^{4+}/Na^{+} substitution strategy-a qualitative breakthrough in this specific, industrially dominant system that has resisted such progress for decades.

- **A new mechanistic principle: site-specific cation substitution reduces thermal expansion anisotropy by 42%**. We provide a detailed crystallographic mechanism linking Zr^{4+} substitution on the M(4) site and Na^{+} occupancy on the M(3) site to a quantified reduction in $\Delta\alpha$ ($\Delta\alpha$ reduced by 42%). The same substitution stabilizes the high-temperature polymorph up to ~910 °C. This structure-property design principle may be generalizable and offers a new strategy for mitigating anisotropy in ring-silicate system-a finding of broad interest to the materials science and community.
- **Scalable, industry-compatible processing**. Unlike most glass-derived transparent ceramics that require rapid quenching or specialized equipment, our process uses standard melt-forming (no rapid cooling) and conventional thermal treatments at ambient pressure. The precursor is a commercial SCS glass composition, enabling direct implement in existing production lines. The ability to produce centimeter-scale ($11.3\times6.5\times0.6$ cm^3) monolithic samples from the world's cheapest and most abundant glass is, we believe, an advance with immediate industrial relevance.
- **Multifunctional performance validated for emerging applications**. Our SCS glass-ceramics simultaneously achieves >84% (at 400 nm) visible transmittance (far exceeding previous SCS glass-ceramics with similar crystallinity), a phase transition temperature >910 °C, bioactivity (hydroxyapatite formation in SBF) and 100% cell viability (biomedical implant potential), and improved mechanical properties (Vickers

hardness, fracture toughness, Young's modulus). These properties open new application windows - biomedical implants, fire-resistant glazing - for the most abundant and inexpensive glass system in the world.

We respectfully argue that **solving a decades-old, composition-specific problem in the world's most commercially important glass system** – through a mechanistically novel and industrially scalable approach – represents a significant advance. *Nature Communications* has a strong track record of publishing papers that transform ubiquitous materials into high-performance platforms. We hope the reviewer will consider that the scientific and practical impact of this work, when judged within the context of the SCS system, meets the journal's criteria.

We are genuinely grateful for the reviewer's constructive criticism, which has forced us to sharpen our message and present our innovations more clearly. Should the editor and reviewer still deem the work more suitable for a specialized journal, we will respect that decision. Nevertheless, we hope that the revised manuscript, with all terminology corrected and novelty articulated, can now be considered for publication in ***Nature Communications***.

3. Additional experimental details for SEPB testing

We thank the reviewer for the valuable suggestion regarding the SEPB fracture toughness testing. In the revised manuscript, we have provided comprehensive experimental details to ensure full reproducibility and compliance with ASTM C1421. Specifically, we have added to the Supplementary Information:

- An image of the bridge-compression loading setup used from precrack introduction
- A representative optical image of the Vickers indentation imprints used to initiate the precrack
- Images showing the precrack front after stable propagation
- Typical load-displacement curves obtained during the SEPB test

All this information has been compiled as Supplementary Fig. 15, accompanied by the following description:

"As shown in Supplementary Fig. 15a, the upper part of the bridge compression fixture (in contact with the sample) is made of silicon carbide, and the lower part is made of high-carbon alloy steel; both components possess extremely high stiffness. Vickers indentations were used as efficient precrack precursors under a load of 1 kgf (~9.8 N) The load holding time were 10 s for the BZ glass sample and 20 s for the BZ-GC sample. Supplementary Fig. 15b displays the indentation lines on the BZ glass (left) and BZ-GC (right) samples, with magnified views of the indentation imprints provided as insets. Supplementary Fig. 15c,d show the precracks in the BZ glass and BZ-GC samples, respectively, after bridge compression.

A three-point bending fixture (identical to that used for flexural strength testing) was used to fracture the precracked samples at a cross-head speed of 0.2 mm/min. The bending span (S_0) was 28 mm. Supplementary Fig. 15e,f show the fracture surfaces after bending testing, where the precrack fronts are clearly visible. The average precrack lengths were 2.02 mm for the BZ glass sample (Supplementary Fig. 15e) and 1.90 mm for the BZ-GC sample (Supplementary Fig. 15f). Loading-displacement curves are

presented in Supplementary Fig. 15g. The maximum load (P_{max}) was 7.45 N for the BZ glass sample and 15.77 N for the BZ-GC sample. According to the calculation formulas specified in ASTM C1421-99, the fracture toughness values were approximately 0.7 MPa·m$^{1/2}$ for the BZ glass and 1.4 MPa·m$^{1/2}$ for the BZ-GC sample. Five specimens for each material were tested, yielding average fracture toughness values of 0.8 ± 0.3 MPa·m$^{1/2}$ for BZ glass and 1.5 ± 0.2 MPa·m$^{1/2}$ for BZ-GC samples."

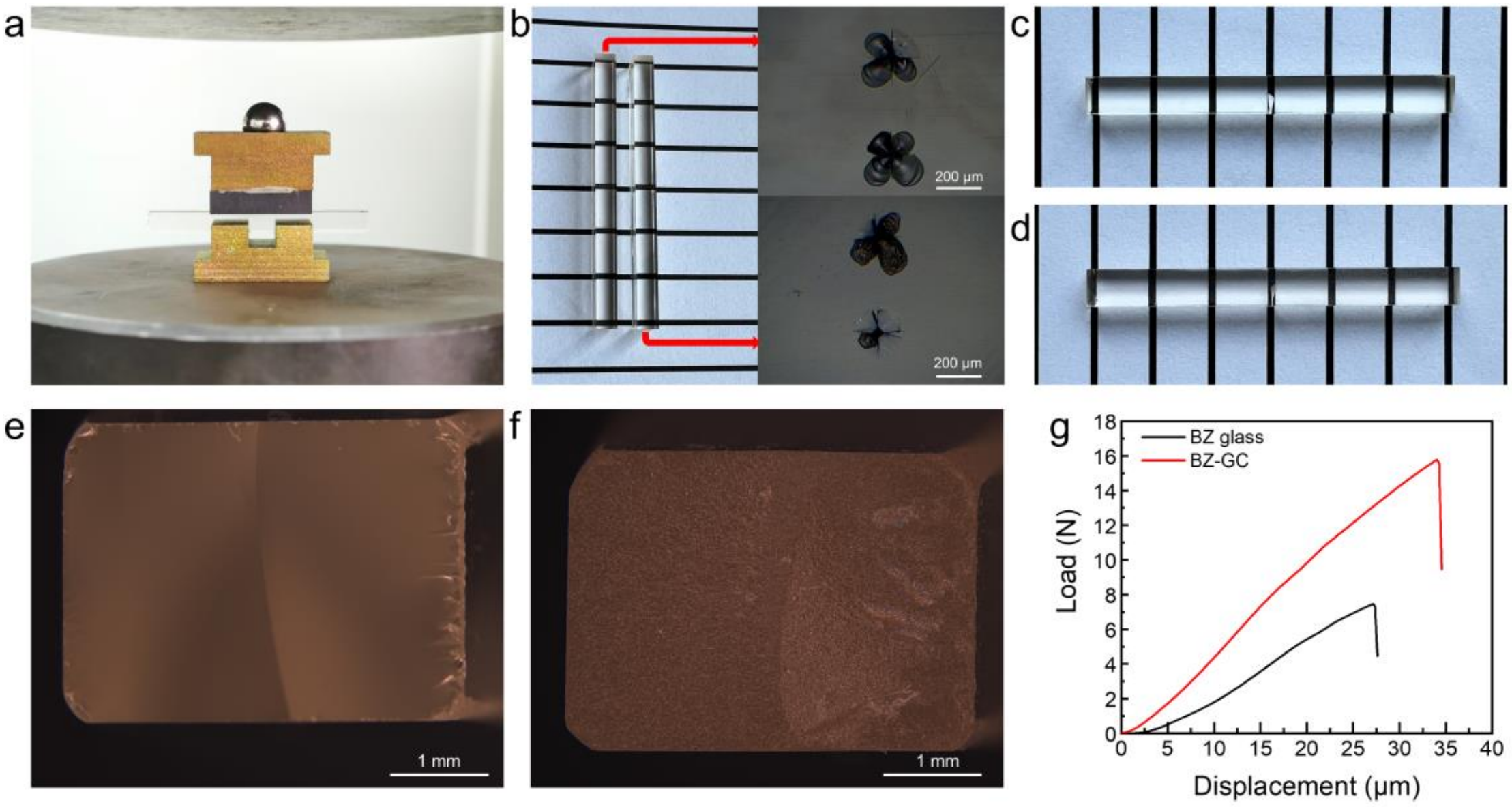

Supplementary Fig. 15. Detailed procedure of the single-edge precracked beam (SEPB) test. a Bridge compression fixture with the sample positioned between the upper (silicon carbide) and lower (high-carbon alloy steel) parts. **b** Vickers indentation lines on BZ glass (left) and BZ-GC (right) samples; the right insets show magnified views of the indentations produced under a load of 1 kgf (~9.8 N). **c, d** Precracks in the BZ glass (**c**) and BZ-GC (**d**) samples after bridge compression. **e, f** Fracture surfaces of the BZ glass (**e**) and BZ-GC (**f**) samples after three-point bending test with the precrack fronts clearly indicated. (**g**) Representative load-displacement curves recorded during the SEPB test for both materials.

We are deeply grateful to Reviewer #2 for the thorough and insightful evaluation of our manuscript. The reviewer's rigorous questions and constructive criticisms have significantly challenged us to clarify our terminology, refine our novelty claims, and strengthen our experimental documentation. Even though we respectfully maintain our position regarding the suitability of this work for Nature Communications, we genuinely appreciate the reviewer's perspective and the time invested in helping us improve the manuscript. The revisions made in response to these comments have substantially enhanced the clarity, completeness, and overall quality of the paper. We thank the reviewer sincerely for the invaluable guidance and for pushing us to present our work at its best.

Reviewer #3 (Remarks to the Author):

The authors have satisfactorily addressed all the comments and suggestions raised

during the review process. The revisions made have significantly improved the clarity and quality of the manuscript. Therefore, I recommend the manuscript for acceptance in its current form.

Response:

We are truly grateful to the reviewer for the thorough evaluation and the positive recommendation to accept the manuscript. We greatly appreciate the reviewer's recognition of our efforts in addressing all the comments and suggestions raised during the review process.

In response to the concerns raised by the other reviewers, we have made substantial revisions to the manuscript, which we believe have significantly improved its clarity, rigor, and overall quality. The key modifications include:

(1) Terminology revision: Based on the 2018 JNCS consensus definition, we have systematically changed the term “ceramic” to “glass-ceramic” throughout the manuscript, including the title, abstract, main text, and all figure captions. Claims regarding “record-sized” or “largest ceramic” have been removed or appropriately qualified.

(2) Novelty articulation: We have refined the presentation of the core innovations, emphasizing that this work represents the first successful fabrication of a highly crystalline (>90 wt%), crack-free, transparent glass-ceramic from commercial sodium calcium silicate (SCS) glass-the world's most abundant glass system. The mechanistic insight of reducing thermal expansion anisotropy by 42% through site-specific Zr^{4+}/Na^{+} substitution is now more clearly highlighted.

(3) Experimental additions: We have included detailed SEPB fracture toughness measurement (including setup schematics, indentation images, and load-displacement curves) to strengthen the manuscript.

We believe these revisions have substantially enhanced the manuscript and addressed all remaining concerns. We are deeply grateful for the constructive and encouraging feedback, and we thank the reviewer for supporting the publication of our work in its current form.

Response to Reviewers

Reviewer #1 (Remarks to the Author):

The authors have made appropriate modifications to the manuscript. I am now happy with the clarifications regarding the materials (glass-ceramic vs ceramic).

In the end the material developed here is very similar to the one reported by Berthier et al (2006). Could the authors comment on the requirement to use a nominal composition different from the aimed crystalline phase? I guess this composition difference explains the glass-ceramic nature of the material (full and congruent crystallisaiton is not possible). In a way developing a ceramic material would have been a clear progress and differentiation to Berthier's work.

Response:

We sincerely thank the reviewer for the positive recognition of our revisions and for raising this insightful point regarding the compositional design and its relation to the glass-ceramic nature of our material.

The reviewer correctly notes that our nominal composition deviates from the simple $Na_2O{\cdot}2CaO{\cdot}3SiO_2$ stoichiometry. This deviation was intentional and stems from two key considerations. First, to stabilize the high-temperature polymorph of $Na_4Ca_4Si_6O_{18}$, which exhibits substantially lower thermal expansion anisotropy compared to the low-temperature phase. The introduction of Zr^{4+} (partial substitution for Ca^{2+}) together with a non-stoichiometric Na/Ca ratio is essential for this stabilization. Second, the nominal composition was adjusted based on the melting trials and the actual glass composition verified by ICP-OES. The measured glass compositions show a light enrichment in Na_2O and Si/Ca molar ratio close to 1.5, which promotes homogeneous melting, improves glass stability, and ultimately enables the fabrication of a transparent, crack-free glass-ceramics after the prescribed heat treatment.

Regarding the reviewer's comment that the material developed here is similar to that reported by Berthier et al. (2006), we respectfully note a critical distinction. Berthier's work achieved by high crystallinity (~97 vol%) but suffered from severe microcracking and loss of transparency due to thermal expansion anisotropy. In contrast, our structural engineering strategy – specifically Zr^{4+} substitution and non-stoichiometric design – reduces the thermal expansion anisotropy, thereby suppressing microcracking even at comparable crystallinity. This enables a transparent glass-ceramic with >84% transmittance and centimeter-scale dimensions, which was not achieved in previous work.

Concerning the reviewer's point that developing a true ceramic (fully crystallized, no residual glass) would represent a clearer advance, we fully agree in principle. However, according to the updated definition of glass-ceramics (Deubener et al., J. Non-Cryst. Solids, 2018), any materials derived from controlled glass crystallization that contains a detectable residual glass phase should be terms a "glass-ceramic". Our material contains approximately 7 wt% residual glass, hence the revised terminology. Nevertheless, its microstructure – densely packed grains with only intergranular amorphous films – closely resembles that a sintered ceramic, and its properties

(transparency, mechanical strength, and thermal stability) significantly surpass those of Berthier's material. We believe that the scientific and technological advance lies in overcoming the long-standing anisotropy-induced microcracking problem, not in the semantic distinction between "glass-ceramic" and "ceramic".

We have incorporated the relevant discussion into the revised manuscript. Specifically, we have added the following passage (as already included in the previous version) to clarify the compositional design:

"To ensure the compositional accuracy, the as-annealed glasses were analyzed by inductively coupled plasma-optical emission spectrometry (ICP-OES, Prodigy 7, LEEMAN, USA). The measured compositions (in mol.%) were 48.57 SiO_2-32.61 CaO-17.63 Na_2O-1.19 B_2O_3 for the B glass and 47.49 SiO_2-32.21 CaO-17.26 Na_2O-1.69 ZrO_2-1.35 B_2O_3 for the BZ glass. Both glasses show a slight enrichment in Na_2O relative to the stoichiometric $Na_2O \cdot 2CaO \cdot 3SiO_2$ formulation, with Si/Ca molar ratios close to 1.5. This deliberate off-stoichiometry was adopted to stabilize the high-temperature polymorph and to tailor the glass-forming ability and crystallization, thereby enabling the fabrication of transparent and highly crystalline glass-ceramics."

We thank the reviewer again for the constructive critique, which has helped us sharpen the message of our work.

Reviewer #2 (Remarks to the Author):

I thank the authors again for their responses and revisions. My opinion remains that the conceptual novelty is too limited for publication in Nature Communications given the existing literature on transparent and tough glass-ceramics, including commercial products. However, at least the current manuscript is now much clearer, follows the correct terminology and some claims have been toned down.

Response:

We sincerely thank the reviewer for the continued engagement and for acknowledging the improvements made in the revised manuscript, particularly regarding clarity, correct terminology, and toned-down claims. We are grateful for the reviewer's rigorous assessment throughout the review process. We appreciated the reviewer's perspective and recognize that the current manuscript now presents the work in a more accurate and balanced manner. Thank you again for your valuable time and constructive suggestions.
